# Tissue fluidification promotes a cGAS–STING cytosolic DNA response in invasive breast cancer

**DOI:** 10.1038/s41563-022-01431-x

**Published:** 2022-12-29

**Authors:** Emanuela Frittoli, Andrea Palamidessi, Fabio Iannelli, Federica Zanardi, Stefano Villa, Leonardo Barzaghi, Hind Abdo, Valeria Cancila, Galina V. Beznoussenko, Giulia Della Chiara, Massimiliano Pagani, Chiara Malinverno, Dipanjan Bhattacharya, Federica Pisati, Weimiao Yu, Viviana Galimberti, Giuseppina Bonizzi, Emanuele Martini, Alexander A. Mironov, Ubaldo Gioia, Flora Ascione, Qingsen Li, Kristina Havas, Serena Magni, Zeno Lavagnino, Fabrizio Andrea Pennacchio, Paolo Maiuri, Silvia Caponi, Maurizio Mattarelli, Sabata Martino, Fabrizio d’Adda di Fagagna, Chiara Rossi, Marco Lucioni, Richard Tancredi, Paolo Pedrazzoli, Andrea Vecchione, Cristiano Petrini, Francesco Ferrari, Chiara Lanzuolo, Giovanni Bertalot, Guilherme Nader, Marco Foiani, Matthieu Piel, Roberto Cerbino, Fabio Giavazzi, Claudio Tripodo, Giorgio Scita

**Affiliations:** 1 IFOM, the FIRC Institute of Molecular Oncology, Milan, Italy; 2 Department of Medical Biotechnology and Translational Medicine, University of Milan, Segrate, Italy; 3 Department of Health Sciences, Human Pathology Section, University of Palermo School of Medicine, Palermo, Italy; 4 Institute of Molecular and Cell Biology, A*STAR, Singapore, & Bioinformatics Institute, A*STAR, Singapore, Singapore; 5 European Institute of Oncology (IEO) IRCCS, Milan, Italy; 6 Istituto Officina dei Materiali, National Research Council (IOM-CNR), Unit of Perugia, c/o Department of Physics and Geology, University of Perugia, Perugia, Italy; 7 Department of Physics and Geology, University of Perugia, Perugia, Italy; 8 Department of Chemistry, Biology and Biotechnology, Biochemical and Biotechnological Sciences, University of Perugia, Perugia, Italy; 9 Institute of Molecular Genetics, National Research Council, Pavia, Italy; 10 Unit of Anatomic Pathology, Department of Molecular Medicine, Fondazione IRCCS Policlinico San Matteo, University of Pavia, Pavia, Italy; 11 Medical Oncology Unit, Fondazione IRCCS Policlinico San Matteo, Pavia, Italy; 12 Department of Internal Medicine and Medical Therapy, University of Pavia, Pavia, Italy; 13 Department of Clinical and Molecular Medicine, University of Roma, La Sapienza, Rome, Italy; 14 Institute of Biomedical Technologies, National Research Council, Milan, Italy; 15 National Institute of Molecular Genetics Romeo and Enrica Invernizzi, INGM, Milan, Italy; 16 Department of Pathology, S. Chiara Hospital, Azienda Provinciale per i Servizi Sanitari, Trento, Italy; 17 CISMed University of Trento, University of Trento, Trento, Italy; 18 Institut Curie and Institut Pierre Gilles de Gennes, PSL Research University, CNRS, UMR-144, Paris, France; 19 Department of Oncology and Haemato-Oncology, University of Milan, Milan, Italy; 20 Faculty of Physics, University of Vienna, Vienna, Austria; 21 Present Address: Max Plank Institute for Dynamics and Self-Organization, Göttingen, Germany; 22 Present Address: Dipartimento di Medicina Molecolare e Biotecnologie Mediche, Università degli Studi di Napoli Federico II, Naples, Italy; 23 Present Address: S.C. Oncologia Medica, ASST Melegnano e della Martesana, Ospedale Uboldo, Cernusco sul Naviglio, Milan, Italy; 24 Present Address: Cell Pathology Children’s Hospital of Philadelphia, Research Institute Department of Pathology and Laboratory Medicine University of Pennsylvania Perelman School of Medicine, Philadelphia, PA USA

**Keywords:** Cellular motility, Biomaterials - cells, Cancer

## Abstract

The process in which locally confined epithelial malignancies progressively evolve into invasive cancers is often promoted by unjamming, a phase transition from a solid-like to a liquid-like state, which occurs in various tissues. Whether this tissue-level mechanical transition impacts phenotypes during carcinoma progression remains unclear. Here we report that the large fluctuations in cell density that accompany unjamming result in repeated mechanical deformations of cells and nuclei. This triggers a cellular mechano-protective mechanism involving an increase in nuclear size and rigidity, heterochromatin redistribution and remodelling of the perinuclear actin architecture into actin rings. The chronic strains and stresses associated with unjamming together with the reduction of Lamin B1 levels eventually result in DNA damage and nuclear envelope ruptures, with the release of cytosolic DNA that activates a cGAS–STING (cyclic GMP-AMP synthase–signalling adaptor stimulator of interferon genes)-dependent cytosolic DNA response gene program. This mechanically driven transcriptional rewiring ultimately alters the cell state, with the emergence of malignant traits, including epithelial-to-mesenchymal plasticity phenotypes and chemoresistance in invasive breast carcinoma.

## Abstract (web summary)

Tissue fluidification in invasive breast carcinoma is accompanied by mechanical stresses that compromise nuclear integrity and liberate DNA, resulting in the activation of a pro-inflammatory response that shape tumour evolution and progression.

## Main

The mechanical properties of cells and tissues are pivotal regulators of cell behaviour and fate in physiology and pathology, including during carcinogenesis^1^. Normal epithelial tissues frequently evolve into solid or jammed masses that are densely packed with cancer cells. To become malignant, a certain degree of fluidity is required for a tissue to be able to proliferate, migrate and disseminate. A recently discovered process by which cells can acquire migratory behaviour is cellular unjamming, a phase transition characterized by collective and cooperative cellular motion akin to fluid flow^2–6^. Whether and how unjamming impacts the acquisition of heritable changes that influence tissue state and malignant progression remains unclear.

Ductal adenocarcinoma in situ (DCIS), a precursor of invasive breast cancer, is a remarkable case in point. Firstly, DCISs typically grow at high cell density within the confinement of the mammary duct lumina (for example, comedonic growth)^7^. These conditions might expose DCIS to overcrowding and compressive mechanical stresses that impact their physical state favouring a transition to a solid (jammed) and kinetically arrested state^2,4,8^. Consistently, nearly 70% of DCISs are indolent, quasibenign lesions^9^. This suggests that packing and extreme confinement exert tumour-suppressive functions. However, 30% of these cancers overcome the caging imposed by the crowded cellular landscape of packed DCIS, by undergoing a solid-to-liquid (jammed–unjammed) phase transition, which facilitates the acquisition of cell locomotion and progression to invasive ductal carcinoma (IDC)^2^.

We hypothesize that this material-like phase transition is an adaptive response to mechanical challenging conditions that, in addition to promoting collective dissemination of early lesions, as previously shown^2^, would also coincidentally result in a long-term, cGAS–STING-mediated, transcriptional-dependent phenotype switch in invasive breast carcinoma.

## Tissue fluidification induces a cytosolic DNA response

The expression of the small G protein RAB5A, a pivotal regulator of endosome biogenesis upregulated in human breast cancer and associated with decreased disease-free survival^10^, is sufficient to overcome kinetic and proliferation arrest in densely packed epithelia^2,6^. RAB5A does so by triggering a mechanically driven phase transition from a solid (or jammed) and immobile state to a flocking-fluid, hyper-motile state that is analogous to animal flocking^2,6,11–13^. Molecularly, this is mediated by the endocytic function of RAB5A, which promotes the internalization of epidermal growth factor receptor (EGFR) into endosomal platforms for the prolonged activation of ERK1/2 and the actin nucleation promoting complex WAVE2. This, in turn, enhances lamellipodia that drive coordinated cell locomotion^2^. In breast carcinoma, tissue fluidification-via-flocking promotes collective motility and local invasiveness of DCIS^2^. We posit that this mechanically driven solid-to-fluid transition might also rewire the transcriptional state of early indolent lesions promoting a phenotypic switch that impacts tumour progression.

To address this possibility, we examine the transcriptional profile of densely packed epithelial monolayers formed by quasi-normal MCF10A cells and the respective oncogenic variant MCF10.DCIS.com cells. Both cell lines were engineered to express RAB5A in a doxycycline-inducible fashion to levels like those found in human breast cancer^2,10^. MCF10.DCIS.com cells express oncogenic T24-H-RAS and are used as models for the progression of DCIS to IDC^14^.

**Fig. 1:**
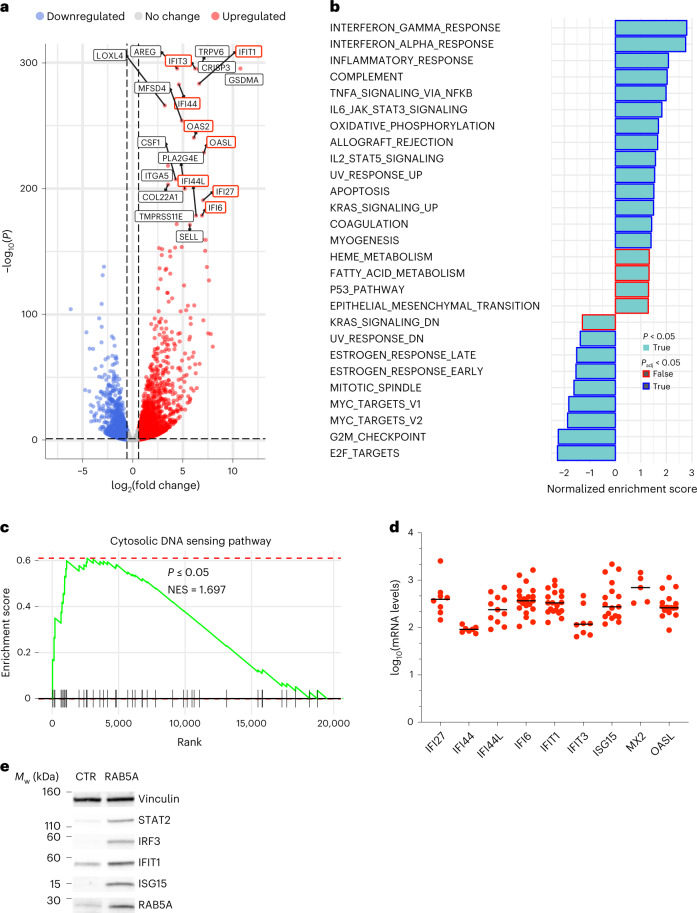
Tissue fluidification induces a CytoDR gene signature. **a**, Volcano plot of differentially expressed genes (DEG) in control and RAB5A-expressing MCF10.DCIS monolayers. All significantly RAB5A-expressing deregulated genes are in red (upregulated) and blue (downregulated). Enrichment (log_2_(fold change)) is plotted on the *x*-axis and significance (Wald test –log_10_(p-value two-sided)) is plotted on the *y*-axis. Labels are the most upregulated genes. Outlined in red are interferon-stimulated genes. **b**, GSEA of DEGs in RAB5A-expressing MCF10.DCIS.com monolayer over control cells. GSEA was performed using the Hallmarks pathway gene sets in the GSEA Molecular Signatures Database. Moderated *t*-statistic was used to rank the genes. Significantly enriched pathways are reported (one-sided *P* < 0.05) with the bar outline colour corresponding to the BH-adjusted *P* value. *P* values were calculated as the number of random genes with the same or more extreme enrichment score (ES) value divided by the total number of generated gene sets. **c**, GSEA enrichment plot of differentially expressed genes in RAB5A-expressing MCF10.DCIS.com monolayer using the KEGG (Kyoto Encyclopedia of Genes and Genomes) cytosolic DNA-sensing pathway (hsa04623). The green curve corresponds to the ES curve, which is the running sum of the weighted ES obtained from GSEA software, while the normalized ES (NES) and the corresponding one-sided *P* value are reported within the graph. **d**, Scatter plots of mRNA expression levels of *IFI27*, *IFI44*, *IFI44L*, *IFI6*, *IFIT1*, *IFIT3*, *ISG15*, *MX2* and *OASL* determined by qRT-PCR in RAB5A-expressing MCF10.DCIS monolayers relative to control cells. Data are expressed as log_10_ values, horizontal lines represent group medians. Each dot represents an independent experiment. Values were normalized to the controls of each experiment. **e**, Immunoblots of lysates from control (CTR) and RAB5A-expressing (RAB5A) MCF10.DCIS.com monolayers with the indicated antibodies (*n* = 3 independent experiments). *M*_w_ is indicated on the left. Source data

**Extended Data Fig. 1:**
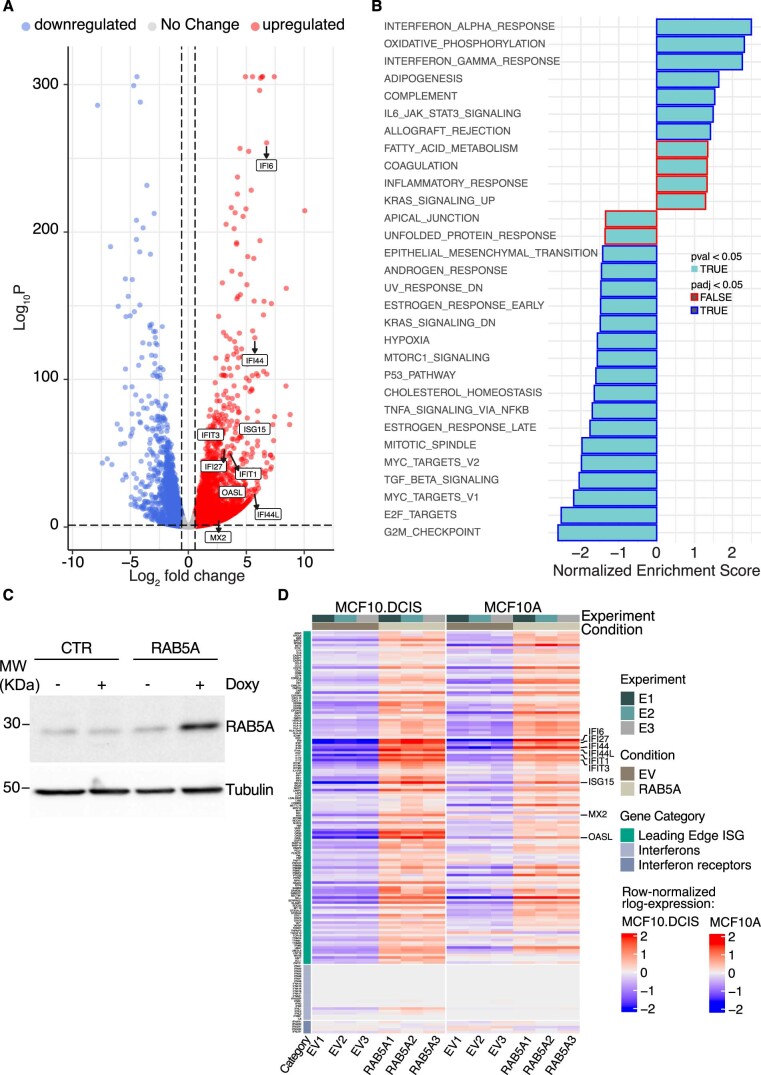
Tissue fluidification induces a CytoDR gene signature in MCF10 monolayers and has marginal effect on Interferon and Interferon receptor genes. **a**. Volcano plot of DEG between control empty vector and RAB5A-MCF10A monolayers. All significantly RAB5A-deregulated genes are indicated in red (upregulated) and blue (downregulated). The enrichment (log2Fold Change) is on the x-axis and the significance (Wald test -log10 p value two-sided) is on the y-axis. Labels show genes validated in Fig. 1d. **b**. Gene set enrichment analysis (GSEA) of DEG in RAB5A-MCF10A monolayer over control cells. Moderated t-statistic was used to rank the genes. Significantly enriched pathways (one-sided P-value < 0.05) are with the colour of the outline of the bar corresponding to the BH-adjusted P-value. P-value was calculated as the number of random genes with the same or more extreme ES value divided by the total number of generated gene sets. **c**. Immunoblots of lysates from doxycycline-treated control (CTR) and RAB5A-(RAB5A)-MCF10.DCIS.com monolayers in 1 experiment out of n = 4 independent ones with the indicated antibodies. Mw is on the left. **d**. Heatmap of the row-normalized expression level of leading-edge genes for the interferon alpha and gamma response pathways, the interferon and interferon receptor genes in each of the 3 replicates of control empty vector (EV) and RAB5A-expressing (RAB5A)-MCF10.DCIS and MCF10A monolayers. Magnified labels on the right indicate genes validated in Fig. 1d. Normalized expression has been calculated for each gene starting from DeSeq2 regularized log-transformed expression value and then normalized by row mean values. Source data

**Extended Data Fig. 2:**
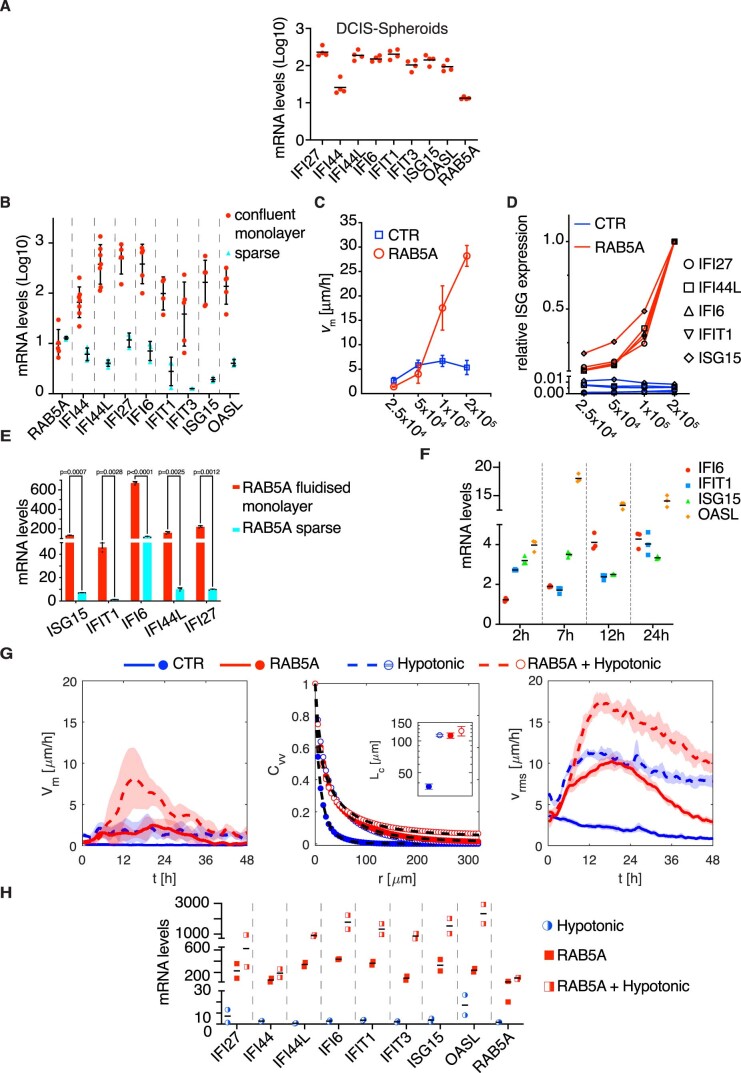
Tissues fluidification induces CytoDR gene expression in various normal and tumoral mammary epithelia. **a**. mRNA expression levels of IFI27, IFI44, IFI44L, IFI6, IFIT1, IFIT3, ISG15, OASL and RAB5A in RAB5A-MCF10.DCIS.com spheroids over the respective control. Data are the mean (n = 3 independent experiments). **b**. mRNA expression levels of RAB5A, IFI44, IFI44L, IFI27, IFI6, IFIT1, IFIT3, ISG15 and OASL in RAB5A-MCF10.DCIS.com monolayers or sparsely seeded cells (sparse) over the corresponding control cells. Data are the mean ± s.d. (*n* = 3 independent experiments). In **a**. and **b**., Values were normalized to the controls of each experiment. **c**. Average migration speed, *v*_*m*_, of control (CTR) or RAB5A-(RAB5A)-MCF10.DCIS.com cells seeded at various densities, and monitored by time lapse (Supplementary Video 1). The values were obtained by PIV analysis evaluated over the time window 8–40 h. Data are the mean ± s.d. (*n* = 3 independent experiments). **d**. Relative expression levels of the indicated ISG genes of control (CTR) or RAB5A- (RAB5A)-MCF10.DCIS.com cells seeded at various densities as described in **c**. Data are the mean (*n* = 3 independent experiments). Values were normalized to the controls at each dilution and expressed as a relative expression with respect to the maximal level for each ISG at the highest seeded density. **e**. mRNA expression levels of the indicated genes in control- and RAB5A-cells seeded at jamming density (fluidized monolayer RAB5A). The same cells were subsequently replated sparsely in the continuous presence of doxycycline to ensure the maintenance of the transgene expression before extracting RNA. Data are the mean ± s.d. (*n* = 3 independent experiments). Values were normalized to the controls of each experiment, unpaired two-tailed t-test with Welch’s correction. **f**. mRNA expression levels of IFI6, IFIT1, ISG15 and OASL in MCF10.DCIS.com monolayers seeded at jamming density incubated or not with hypotonic medium (composed of a 1:1 mix of media and H_2_O + 1 mM CaCl_2_ + 1 mM MgCl_2_) at the indicated time points. Data are expressed as mean (n = 3 independent experiments). Values were normalized to the controls of each experiment. All values are significantly different with a p value less than 0.05, each-pair two tailed Student’s t-test. **g**. Migration parameters, obtained from PIV, of control (CTR) or RAB5A-(RAB5A)-MCF10.DCIS.com monolayers treated with hypotonic media for 48 h (Supplementary Video 2), as in 2f. Left panel: migration speed *v*_*m*_. Central panel: spatial velocity correlation function *C*_*vv*_ evaluated over the time window 8–40 h. In the inset are reported the corresponding correlation lengths *L*_*c*_ obtained from stretched exponential fits on *C*_*vv*_ curves (black dashed lines in the main panel). Right panel**:** root mean square velocities in the center of mass reference frame. Velocity fields are computed over 4 FOVs per condition (each FOV including more than 2 × 10^3^ cells). Data are averages over different FOVs (right and left panel) and different time intervals (central panel). Shadowed error bars in left and right panel are evaluated as the standard deviation over the different FOVs. Error bars in central panel are the standard deviation of the mean over 5 different time intervals. **h**. mRNA expression levels of IFI27, IFI44, IFI44L, IFI6, IFIT1, IFIT3, ISG15, OASL and RAB5A in MCF10.DCIS.com monolayers subjected to different conditions (doxycycline-induction of RAB5A-expression, hypotonic treatment or the combination of both) over control cells. Data are the mean (*n* = 2 independent experiments). Values were normalized to the controls of each experiment. Source data

As expected, densely packed MCF10A and MCF10.DCIS.com monolayers are jammed and kinetically arrested^2,6^. Induction of RAB5A promoted the reawakening of collective motion via flocking^2,6^. This was accompanied by robust alterations in the transcriptional profile (Fig. 1a and Extended Data Fig. 1a–c). Unexpectedly, gene set enrichment analysis (GSEA) revealed the interferon-stimulated gene signature (ISG) as the most significantly enriched in deregulated genes (Fig. 1b,c). Noticeably, innate immune responses are also promoted by free endogenous DNA present in the cytosol, which is recognized as nonself^15^. We thus verified that RAB5A expression boosted a cytosolic DNA response (CytoDR) program (Fig. 1c). Determination of the mRNA levels of the selected most upregulated genes confirmed the effect of RAB5A-fluidification, and highlighted the massive increase in the expression of a number of these genes (Fig. 1d,e). The upregulation of ISG was also detected in fluidized MCF10A monolayers (Supplementary Fig. 1a,b) and in MCF10.DCIS.com cells grown as tumoroid (Extended Data Fig. 2a). In all these conditions, we have previously shown that RAB5A expression is sufficient to promote a solid-to-liquid transition via flocking and persistent rotational collective motion^2,6^.

RAB5A upregulated CytoDR genes only mildly in sparse cells (Extended Data Fig. 2b), suggesting that this response is an emergent property of epithelial cell collectives and associated with tissue fluidification. To further explore this property, we correlated the expression of CytoDR genes and flocking motion (measured using the average migration speed *v*_m_ of the entire cell collectives) as a function of cell density. We found that above a critical density, which corresponds to a condition where cells form a system-spanning inter-connected cluster, there is a sharp increase in *v*_*m*_ (Extended Data Fig. 2c and Supplementary Video 1) and a concurrent elevation of CytoDR genes (Extended Data Fig. 2d). By contrast, CytoDR gene expression is diminished once RAB5A-expressing cells from compressed but flocking monolayers are replated sparsely (Extended Data Fig. 2e).

The induction of flocking motion via exposure to a hypotonic solution, which promotes tissues fluidification independently from RAB5A expression^6^, was sufficient to increase CytoDR gene expression (Extended Data Fig. 2f). Importantly, the concomitant expression of RAB5A and hypotonic treatment synergically activated flocking fluid motility, as revealed by the increase in typical quantities that measure collective motility, including the average migration speed *v*_m_, the velocity correlation length *L*_C_ and the root mean square amplitude of the velocity fluctuations *v*_rms_ (Extended Data Fig. 2g and Supplementary Video 2). The synergic increase in collective motility observed under these conditions also resulted in robust induction of CytoDR genes (Extended Data Fig. 2h).

**Extended Data Fig. 3:**
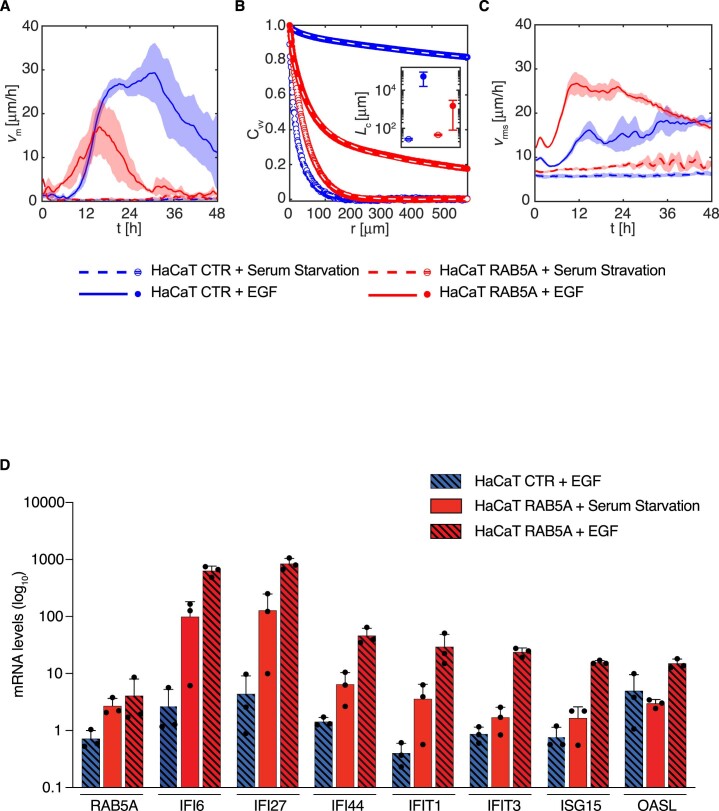
Tissue fluidification-dependent CytoDR is mediated by RAB5A in HaCat. **a–c**. Migration parameters of HaCat monolayers, obtained from nuclear tracking. Doxycycline-treated control or RAB5-expressing HaCat monolayers were serum starved (SS) for 48 h before addition of 100 ng/ml of EGF and monitored by time-lapse microscopy (Supplementary Video 3). (**a**) Migration speed *v*_*m*_. Data are averages over 5 FOVs for each condition. Shadowed error bars are evaluated as the standard deviation over the different FOVs. (**b**) Spatial velocity correlation functions *C*_*vv*_ evaluated over the time window 8–40 h. The inset report the corresponding correlation lengths *L*_*c*_ obtained from stretched exponential fits on *C*_*vv*_ curves (black dashed lines in the mail panel). Data are averages over 5 time intervals for each condition. Error bars are evaluated as the standard deviation of the mean over the different time intervals. (**c**) Root mean square velocities in the center of mass reference frame. Velocity fields are computed over different FOVs, encompassing in total about 10^4^ tracked cells per frame per condition. Data are averages over 5 FOVs for each condition. Shadowed error bars are evaluated as the standard deviation over the different FOVs.**d**. Box plot of the mRNA expression levels of RAB5A, IFI6, IFI27, IFI44, IFIT1, IFIT3, ISG15 and OASL in doxycycline-treated control (CTR) and RAB5A-HaCat monolayers treated exactly as described above with respect to control cells. Monolayers were serum starved mock-treated or stimulated with 100 ng/ml EGF: Hatched blue bar, serum starved HaCat CTR + EGF; Red bars, HaCat RAB5A- serum starved; hatched red bar, serum starved HaCat RAB5A stimulated with 100 ng/ml EGF (Serum Starved HaCat RAB5A + EGF) over control serum starved HaCat cells. Data are expressed as mean ± s.d. (*n* = 3 independent experiments). Values were normalized to the controls of each experiment. Source data

We also studied HaCat keratinocyte cells. These cells undergo flocking after induction of RAB5A^6^, which is greatly enhanced following the addition of EGF to quiescent, serum-starved cells^16^ (Extended Data Fig. 3a–c and Supplementary Video 3). EGF addition promoted robust flocking (Extended Data Fig. 3a–c) but it was insufficient to induce CytoDR genes. CytoDR gene induction required the concomitant expression of RAB5A (Extended Data Fig. 3a–c).

Together, these results indicate that endocytic-mediated tissue fluidization via flocking can transcriptionally rewire cell collectives toward a cytosolic DNA response in several normal and tumorigenic epithelia.

## Tissue fluidification activates a cGAS–STING pathway

cGAS is an innate immune sensor of DNA that recognizes cytosolic DNA, resulting in the activation of STING. STING, in turns, activates TANK binding kinase 1 (TBK1) to phosphorylate the transcription factor interferon regulatory factor 3 (IRF3), which translocates to the nucleus to induce the expression of type I/III interferon and interferon-stimulated genes^17^.

**Fig. 2:**
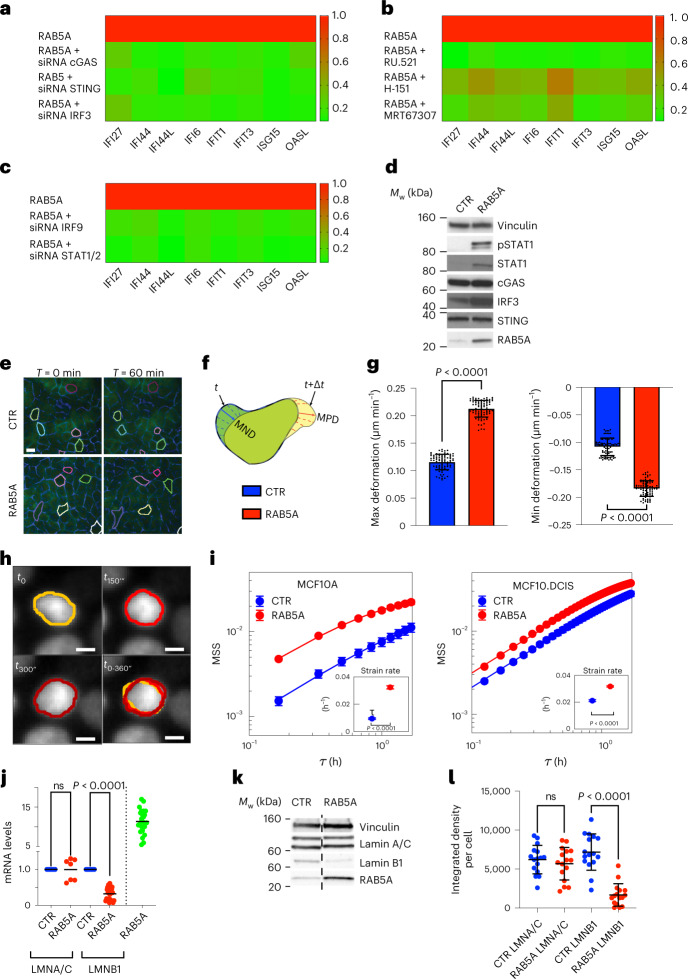
A cGAS–STING pathway mediates tissue fluidification-dependent CytoDR genes. **a**, Heatmap of CytoDR DEGs in RAB5A-MCF10.DCIS.com monolayers silenced for the indicated genes (Supplementary Fig. 1a). Data are the ratio of gene expression of each condition relative to mock-scramble-oligos-treated RAB5A-cells. The mean ± s.d. (*n* = 3 experiments); each-pair two-tailed Student’s *t*-test are in Source Data Fig. 2a. **b**, Heatmap of CytoDR DEGs in RAB5A-MCF10.DCIS.com monolayers treated with cGAS inhibitor RU.521(7 μg ml^–1^), or STING antagonist, H-151 (4 μg ml^–1^), or TBK1/IKK inhibitor, MRT67307 (20 μM). Data are the ratio of gene expression in each condition relative to vehicle-treated RAB5A-cells. The mean ± s.d. (*n* = 3 independent experiments), each-pair two-tailed Student’s *t*-test are in Source Data Fig. 2b. **c**, Heatmap of CytoDR DEGs in RAB5A-MCF10.DCIS.com monolayers silenced for the indicated genes. The data are the ratio of gene expression in each of conditions relative mock-scramble-oligos-treated RAB5A-cells. The mean ± s.d. (n = 7 experiments), each-pair two-tailed Student’s *t*-test are in Source Data Fig. 2c. **d**, Immunoblots of control- (CTR) and RAB5A-MCF10.DCIS.com monolayers with the indicated antibodies (*n* = 3 independent experiments). **e**, Still images of cell contours are indicated by pseudo-colouring of EGFP-CDH1, control- (CTR) and RAB5A-MCF10A cells (Supplementary Video 4). Scale bar, 10 μm. **f**, Scheme depicting cell maximum positive deformation (MPD) and negative deformation (MND). **g**, Data are the mean ± s.d. of MPD and MND (80 cells per condition in *n* = 4 independent experiments). Two-tailed Mann–Whitney non-parametric test. **h**, Consecutive frames (top-left, top-right and bottom-left subpanels) of the same RAB5A-expressing MCF10A nucleus (Supplementary Video 5). Continuous lines with different shades of red represent the profiles obtained via nuclear segmentations. In the bottom-right supanel is reported a superposition of the three profiles shown in the other subpanels. Scale bar, 5 μm. **i**, Comparison of the nuclear mean square strain (MSS) of control and RAB5A-MCF10A (left panel) or MCF10.DCIS.com (right panel) cell monolayers. The MSS is obtained by tracking and segmenting N nuclei over the time window 4–20 h (N > 5,000 and N > 1,000 for control- and RAB5A-MCF10A monolayers, respectively; >N700 and >N400 for control- and RAB5A-MCF10.DCIS.com monolayers, respectively). Continuous lines are best fitting curves to the data with an exponential model. Insets report the nuclear strain rate as mean ± s.d. (*n* = 10 randomly populated subsets of cells), two-tailed *t*-test. **j**, Levels of LMNA, LMNB1 and RAB5A mRNA in RAB5A- and control-MCF10.DCIS.com monolayers. The data are the mean (*n* = 7 for LMNA, *n* = 25 for LMNB1 *n* = 25 for RAB5A). Two-tailed Mann–Whitney non-parametric test. **k**, Immunoblots of control- and RAB5A-MCF10.DCIS.com monolayers with the indicated antibodies (*n* = 3 independent experiments). **l**. Scatter plot of the expression level of Lamin A/C and Lamin B1 in control and RAB5A-expressing MCF10.DCIS.com monolayers. Data are mean ± s.d. of the integrated density/cell measured in different FOV in *n* = 3 independent experiments, unpaired two-tailed *t*-test with Welch’s correction. *P* values are indicated in each graph. Source data

To determine the involvement of the cGAS–STING axis in the activation of CytoDR due to RAB5A-mediated tissue fluidification, we used pharmacological and molecular genetic loss-of-function approaches targeting each component of the cGAS–STING–TBK1–IRF3 pathway. We silenced *cGAS*, *STING* or *IRF3* or treated cells with the cGAS inhibitor, RU.521, the STING antagonist, H-151, or the TBK1/IKK inhibitor, MRT67307, which impairs the phosphorylation of IRF3 (ref. ^18^). All these treatments robustly hampered the upregulation of CytoDR genes induced by tissue fluidification in MCF10.DCIS.com model tissues (Fig. 2a,b and Supplementary Fig. 1a). We also targeted key transcription factors acting downstream of the cGAS–STING axis, *IRF9*, *STAT1* and *STAT2*, which robustly reduced CytoDR gene upregulation (Fig. 2c and Supplementary Fig. 1a). Immunoblotting of cellular lysates of densely packed monolayers revealed that IRF3 and both the total and phosphorylated levels of STAT1, were elevated (Fig. 2d), consistent with this pathway being activated by RAB5A-mediated fluidification of MCF10.DCIS.com cell collectives.

cGAS is activated by cytosolic DNA derived from invading microbes^19^, damaged mitochondria^20^, ruptured nuclei and micronuclei^20–22^, or self-DNA from engulfed tumour cells^23^. Nuclear damage frequently arises as a consequence of mechanically induced deformation^24,25^. We found no evidence of an altered number of micronuclei (Supplementary Fig. 1b,c). Fluidification-via-flocking is, instead, accompanied by large fluctuations in cell density^12^ and area (Fig. 2e–g and Supplementary Video 4), which might result in increased nuclear deformation. We developed an automated image analysis pipeline to monitor nuclear shape changes over time to verify this conjecture. In control and RAB5-expressing MCF10A and MCF10.DCIS.com monolayers, tissue fluidification-via-flocking resulted in larger and faster deformations (Supplementary Video 5), which were measured by estimating the mean squared nuclear strain $MSS(\tau ) \equiv \left\langle {\left\langle {{\Delta}a_n^2(\tau |t)} \right\rangle _n} \right\rangle _t$ for different delay times *τ* and extracting the corresponding strain rate $\dot \gamma _0 \cong MSS(\tau )/\tau$ (Fig. 2h,i). In previous expressions, ${\Delta}a_n(\tau |t) \equiv \left[ {A_n(t + \tau ) - A_n(t)} \right]/\left\langle {A_n(t)} \right\rangle _t$, where *A*_*n*_(*t*) is the projected area of the *n*-th nucleus at time *t* and the symbols $\left\langle \cdot \right\rangle _n$ and $\left\langle \cdot \right\rangle _t$ indicate averages performed over all the segmented nuclei and over time, respectively.

**Extended Data Fig. 4:**
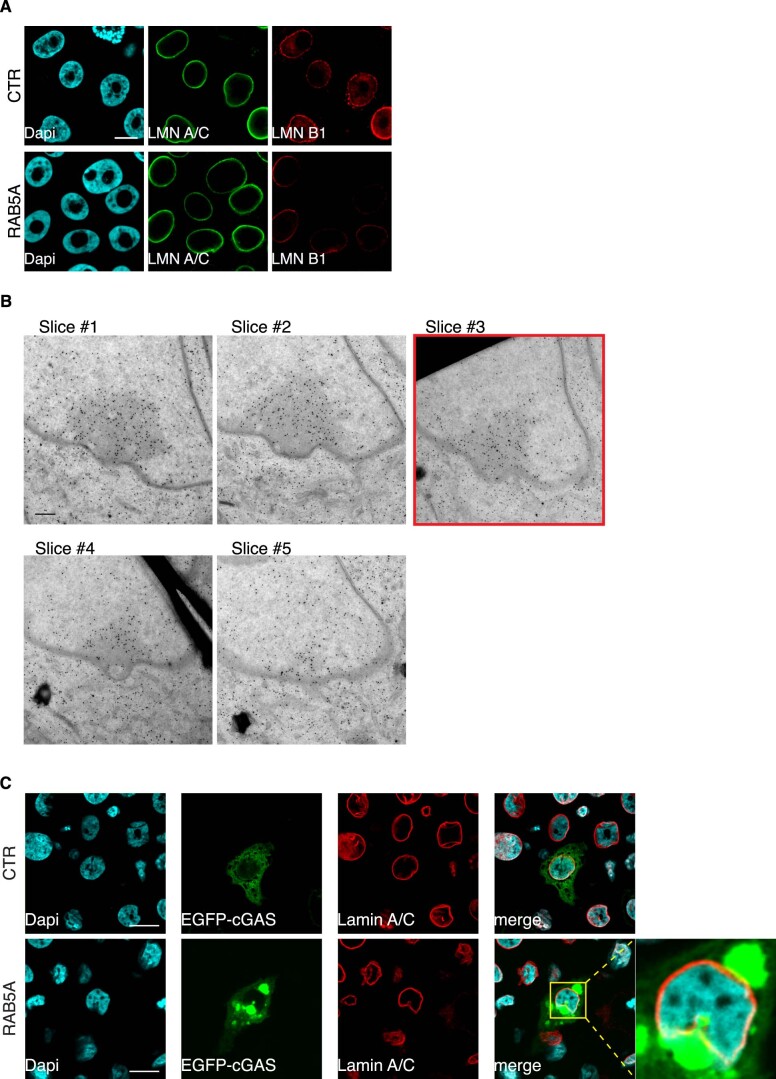
cGAS accumulation at nuclear envelope ruptures. **a**. Immunofluorescence images of control and RAB5A-monolayers (*n* = 3 independent experiments), stained with Dapi (Cyan), Lamin A/C (Green) and Lamin B1 (red). Scale bar 15 μm. **b**. Sequential Z axis sections of EGFP-cGAS-expressing RAB5A-MCF10.DCIS.com monolayers used for 3D tomographic reconstruction shown in Fig. 3f (lower right image). The image outlined in red correspond the one shown in Fig. 3f (upper right image). Scale Bar, 500 nm. **c**. Immunofluorescence images of control (CTR) and RAB5A-expressing (RAB5A) MCF10.DCIS.com monolayers (*n* = 4 independent experiments), transiently transfected with EGFP-cGAS and stained with DAPI and anti-Lamin A/C antibody to detect nuclei and nuclear lamina, respectively. Magnified images of the selected boxed area are shown. Scale bar 20 μm.

We also noticed that the expression of RAB5A resulted in a significant reduction of the mRNA levels of Lamin B1, but not of Lamin A/C, and of the protein levels measured by immunoblotting (Fig. 2j,k) and immunofluorescence (Fig. 2l and Extended Data Fig. 4a). The increased mechanical stress and reduced Lamin B1 levels might compromise nuclear integrity and result in more frequent ruptures of the nuclear envelope (NE), with the release of DNA into the cytoplasm that, in turn, can trigger cGAS activation. We verified this possibility in multiple ways.

**Fig. 3:**
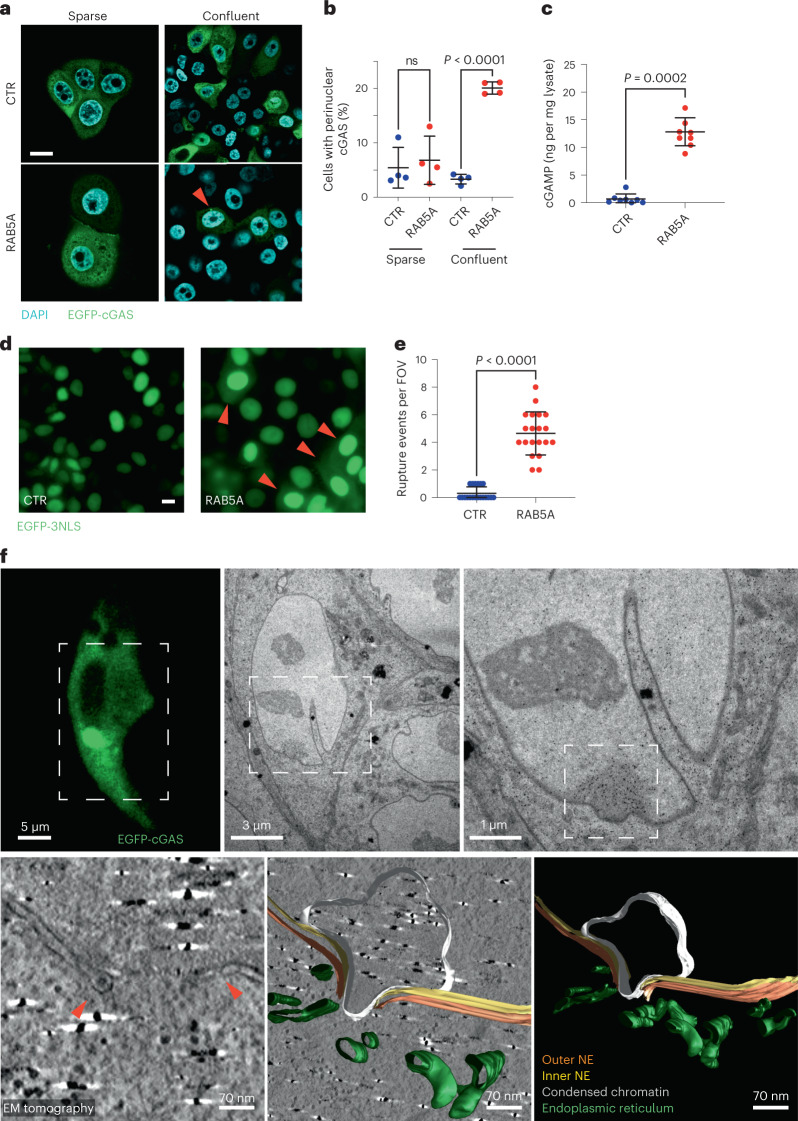
Nuclear envelope ruptures activate cGAS in fluidized monolayers. **a**, Immunofluorescence images of doxycycline-treated, EGFP-cGAS control (CTR) and RAB5A-expressing (RAB5A) MCF10.DCIS.com cells (*n* = 4 experiments) seeded either sparsely or as confluent monolayers. Scale bar, 10 μm. Red arrow point to EGFP-cGAS perinuclear foci **b**, Scatter plot of the percentage of MCF10.DCIS.com cells with perinuclear cGAS enrichment is expressed as the mean ± s.d. (>15 FOV per experimental condition in *n* = 4 independent experiments), unpaired two-tailed *t*-test with Welch’s correction. **c**, Quantification of cGAMP levels by ELISA from control (CTR) and RAB5A-expressing (RAB5A) MCF10.DCIS.com cell extracts. Data are the cGAMP amounts (ng) per mg of total cell extract expressed as the mean ± s.d. (*n* = 8 experiments), two-tailed Mann–Whitney non-parametric test. **d**, Snapshot of time-lapse (Supplementary Video 6) EGFP-3NLS-expressing control (CTR) and RAB5A MCF10.DCIS.com monolayers (*n* = 2 independent experiments), displaying events of NE rupture and EGFP-3NLS leakage (red arrowheads). Scale bar, 10 μm. **e**, Scatter plot of the number of nuclear envelope rupture events per FOV reported as mean ± s.d. (10 FOV per experimental conditions in *n* = 2 independent experiments), two-tailed Mann–Whitney non-parametric test. **f**, CLEM analysis of cGAS perinuclear foci. RAB5A-expressing MCF10.DCIS.com monolayers transfected with EGFP-cGAS were plated on MaTek dishes with grids. Cells identified on grids by confocal microscopy were processed for electron microscopy and *z*-axis serial sections were stained with gold-labelled anti-GFP antibody to detect EGFP-cGAS (right). Dashed boxes indicate regions that were progressively magnified in EM. The bottom images show a 3D tomographic reconstruction (left) and 3D models of an NE rupture site (right panels). Arrowheads indicate the site of NE rupture (*n* = 2 independent experiments). Source data

Firstly, we expressed cGAS-fused to EGFP (EGFP-cGAS) and monitored its localization and distribution. In dense, kinetically arrested MCF10.DCIS.com monolayers and in sparsely seeded cells EGFP-cGAS displayed a primarily cytoplasmic diffuse staining, as expected^26,27^ (Fig. 3a). Conversely, a focalized perinuclearly restricted localization was seen after RAB5A induction in flocking-fluid monolayers, but not in sparsely seeded cells (Fig. 3a,b), similarly to what occurs after nuclear envelope ruptures^28^.

Secondly, RAB5A-flocking fluid cells exhibited a significant increase in cGAMP levels, the product of cGAS enzymatic activity^29,30^ (Fig. 3c).

Thirdly, we monitored nuclear envelope ruptures through real-time analysis of the dynamics of the 3NLS-EGFP sensor. 3NLS-EGFP displayed a nuclear restricted expression in control cells, but a cytoplasmic distribution in fluidized RAB5A-expressing monolayers, indicative of NE ruptures (Fig. 3d,e and Supplementary Video 6).

Finally, we performed correlative-light electron microscopy (CLEM) tomography and immune EM to directly visualize the presence of NE ruptures. In RAB5A, but not control cells, EGFP-cGAS accumulated at sites of condensed chromatin, immediately adjacent to the region where both the inner and outer NE membranes were ruptured (Fig. 3f and Extended Data Fig. 4b). Immunofluorescent staining of Lamin A/C also revealed that nuclei in RAB5A-fluidized monolayers undergo large deformation and possibly ruptures as indicated by the accumulation of cGAS around distorted nuclei and at the apex of nuclear invagination (Extended Data Fig. 4c).

## Tissue fluidification triggers mechano-protective responses

The large fluctuations in tissue density, cell area and nuclear shape suggest that RAB5A-fluidized epithelial collectives are subjected to persistent and chronic mechanical strain and stress. These stresses can compromise tissue integrity^31^ and cause nuclear rupture and DNA damage^24,25^. Both individual cells and epithelial sheets, however, can adapt to acute stress by mounting a nuclear mechano-protective response that preserves them from widespread genomic damages^32^. These responses include increases nuclear rigidity and size, elevation in chromatin compaction^32,33^, and the remodelling of peri-nuclear cytoskeletal actin with the formation of nuclear actin rings^28,34^. We hypothesized that endocytic unjamming-via-flocking exerts prolonged mechanical stress in epithelial ensembles that react by mounting a mechano-protective strategy, which, eventually, fails resulting in DNA damage. We set out to investigate this possibility.

**Fig. 4:**
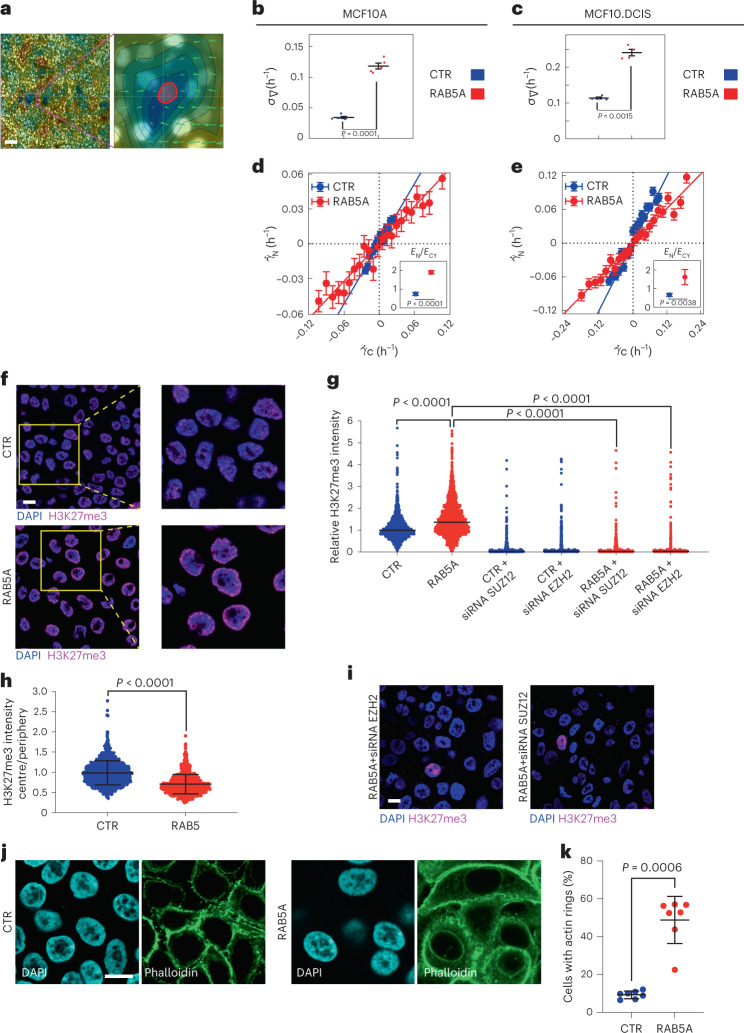
Tissue fluidification induces nuclear stiffness, heterochromatin reorganization and actin remodelling. **a**, Left: map of the divergence of the velocity field from PIV in RAB5A-MCF10A monolayer. Cold (warm) colours indicate negative (positive) values of divergence. Right: magnified view of a smaller portion (70 × 70 μm) centred on a segmented nucleus (red outline). The velocity field (green arrows) converges to the central cell, corresponding to a local negative value of divergence and compressive deformation. Scale bar, 70 μm. **b**,**c**, Root mean square value *σ*_∇_ of the divergence of the velocity field from PIV MCF10A (**b**) and MCF10.DCIS.com (**c**) monolayers. Different points correspond to different FOVs, each one corresponding to ~1.2 × 10^4^ and ~8 × 10^3^ cells for MCF10A and MCF10.DCIS.com samples respectively. Black lines are the averages ± s.d. two-tailed *t*-test. **d**,**e**, Nuclear strain rate $\dot \gamma _N$ as a function of the corresponding cell strain rate $\dot \gamma _C$ for MCF10A (**d**) and MCF10.DCIS.com (**e**) monolayers. $\dot \gamma _N$ is obtained from nuclear segmentation, while $\dot \gamma _C$ is estimated from the divergence of the velocity field. Data are grouped into evenly spaced bins along the horizontal axis. Symbols and error bars are the mean and standard deviation of the $\dot \gamma _N$-values in each bin, respectively. Straight lines are best fitting curves with a linear model $\dot \gamma _N = s\dot \gamma _C$. Insets: the ratio between the effective elastic moduli *E*_N_/*E*_CY_ of the nucleus and the cytoplasm reported as mean ± s.d. obtained as the slope of best fitting line to the data in the main panel (*n* = 10 randomly populated subsets of cells), two-tailed *t*-test. **f**, Immunofluorescence images of control (CTR) and RAB5A MCF10.DCIS.com monolayers (*n* = 3 experiments), stained with DAPI and anti-H3K27me3-antibody. Magnified images are shown. Scale bar, 10 μm. **g**, Relative H3K27me3 intensity of control (CTR) and RAB5A MCF10.DCIS.com monolayers silenced or not for EZH2 or SUZ12. Each dot represents a cell, and the median is indicated (>1,400 cells per experimental condition for CTR and RAB5A, >400 cells per experimental condition in *n* = 3 independent experiments for siRNA-treated conditions), Kruskal–Wallis/Dunn’s test. **h**, Ratio of H3K27me3 intensity of the nuclear central region over the periphery in control-(CTR) and RAB5A-(RAB5A)-MCF10.DCIS.com cells. Each dot is a cell and the mean ± s.d. is indicated. (>1,000 cells per experimental conditions in *n* = 3 experiments), two-tailed Mann–Whitney non-parametric test. **i**, Immunofluorescence images of control (CTR) and RAB5A MCF10.DCIS.com monolayers (*n* = 3 experiments), silenced for *EZH2* or *SUZ12* and stained with DAPI and anti-H3K27me3-antibody. Scale bar, 10 μm. **j**, Immunofluorescence images of control (CTR) and RAB5A MCF10.DCIS.com monolayers (*n* = 2 experiments), stained with phalloidin to detect F-actin. Scale bar, 20 μm. **k**, Percentage of cells with actin rings per FOV expressed as mean ± s.d (dots represent seven FOVs in *n* = 2 independent experiments), two-tailed Mann–Whitney non-parametric test. Source data

Firstly, we investigated how nuclei respond to motility-induced fluctuation in the local cell density *ρ* in jammed and fluid monolayers^12,35^. We considered how the instantaneous nuclear strain rate $\dot \gamma _N = \frac{1}{A}\frac{{\partial A}}{{\partial t}}$ of each cell nucleus depends on the local monolayer strain rate $\dot \gamma _C = - \frac{1}{\rho }\frac{{\partial \rho }}{{\partial t}}$, which we estimated as the divergence of the velocity field from PIV analysis (Fig. 4a–c). In all cases, a significant correlation is found between $\dot \gamma _N$ and $\dot \gamma _C$, indicating that the nucleus systematically deforms in response to compressive and tensile strains imposed on the cell by the relative motion of its neighbours. RAB5A-fluidized monolayers undergo larger density fluctuations than control-jammed monolayers (Fig. 4b,c). Furthermore, the nearly linear relation between nuclear and cell strain rates is characterized by markedly different slopes in the two cases: in response to the same variation in the cell density, nuclei of RAB5A-fluidized monolayers deform significantly less (Fig. 4d,e), that is, they are stiffer. Using a simple mechanical model, described in ref. ^36^, the slope of the $\dot \gamma _N$ versus $\dot \gamma _C$ curve can be used to estimate the ratio *E*_N_/*E*_CY_ between the effective elastic moduli *E*_N_ and *E*_CY_ characterizing the mechanical response to in-plane compressive/tensile stresses of the two main cellular compartments, nucleus and cytoplasm, respectively. We found that the ratio *E*_N_/*E*_CY_ is about twice as large in RAB5A-fluidized monolayers, indicating a significant increase in nuclear stiffness compared with controls (inset of Fig. 4d, e).

**Extended Data Fig. 5:**
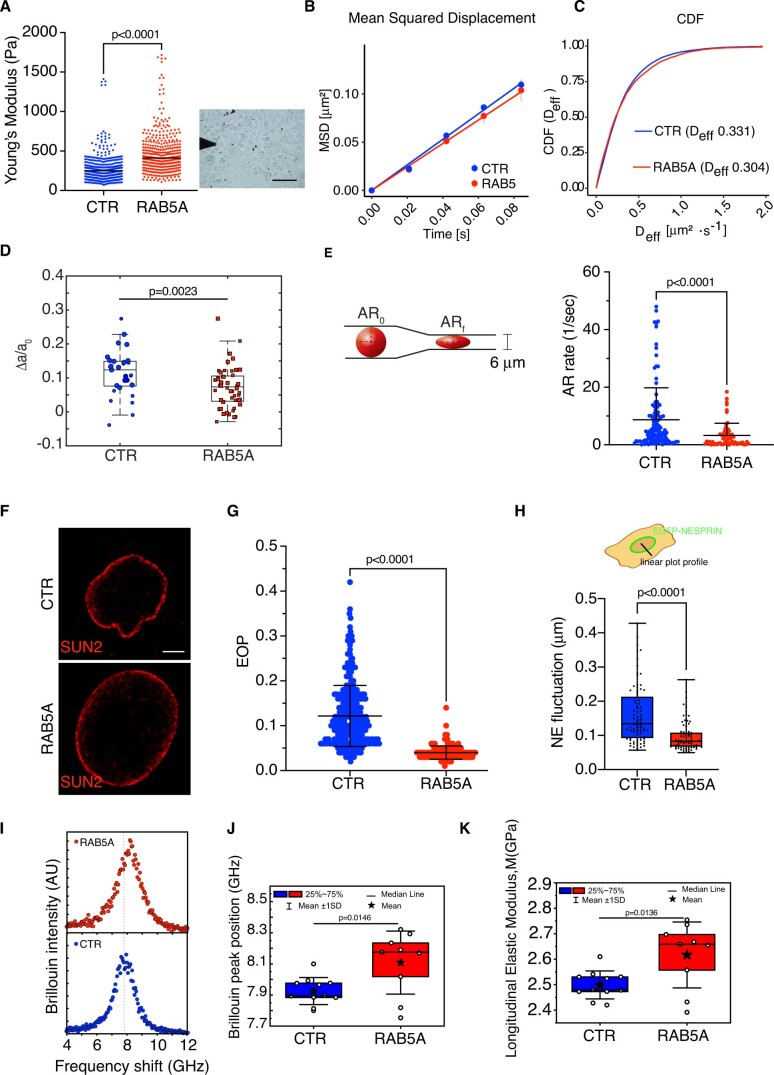
Mechanical characterization of nuclei in control and fluidized tissues. **a**. Nuclear elasticity (Young’s modulus) using atomic force microscopy (AFM)-based force indentation of the nuclear surface through the cell cortex of control and RAB5A-MCF10.DCIS.com monolayers seeded at jamming density. Data are the mean ± s.e.m. (100 cells/condition in *n* = 3 independent experiments), two-tailed Mann–Whitney non-parametric test. Scale bar, 200 µm. **b, c**. Quantification of cytoplasmic rheology using genetically encoded multimeric nanoparticle (GEM)^37^. **b**) Time- and ensemble- averaged Mean Square Displacement (MSD) plots for control (blue line) and RAB5A (red line) in MCF10.DCIS.com monolayers seeded at jamming density. **c**) Cumulative distribution function showing the coefficient of diffusion, CDF, (D_eff_) data of for control (blue line) and RAB5A (red line) MCF10.DCIS.com monolayers seeded at jamming density. **d**. Nuclear deformation of control and RAB5A monolayers subjected to a 22% biaxial stretch. Control and RAB5A-MCF10.DCIS.com monolayers were seeded onto elastic PDMS membranes in a biaxial stretching device^63^. Thenuclear area strain, Δa was measured in various, randomly picked fields of view. Bottom, middle and top lines of boxes show the 25th, 50th and 75th percentiles, respectively, and whiskers show 1.5 times the interquartile range from hinges (>29 FOV/experimental condition in *n* = 2 independent experiments, indicated by different size symbols, respectively), two tailed Mann–Whitney non-parametric test. **e**. The rates of aspect ratio (A/R) changes of mCherry-H2B control (CTR) and RAB5A-MCF10.DCIS.com cells that are passively going through a constricted 6 micrometre-wide channel schematically depicted on the left (Supplementary Video 7), were calculated for several cells in the time interval needed to pass from an undeformed (A/R_0_, at t_0_), just before entering into the constriction, to a completely squeezed configuration (A/R_f_, at t_f_). The rates of nuclear AR variation were extrapolated through a robust fit in R using the ‘robustbase’ package http://robustbase.r-forge.r-project.org/, where the rate of AR changes as function of time is the slope of the fitted curve. Scatter plot data of mean ± S.D. (114 cells/control and 61 cells/RAB5A of *n* = 3 independent experiments) of the rate of A/R changes, two-tailed Mann–Whitney non-parametric test. **f**. Images of the SUN2-stained NE of control and RAB5A-MCF10.DCIS.com cells (*n* = 3 experiments), seeded at jamming density. Scale bar 2 µm. **g**. Scatter plot of EOP quantifications expressed as mean ± s.d. (> 181 cells/condition in *n* = 3 independent experiments), two-tailed Mann–Whitney non-parametric test. **h**. Quantification of NE fluctuations in EGFP-Nesprin-expressing, control and RAB5A-MCF10.DCIS.com cells. Nuclei of live cells transfected with EGFP-Nesprin were recorded at 250 msec per frame for 5 min (Supplementary Video 8). NE fluctuations were obtained from the standard deviation of the NE from its mean position (see Methods and in the inset scheme). The box extends from the 25th to 75th percentile, the whiskers show minimum and maximum values, and the line within the box represents the median *(n* = 60 position along the NE/condition in >10 cells/ condition), two-tailed Mann–Whitney non-parametric test. **i–k**. Nuclear mechanics of live cells obtained by Brillouin micro-spectroscopy. **i**) Representative Brillouin spectra (anti-Stokes peaks) of nuclei of RAB5A (upper panel) and control cells (lower panel). **j**) Mean and median of Brillouin shifts (black line) measured in RAB5A (n = 9) and control cells (n = 11). The box extends from the 25th to 75th percentile, the whiskers show the mean ± s.d., and the line within the box represents the median, unpaired one-tailed t-test with Welch’s correction. **k**) From Brillouin shifts, we derived the apparent longitudinal elastic modulus. The box extends from the 25th to 75th percentile, the whiskers show the mean ± s.d., and the line within the box represents the median, unpaired one-tailed t-test with Welch’s correction. P values are indicated in each graph. Source data

We corroborated this finding by probing the mechanical properties of RAB5A-fluidized nuclei and monolayers through several orthogonal approaches. We quantify nuclear elasticity using atomic force microscopy (AFM)-based force indentation of the nuclear surface through the cell cortex. Nuclear force indentation showed an approximately twofold increase in the stiffness in RAB5A-fluidized monolayers as compared with control (Extended Data Fig. 5a). We also probed the rheological properties of the cytoplasm, which may contribute to the difference in Young’s modulus obtained by AFM indentation. We found that the mean square displacement and coefficient of diffusion of genetically encoded, cytoplasmic fluorescence particles (GEM)^37^ were not significantly different in control-arrested versus flocking-fluid RAB5A monolayers (Extended Data Fig. 5b,c). Thus, the increased stiffness of RAB5-fluidized cells is likely the result of the increased nuclei rigidity.

**Extended Data Fig. 6:**
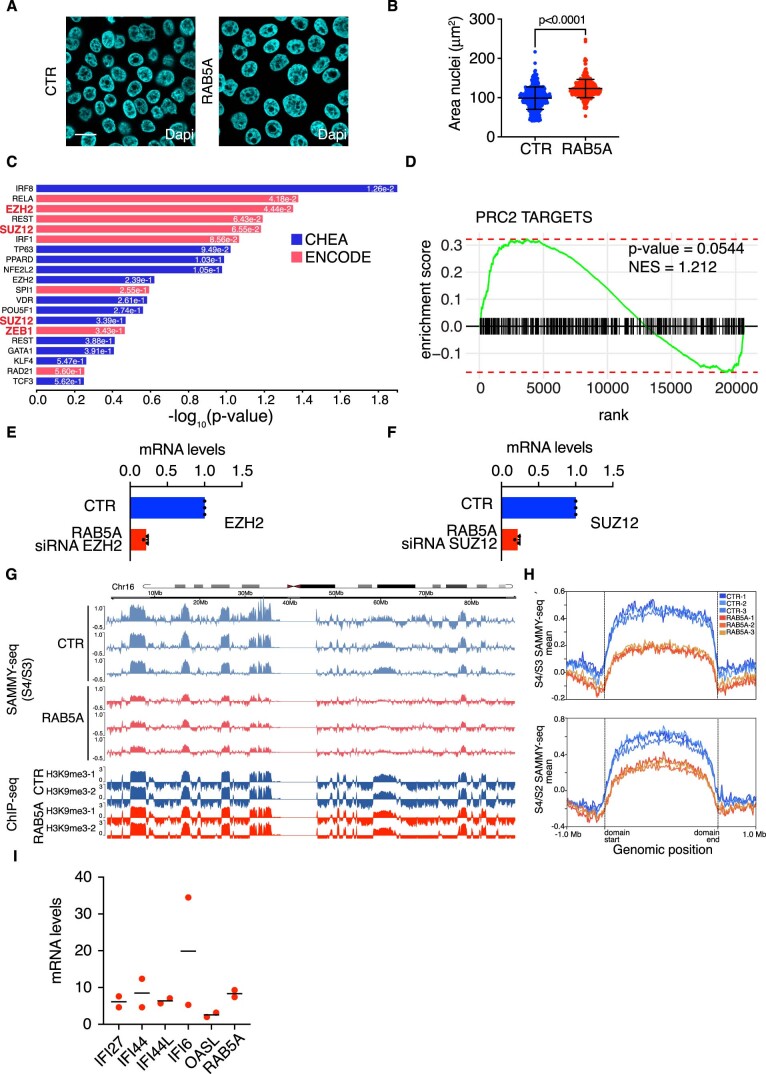
Fluidized MCF10.DCIS.com monolayers display an increase nuclear size and enrichment in PRC2-targets. **a**. Immunofluorescence images of nuclei in control (CTR) and RAB5A-(RAB5A)-MCF10.DCIS.com monolayers (*n* = 3 experiments). Scale bar 20 μm. **b**. Scatter Plot of the nuclear area per field of view is the mean ± s.d. (> 100 FOV/experimental conditions in *n* = 3 independent experiments), two-tailed Mann–Whitney non-parametric test. **c**. Transcription factor enrichment analysis of DEG in MCF10.DCIS.com over control monolayers and entries of the ChEA and ENCODE databases. In the Bars are one-sided P-value (combination of Fisher’s exact test and deviation from expected rank for random input gene-set). Bars are coloured according to the transcription factor/target-gene interactions from ChIP-seq/chip experiments database where they have been found enriched. **d**. GSEA Enrichment plot of the PRC2 targets gene set in the CGP collection of the GSEA Molecular Signatures Database using DEG in RAB5A-MCF10.DCIS.com monolayers. The green curve is the ES (enrichment score) curve, which is the running sum of the weighted enrichment score obtained from GSEA software, while the normalized enrichment score (NES) and the corresponding one-sided test is shown. **e, f**. Box plot of the mRNA expression levels of EZH2 and SUZ12 in RAB5A-MCF10.DCIS.com monolayer over control cells silenced with the indicated oligos. Data are the mean ± s.d. (*n* = 3 independent experiments). Values were normalized to the controls of each experiment. **g**. Visual representation of genomic tracks for ChIP-seq signal enrichment and SAMMY-seq fractions comparison for a representative chromosome (chr 16). For each position along the chromosome (x axis) the enrichment signal (y axis) is the normalized log2 ratio of ChIP over input control samples sequencing reads for ChIP-seq and the normalized log2 ratio of S4 over S3 sequencing (S4/S3) for SAMMY-seq. The data shown in the genomic tracks (from top to bottom) are: three SAMMY-seq replicates on CTR and RAB5A samples; two H3K9me3 ChIP-seq replicates (performed with two alternative antibodies - see methods) on CTR and RAB5A. **h**. Average SAMMY-seq enrichment signal over heterochromatic regions (metaprofile). SAMMY-seq experiment profiles computed on CTR and on RAB5A-expressing samples are reported (3 replicates for each condition depicted with lines of different colors - see colour legend). The heterochromatic regions were selected based on H3K9me3 ChIP-seq (with ab-176916-Abcam antibody) enriched domains (n = 78 domains). The x-axis reports the relative position with respect to the start and end borders of the considered domains (domain bodies rescaled) in addition to 1Mb flanking regions with absolute (unscaled) coordinates. The y-axis reports the average SAMMY-seq enrichment across all the considered domains. Similar results are obtained by considering either the S4/S2 or the S4/S3 SAMMY-seq fractions comparisons (log2 ratios) to highlight the location of the less accessible and soluble chromatin fraction. **i**. mRNA expression levels of IFI27, IFI44, IFI44L, IFI6, OASL and RAB5A in RAB5A- MCF10.DCIS.com cells over control cells injected into mammary fat pads of mice. After one week, mice were fed with doxycycline to induce RAB5A expression and the primary tumours were isolated 4 weeks after doxycycline treatment. Data are the mean (*n* = 2 independent experiments). Values were normalized to the controls of each experiment. P values are indicated in each graph. Source data

Next, we subjected control and RAB5A monolayers plated on a deformable polydimethylsiloxane (PDMS) membrane to a predefined 22% biaxial stretch and measured the ensuing cell and nuclear deformations. The cell nuclei of flocking fluid monolayers deformed about twofold less than control cells (Extended Data Fig. 5d). Exploiting the same model used to interpret motility-induced deformations, the results of this stretching experiment can be expressed in terms of the ratio *E*_N_/*E*_CY_ between the effective elastic moduli associated to the nucleus and the cytoplasm (Methods). This ratio was found to be about twice as large in RAB5A monolayers (7.1) compared with controls, (4.5) (Extended Data Fig. 5d). Similarly, the nuclear deformation, obtained by determining the rate of aspect ratio changes, of RAB5A-expressing cells that are forced into a restricted 6 μm-wide channel was two-time less than in control cells (Extended Data Fig. 6e and Supplementary Video 7), consistent with the increased nuclear stiffness.

The increased rigidity of RAB5A nuclei might impact the nuclear shape at a steady state. Thus, we measured nuclear shape variations in control and fluidized monolayers by determining the dimensionless parameter excess of the perimeter of the projected nuclear shape (EOP) through immunofluorescent analysis of SUN2, an inner nuclear membrane protein^38^. EOP values of a highly folded, presumably floppy, and soft object are expected to be close to 1, whereas EOP of a rigid object tends to be close to 0. The EOP values of RAB5A fluidized monolayers were significantly closer to 0 with respect to control-jammed ones (Extended Data Fig. 5f,g). We also estimated the nuclear envelope shape fluctuations assuming that rigid nuclei should display reduced fluctuation with respect to softer or floppy ones. We monitored nuclei in live cells expressing a mini-EGFP-Nesprin1, which encompasses the Calponin actin-binding domain and the c-terminus of the NE protein Nesprin1 (ref. ^39^), at high frame rates. The amplitude of NE fluctuations was next calculated by measuring the standard deviation of the NE from its mean position. The NE fluctuations were significantly reduced in RAB5A fluidized cells (Extended Data Fig. 5h and Supplementary Video 8).

Finally, we employed Brillouin microscopy to probe nuclear mechanical properties. We detected a clear Brillouin frequency shift that corresponds to a significant increase in the longitudinal elastic modulus, *M*, indicative of increased nuclear stiffness, of RAB5A-expressing cell nuclei as compared with control (Extended Data Fig. 5i–k).

We also showed that nuclear-projected areas are nearly 25% larger in RAB5A-fluidized monolayers as compared with control ones (Extended Data Fig. 6a,b). Nuclei of RAB5A-expressing cells appeared enlarged, and their nuclear envelope might be under tension, in keeping with the reduced EOP of the nucleus and NE fluctuations.

To probe the heterochromatin state, we initially examined the nuclear levels of H3K27me3. RAB5A-fluidized DCIS cells display a small but significant increase in H3K27m3-heterochromatin marks (Fig. 4f,g), which were enriched at the nuclear periphery (Fig. 4h). Analysis of the top 100 upregulated genes revealed among the top transcription factors, EZH2, a histone H3 lysine 27 *N*-methyltransferase, and SUZ12, a key component of the polycomb repressor complex-2 (PRC2) (Extended Data Fig. 6c). These enzymes deposit H3K27m3 in response to nuclear mechanical stress^40^. Additionally, a pre-ranked GSEA showed enrichment in genes that can be targeted by PRC2 (Extended Data Fig. 6d). Consistently, silencing of *EZH2* or *SUZ12* abrogated the increase in H3K27me3-heterochromatin marks (Fig. 4g,i, and Extended Data Fig. 6e,f).

The chronic mechanical stress together with Lamin B1 reduction in RAB5A-fluidized monolayers might also elicit genome-wide structural alterations in constitutive H3K9me3 normally associated with the lamina, as a mechanism to dissipate forces^28^. We employed SAMMY-Seq and H3K9me3 ChIP-seq to verify this possibility. SAMMY-Seq is a high-throughput sequencing-based method for genome-wide characterization of chromatin accessibility, which can detect architectural rearrangements of lamina-associated heterochromatin domains^41^. RAB5A-fluidized monolayers displayed no changes in the H3K9me3-genome-wide ChIP-seq profile (Extended Data Fig. 6g), but a consistent reduction in the SAMMY-seq signal for heterochromatin regions (Extended Data Fig. 6h).

Next, we found that tissue fluidification is also accompanied by cell shape changes and perinuclear remodelling of the actin cytoskeleton. Doxycycline induction of RAB5A resulted in perturbations of the shape of cells (Fig. 4j), increased cytoplasmic polymerized actin and the formation of perinuclear actin rings (Fig. 4j,k).

**Fig. 5:**
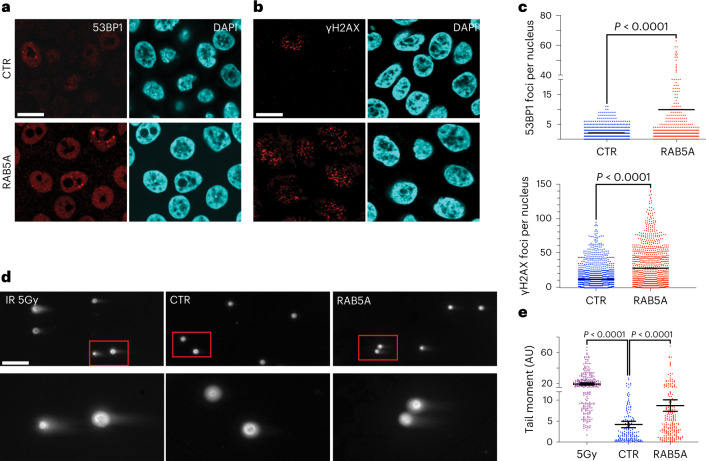
Endocytic-dependent tissue fluidification results in DNA damage. **a**,**b**, Images of control and RAB5A-MCF10.DCIS.com monolayers (*n* = 3 experiments), stained with the indicated antibodies. Scale bar, 20 μm. **c**, The scatter plot shows the mean of 53BP1 foci per nuclei or γH2AX foci per nuclei (>150 cells in *n* = 3 experiments), two-tailed Mann–Whitney non-parametric test. **d**, Representative images of neutral comet assay in MCF10.DCIS.com monolayer cells expressing RAB5A. Control cells irradiated (IR 5 Gy) or not (CTR) are also shown. Scale bar, 100 μm. **e**, Quantification of DNA damage by tail moment analysis. Horizontal bars indicate the means and s.d. of from *n* = 2 independent experiments; >100 cells per sample were scored, one-way ANOVA. Source data

RAB5A-fluidized monolayers mount a complex mechano-protective response, which leads to decreased nuclear pliability and softness, suggesting the possibility that these monolayers are less capable of dissipating mechanical energy to prevent DNA damage^28^. Consistently, RAB5A-flocking monolayers display elevated DNA damage, as evidenced by the increase in 53BP1 and γH2AX foci (Fig. 5a–c), and in the tail moment determined by neutral DNA comet assays^42^ (Fig. 5d,e).

## RAB5A induces cGAS activation in invasive carcinoma

**Fig. 6:**
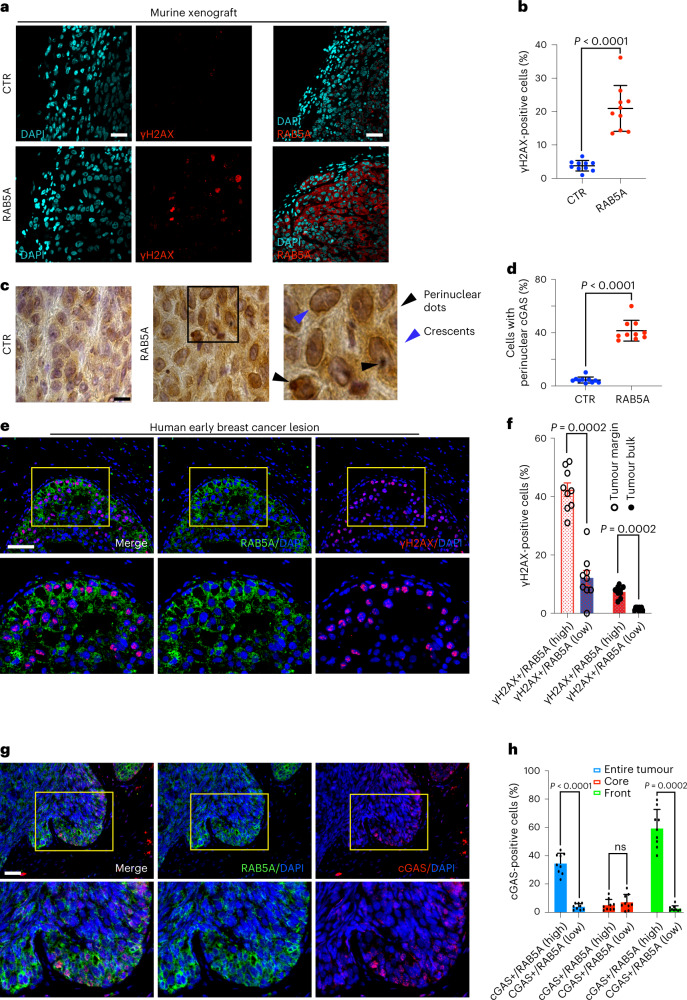
Increased RAB5A-expression, cGAS perinuclear accumulation and DNA damage in human invasive ductal carcinoma. **a**, Immunofluorescence images of control (CTR) and RAB5A MCF10.DCIS.com cells injected into mammary fat pads of immunocompromised mice. After one week, mice were fed doxycycline to induce RAB5A expression. Four weeks after doxycycline treatment, primary tumours were co-stained with DAPI and γH2AX, or DAPI and RAB5A. Scale bar, 100 μm. **b**, Percentage of γH2AX-positive cells per FOV. Data are mean ± s.d. (*n* = 2 experiments, with five mice per experiment), unpaired two-tailed *t*-test with Welch’s correction. **c**, Immunohistochemical analysis of cGAS in control (CTR) and RAB5A MCF10.DCIS.com cells injected into mammary fat pads of immunocompromised mice and treated as in **a**. Primary tumours were stained with an anti-cGAS antibody. Right panel: a higher magnification of the boxed region to highlight cGAS accumulation in perinuclear dots (black arrowheads) or crescent-like distribution (blue arrowhead). Scale bar 80 μm. **d**, The percentage of cells with perinuclear cGAS enrichment is expressed as the mean ± s.d. (each dot represents an FOV out of >5 FOVs per experimental condition in *n* = 2 independent experiments), two-tailed Mann–Whitney non-parametric test. **e**, Multiplex immunohistochemistry/Immunofluorescence (mIHC/IF) of RAB5A, γH2AX and DAPI in human DCIS. Magnified images from the selected yellow boxes are shown. Scale bar, 150 μm. **f**, The percentage of γH2AX-positive nuclei in cells that express high (>2 on a scale from 0,1,2,3) or low (<2 on a scale from 0,1,2,3) levels of RAB5A in the tumour bulk or margin (see Methods for details and Supplementary Video 13). The areas of *n* = 9 independent DCIS were analysed with more than 200 nuclei per area, two-tailed Mann–Whitney non-parametric test. **g**, Multiplex immunohistochemistry/immunofluorescence (mIHC/IF) of RAB5A, cGAS, and Dapi in human DCIS. Magnified images from the selected yellow boxes are shown. Scale bar, 300 μm. **h**, The percentage of cGAS-positive cells that express high (>2 on a scale from 0,1,2,3) or low (<2 on a scale from 0,1,2,3) levels of RAB5A in the tumour bulk, or the margin or the whole tumour (Methods). The areas of *n* = 9 independent DCIS were analysed with more than 200 nuclei per area, two-tailed Mann–Whitney non-parametric test and unpaired two-tailed *t*-test with Welch’s correction. Source data

**Extended Data Fig. 7:**
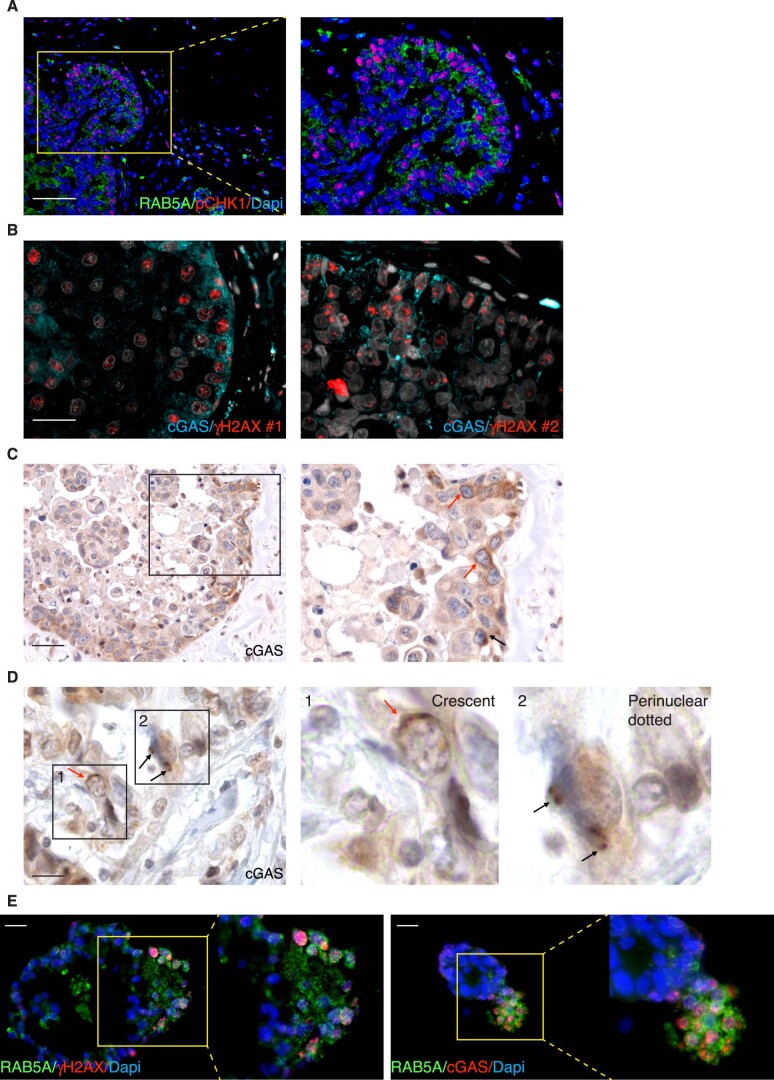
IHC characterization of RAB5A, pCHK1 and cGAS in human DCIS and breast cancer organoids. **a**. Representative (1 out of more than 20 tested) multiplex immunohistochemistry/immunofluorescence (mIHC/IF) of RAB5A, pCHK1 and DAPI in human Ductal breast Carcinoma *in Situ* (DCIS). A magnified image from the selected yellow boxes is shown. Scale bar 250 μm. **b**. Representative (1 out of more than 20 tested) multiplex OPAL immunohistochemistry/immunofluorescence (mIHC/IF) of cGAS and γH2AX in 2 distinct human DCIS. Scale bar 100 μm. **c**. Representative immunohistochemistry analysis of cGAS in human DCIS with invasive buds in n = 5 distinct human DCIS. A magnified image from the selected boxed area is shown. Red Arrow indicates the crescent-like, perinuclear accumulation of cGAS signal. Scale bar 80 μm. **d**. Representative immunohistochemistry analysis of cGAS in locally invasive human DCIS (*n* = 9 distinct human DCIS). Magnified images from the selected boxed area are shown. Red arrow indicates the crescent-like, perinuclear accumulation of cGAS signal, black arrows indicate the perinuclear dotted distribution of cGAS. Scale bar 40 μm. **e**. Representative multiplex immunohistochemistry/Immunofluorescence (mIHC/IF) of RAB5A, γH2AX, cGAS and DAPI in *n* *=* *5* patient-derived breast cancer organoids. Magnified images from the selected yellow boxes are shown. Scale bar 150 μm.

**Extended Data Fig. 8:**
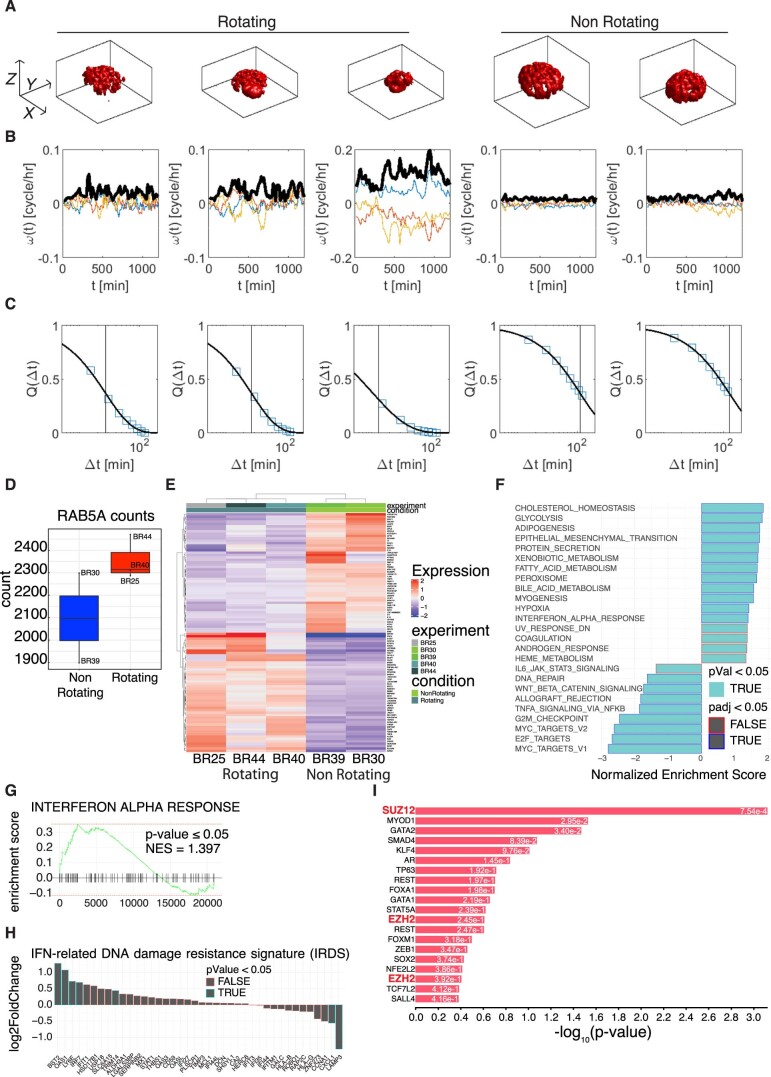
Human breast cancer organoids that persistently rotate display elevated RAB5A, and enrichment in ISG and PRC2-dependent genes. **a**. 3D rendering of 5 living breast cancer organoids. The size of each box along the x- and y-directions corresponds to 200 μm. **b**. Thin blue, orange and yellow curves: temporal evolution of the x, y and z components of the angular velocity associated with the rotation of the organoid depicted in the box above each panel, respectively. Thick black curves represent the angular speed, *that is*, the modulus of the angular velocity. Organoids are categorized as ‘rotating’ if their average angular speed is larger than 0.03 cycles/h. **c**. Symbols: overlap parameter *Q*(*τ*) obtained from 3D-DVA analysis, capturing the internal rearrangement dynamics of the organoid depicted in the box above each panel. Continuous thin lines are best fitting curves to the data with an exponential model $Q\left( \tau \right) = e^{ - \tau /\tau ^ \ast }$. Vertical thick lines are drawn in correspondence with the characteristic relaxation time τ^*^ obtained from the fitting model. **d**. Expression levels of RAB5A isoform in *n* = 3 patients-derived rotating (red) and *n* = 2 patients-derived non-rotating (blue) organoids. Bottom, middle and top lines of boxes show the 25th, 50th and 75th percentiles, respectively, and whiskers show 1.5 times the interquartile range from hinges. **e**. Heatmap showing the top 100 deregulated genes (top 50 up and top 50 down) in each of the 3 replicates of the rotating and non-rotating organoids. **f**. GSEA of DEG in rotating *versus* non-rotating organoids. Moderated t-statistic was used to rank the genes. Reported are significantly enriched pathways (one-sided P-value < 0.05; number of random genes with the same or more extreme ES value divided by the total number of generated gene sets) with the colour of the outline of the bar corresponding to the BH-adjusted P-value. **g**. GSEA Enrichment plot of DEG in rotating *versus* non-rotating organoids. The green curve corresponds to the ES (enrichment score) curve, which is the running sum of the weighted enrichment score obtained from GSEA software, while the normalized enrichment score (NES) and the corresponding one-sided P-value are reported within the graph. **h**. Quantitative changes in the expression of the INF-related DNA damage resistance signature genes in rotating *versus* non-rotating organoids. The log2Fold Change is plotted on the x-axis and the significance (Wald test p value two-sided) is defined by the colour code of the outline. **i**. Transcription factor enrichment analysis for overlap between the input set of differentially expressed genes in rotating *versus* non-rotating organoids and entries of the ChEA and ENCODE databases In the bars is the one-sided P-value (combination of Fisher’s exact test and deviation from expected rank for random input gene-set). Source data

Next, we studied whether the nuclear mechano-perturbations leading to cGAS activation and DNA damage observed in vitro are also relevant in pathological tissues. Control and RAB5A-MCF10.DCIS.com cells were injected into the mammary fat pads of immunocompromised animals to model DCIS. In these tumours, RAB5A induction increased CytoDR, as expected (Extended Data Fig. 5i), and was associated with an elevated number of γH2AX-positive cells (Fig. 6a,b), and increased levels of cGAS, which display a perinuclear dotted or crescent-like appearance (Fig. 6c,d). This pattern is similar to the one seen in human DCIS with local infiltrative areas (Extended Data Fig. 8c,d), likely reflecting its activation by cytoplasmic DNA. In human tumours, RAB5A displayed a graded increase in expression right at the margin of locally invasive foci (Fig. 6e–g and Extended Data Fig. 7a). Notably, these marginal cells, also displayed increased γH2AX, and phosphorylated checkpoint kinase 1 (pCHK1; Fig. 6e,f and Extended Data Fig. 7a), a marker of persistent DNA damage^43^, and more relevantly of cGAS (Fig. 6g,h and Extended Data Fig. 7b–d). Similar graded expression of RAB5A associated with elevated γH2AX and cGAS was also detected in patient-derived breast cancer organoids (Extended Data Fig. 7e).

Quantitative analysis of nuclear motion in living breast cancer organoids revealed that some of them displayed persistent rotational motion and all the key features of a flocking fluid (Supplementary Video 9). By combining 3D image registration with a differential analysis of the residual intensity fluctuations^13^, we decoupled the rigid body contribution associated with the global rotation of the organoid from the internal rearrangement dynamics, which is captured by the overlap parameter *Q*(*τ*). Persistently rotating organoids also displayed markedly faster internal dynamics, with a relaxation time τ^*^ at least two times shorter than the static ones (Extended Data Fig. 8a–c and Supplementary Video 10). Next, we subjected the two static and the three rotating organoids to RNA-seq analysis. Despite the limited number of samples, we found that rotating fluid organoids display a significantly elevated expression of RAB5A (Extended Data Fig. 8d), enrichment in several genes belonging to the interferon-alpha stimulated and Interferon-related DNA damage resistance signature (Extended Data Fig. 8e–h). Whereas the analysis of the top deregulated genes pointed to PRC2 complex components, *SUZ12* and *EZH2*, as a key altered transcription factor in rotating organoids (Extended Data Fig. 8i).

## Tissue fluidification promotes EMT and chemoresistance

Chronic stimulation of cGAS–STING signalling has been shown to exert either immuno-protective or pro-tumorigenic effects. For example, by establishing an immune-suppressive tumour microenvironment, cGAS activation can promote a transition toward a mesenchymal state^44^ and chemoresistance, favouring metastatic dissemination^45^. In addition, an experimentally derived interferon-related DNA damage resistance signature (IRDS), highly related to CytoDR, has been associated with resistance to chemotherapy and/or radiation across different cancers^46^. Hence, we hypothesize that endocytic-mediated tissue fluidification promotes the acquisition of chemoresistance and plastic EMT traits.

**Extended Data Fig. 9:**
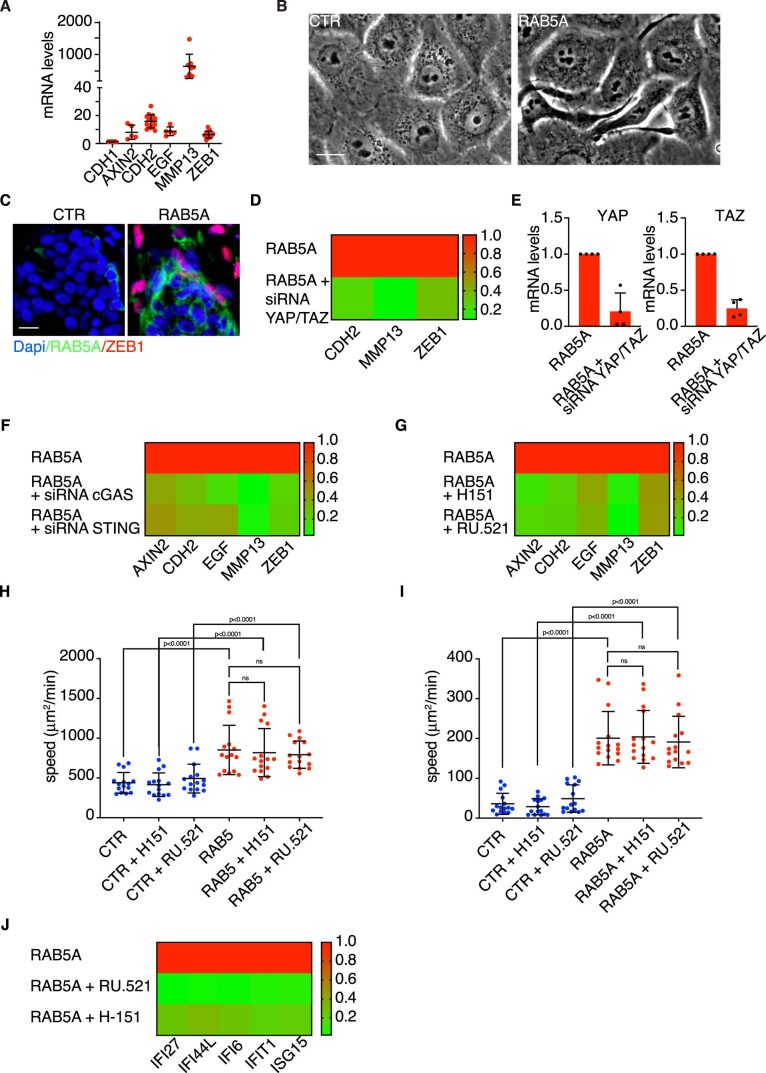
YAP1/TAZ and cGAS–STING-dependency of EMP gene activation, collective migration and invasion in RAB5A fluidized monolayers. **a**. mRNA expression levels of CDH1, AXIN2, CDH2, EGF, MMP13, and ZEB1 in RAB5A-MCF10.DCIS.com monolayers over control cells. Data are the mean ± s.d. (each dot represents an independent experiment). Values were normalized to the controls of each experiment. **b**. Phase-contrast images of control empty vector (CTR) and RAB5A-expressing (RAB5A) MCF10.DCIS.com monolayers (*n* = 5 independent experiments). Scale bar, 20 μm. **c**. Immunofluorescence images of doxycycline-treated control (CTR) and RAB5A-(RAB5A) MCF10.DCIS.com spheroids (a representative image from 5 independent experiments) stained as indicated. Scale bar 20 μm. **d**. Heatmap representing color-coded expression levels of differentially expressed EMT genes in RAB5A-MCF10.DCIS.com monolayers silenced for YAP and TAZ. The data are the ratio between the level of gene expression in each of the conditions tested relative to those of mock-treated RAB5A-cells. The mean ± s.d. (at least *n* = 3 independent experiments), two tailed Student’s t-test (siRNA versus RAB5A-expressing cells) are reported in Source data_Figure 9D. **e**. mRNA expression levels of YAP and TAZ in RAB5A-MCF10.DCIS.com over control cells silenced with the indicated oligos. Data are expressed as mean ± s.d. (*n* = 3 independent experiments). Values were normalized to the controls of each experiment. **f**. Heatmap representing color-coded expression levels of differentially expressed EMT genes in RAB5A-expressing MCF10.DCIS.com monolayers silenced for the indicated genes. This analysis was carried out on the same total mRNA from the experiment shown in Fig. 2a. The effectiveness of silencing is reported in Supplementary Fig. 4a. The mean ± s.d. (at least *n* = 3 independent experiments), two tailed Student’s t-test (siRNA versus RAB5A-expressing cells) are reported in Source data_Figure 9F. **g**. Heatmap representing color-coded expression levels of differentially expressed EMT genes in RAB5A-MCF10.DCIS.com monolayers treated with the following small molecules: cGAS inhibitor, 7 μg/ ml RU.521, or the STING antagonist, 4 μg/ ml H-151. This analysis was carried out on the same total mRNA from the experiment shown in Fig. 2b. The data are the ratio between the level of gene expression in each of the conditions tested relative to vehicle-treated RAB5A-expressing cells. The mean ± s.d. (at least n = 3 independent experiments) with two tailed Student’s t-test are reported in Source data_Figure 9G. **h**. Scatter plot of the speed of wound closure of control (CTR) and RAB5A-expressing MCF10.DCIS.com monolayers treated with vehicle or the cGAS inhibitor, 7 μg/ ml RU.521, or the STING antagonist, 4 μg/ ml H-151 (Supplementary Video 11). Data are the mean ± s.d. (*n* = 3 independent experiments, in which 5 FOV/condition/experiment were analysed), two-tailed Mann–Whitney non-parametric test. **i**. Scatter plot of control (CTR) and RAB5A-expressing MCF10.DCIS.com monolayers invading into a Matrigel coated wound and treated with vehicle or the cGAS inhibitor, 7 μg/ ml RU.521, or the STING antagonist, 4 μg/ ml H-151 (Supplementary Video 12). Data are the mean ± s.d. (*n* = 3 independent experiments, in which 5 FOV/condition/experiment were analysed), two-tailed Mann–Whitney non-parametric test, is indicated in the figure. **j**. Heatmap representing color-coded expression levels of differentially expressed CytoDR genes in RAB5A-MCF10.DCIS.com monolayers shown in Supplementary Fig. 10f treated with the indicated small molecules: cGAS inhibitor 7 μg/ ml RU.521 or the STING antagonist 4 μg/ ml H-151. The data are the ratio between the level of gene expression in each of the conditions tested relative to vehicle-treated RAB5A-expressing cells. The mean ± s.d. (*n*> 3 independent experiments) and two tailed Student’s t-test are reported in Source data_Figure 9J. P values are indicated in each graph. Source data

Firstly, we noticed that several mesenchymal markers, including CDH2, ZEB1, MMP13, EGF and AXIN2, were upregulated in RAB5A-expressing fluidized-via-flocking MCF10.DCIS.com (Extended Data Fig. 9a). Several canonical mesenchymal factors, including the master EMT regulators, SNAIL1 and 2 and TWIST, were unaffected, suggesting the acquisition of what has been defined as plastic EMT state (EMP)^47^. This was corroborated by the morphological analysis, which indicated that RAB5A expression leads to the acquisition of an elongated mesenchymal morphology (Extended Data Fig. 9b), and by the nuclear accumulation of ZEB1 in RAB5A-expressing MCF10.DCIS.com spheroids (Extended Data Fig. 9c). The expression of this set of genes was dependent on YAP/TAZ activity (Extended Data Fig. 9d,e), suggesting that the EMP phenotype switch is, at least in part, a mechanoresponsive process^48,49^.

As EMP is associated with invasion and metastasis, next, we tested whether the increased migratory and invasive capacity in RAB5A-fluidized collectives is mediated by the activation of cGAS and STING. We found, however, that either cGAS or STING inhibition, while effectively reducing the upregulation of ISG (Fig. 2a,b and Extended Data Fig. 9j), and, more relevantly, of EMP genes (Extended Data Fig. 9f,g), had no impact on wound migration or collective invasion (Extended Data Fig. 9h,i and Supplementary Videos 11 and 12). This finding indicates that endocytic-mediated tissue fluidization promotes collective motion and invasion. This is accompanied by increased mechanical stresses that results in frequent NE ruptures, the release of damaged DNA and the activation of cGAS–STING, which contributes to the acquisition of mesenchymal traits.

**Extended Data Fig. 10:**
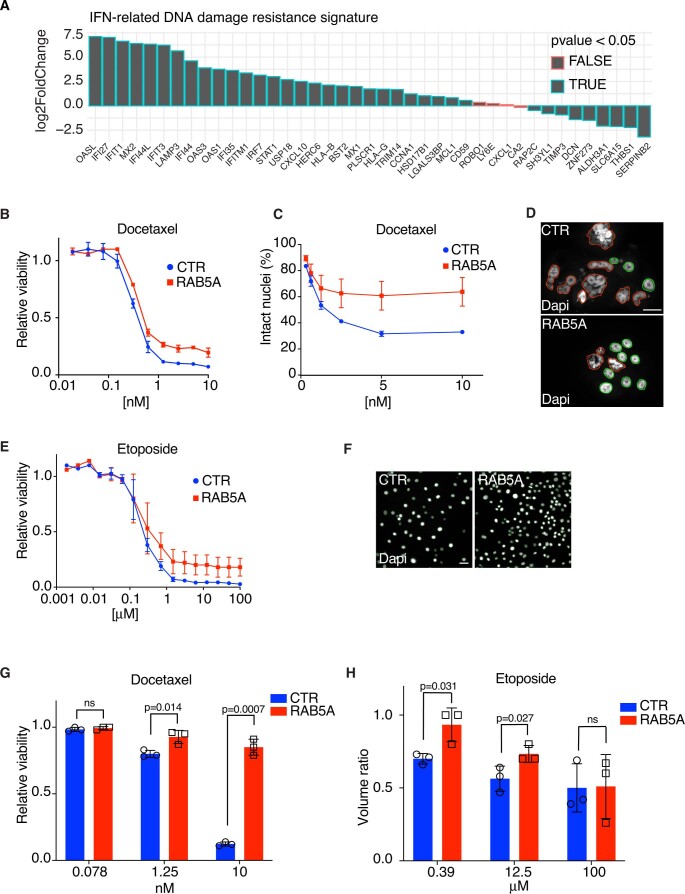
Tissue fluidification enhances chemoresistance. **a**. Quantitative changes in the expression of the INF-related DNA damage resistance signature genes in RAB5A-expressing MCF10.DCIS monolayers with respect to their respective controls. The log2Fold Change is plotted on the x-axis and the significance (Wald test p-value two-sided) is defined by the colour code of the outline. **b**. Dose-dependent analysis of cell proliferation of control (CTR) and RAB5A-expressing (RAB5A) MCF10.DCIS.com treated with the indicated concentrations of Docetaxel. Data are the mean ± s.d. (*n* = 3 independent experiments). **c**. Percentage of intact nuclei after treatment with the indicated doses of docetaxel in control (CTR) and RAB5A-expressing (RAB5A) MCF10.DCIS.com cells. Data are the mean ± s.d. (*n* = 3 independent experiments). Scale Bar is 20 μm **d**. Representative images of control empty vector (CTR) and RAB5A-expressing (RAB5A) MCF10.DCIS.com nuclei in the presence of 10nM Docetaxel. Intact and aberrant polynucleated cell nuclei are indicated by green and red contours. Scale bar 10 μm. **e**. Dose-dependent analysis of cell proliferation of control (CTR) and RAB5A-expressing (RAB5A) MCF10.DCIS.com treated with the indicated concentrations of Etoposide. Data are the mean ± s.d. (*n* = 3 independent experiments). **f**. Representative images of control empty vector (CTR) and RAB5A-expressing (RAB5A) MCF10.DCIS.com nuclei in the presence of 0.5 μM Etoposide. Scale bar 30 μm. **g, h**. Box Plot of the cell viability (by counting intact nuclei) and cell proliferation (measured as relative volume of spheroids) of control (CTR) and RAB5A-expressing (RAB5A) MCF10.DCIS.com spheroid grown over a Matrigel layer treated with the indicated concentrations of Docetaxel or Etoposide. Data are the mean ± s.d. (*n* = 3 independent experiments), unpaired one-tailed t-test with Welch’s correction. P values are indicated in each graph. Source data

Finally, we determined whether the elevation of CytoDR is associated with chemoresistance to anti-tumorigenic drugs. Firstly, GSEA in control and fluidized monolayers revealed the enrichment of an interferon-related DNA damage resistance signature, previously associated with resistance to chemotherapy and/or radiation^46^ (Extended Data Fig. 10a). Additionally, RAB5A-cells were slightly more resistant to the microtubule stabilizer, docetaxel (Extended Data Fig. 10b–d), and the topoisomerase inhibitor, etoposide (Extended Data Fig. 10e,f). In the former case, while most control cells display a grossly defective nuclear morphology, as expected, more than 60% of RAB5A-expressing cells displayed intact and unperturbed nuclei (Extended Data Fig. 10c,d). Increased chemoresistance to docetaxel and etoposide was also detected in 3D spheroids (Extended Data Fig. 10g,h).

## Outlook

The tissue-level phase transition from a solid or jammed to a liquid-like or unjammed state has been recently proposed to be a complementary or alternative gateway to cell invasion in both normal epithelia during development^3^ and in solid carcinoma during malignant progression^2,4,8,50^. Indeed, the progression from an indolent, quasibenign ductal breast carcinoma lesion to invasive ductal carcinoma is associated with the acquisition of a flocking-fluid mode of collective motion induced by the upregulation of the endocytic, promigratory gene, *RAB5A*^2,10^. In addition, we show here, that the altered mechanics of fluidized tissues impact nuclear integrity, and promote DNA release into the cytoplasm that results in a robust, cell-autonomous and long-lived transcriptional rewiring toward a cGAS–STING-dependent, pro-inflammatory response. This response has potential, context-dependent, far-reaching consequences in shaping the fate of tumour cells and cells of the microenvironment. Indeed, this cytosolic DNA response axis has recently been shown to induce a pro-tumorigenic phenotype, characterized by a shift toward a mesenchymal state and increased chemoresistance^44,45^, as we also found in our system. It is, however, likely that in an immune-proficient context, the same axis might trigger a pro-immunogenic, potentially anti-tumoral response. Whether this is the case is certainly a matter of future investigation. Similarly, it will be paramount to determine what are the factors or conditions that tune the mechanically driven cGAS–STING activation toward either a pro-tumorigenic or pro-immunogenic fate.

## Methods

A list of antibodies and dilutions is in Supplementary Table 1. Reagents, oligos and QRT-PCR assays are detailed in Supplementary Table 2. A list of organoids with relevant information is in Supplementary Table 3. Supplementary Methods are in Supplementary Information.

### Cell streaming assay

As previously shown^2^, cells were seeded in six-well plates (1.5 × 10^6^ cells per well) in complete medium and cultured until a uniform monolayer had formed. RAB5A expression was induced, where indicated, 16 h before performing the experiment by adding fresh complete media supplemented with 2.5 μg ml^–1^ doxycycline hyclate to cells. Comparable cell confluence was tested by taking pictures by differential interference contrast imaging using a 10× objective and counting the number of nuclei per field. In the cell streaming assay, the medium was refreshed before imaging began. An Olympus ScanR inverted microscope with 10× objective was used to take pictures every 5–15 min over a 24–48 h period. The assay was performed using an environmental microscope incubator set to 37 °C and 5% CO_2_ perfusion. After cell induction, doxycycline hyclate was maintained in the medium for the total duration of the time-lapse experiment.

For plasma membrane tension perturbation by osmotic treatments, an equal volume of hypotonic buffer (H_2_O + 1 mM CaCl_2_ + 1 mM MgCl_2_) was added to cell monolayer before performing experiments.

### Wound healing

For wound healing experiments, confluent monolayers of MCF10.DCIS.com control and RAB5A-positive cells, were plated on 12-well plates, and were wounded by scraping with a pipette tip and then transferred immediately to the microscope stage-top incubator. For monolayer invading assay, wounds were coated with Matrigel before the time-lapse.

Time lapses were performed with a Leica Thunder Imaging System based on a Leica DMi8 inverted microscope equipped with a Leica DFC9000 GT sCMOS camera. The images were acquired with an HC PL APO 10× objective using Leica LAS X software. Wound healing maximal velocity of closure is calculated from the area covered over time extracted using a custom Fiji^51^ and Matlab code.

The area covered over time is fitted with two straight lines (https://github.com/aganse/MultiRegressLines.matlab/blob/master/regress2lines.m) the slope of the second straight line is used to estimate the maximal velocity of closure after a lag phase.

### 3D spheroid formation assay

MCF10.DCIS.com cells were plated on ultra-low-attachment-surface six-well plates (Corning, Cat# 3471) at a density of 5 × 10^3^ cells per well. Cells were grown in serum-free condition for 10 days by adding fresh culture media every 2 days. After 7 days, 2.5 μg ml^–1^ doxycycline hyclate was added to the medium to induce RAB5A expression. Doxycycline was maintained in the medium for 2 days and finally spheroids were collected and processed for total RNA extraction.

### Mammary fat pad tumour development in NSG mice

All animal experiments were approved by the OPBA (Organisms for the Well-Being of the Animal) of IFOM and Cogentech. All experiments complied with national guidelines and legislation for animal experimentation. All mice were bred and maintained under specific pathogen-free conditions in our animal facilities at Cogentech Consortium at the FIRC Institute of Molecular Oncology Foundation and at the European Institute of Oncology in Milan, under authorization from the Italian Ministry of Health (autorizzazione no. 604–2016). The maximal tumour size permitted by our ethical guideline is 200 mm^3^. None of the experiments exceeded this limit.

Control and RAB5A MCF10.DCIS.com cells were injected into female NOD.Cg-PrkdcscidIl2rgtm1Wjl/SzJ (commonly known as the NOD SCID gamma; NSG) mice between 6–10 weeks old as described previously^52^.

### Neutral comet assay

The neutral comet assay was performed as described previously^53^ following the manufacturer’s protocol (Trevigen). Comet tail moment was measured using OpenComet plugin for ImageJ^54^.

### Image acquisition

Time-lapse imaging of the motility of 3D organoids was performed using a Leica TCS SP8 laser confocal scanner mounted on a Leica DMi8 microscope equipped with motorized stage; a HC PL FLUOTAR 20×/0.5 NA dry objective was used. A white-light laser was used as the illumination source. Leica Application Suite X (LAS X, https://www.leicamicrosystems.com/products/microscopesoftware/details/product/leica-las-x-ls/) software was used for all acquisitions. Image acquisition conditions were set to remove channel crosstalk, optimizing spectral detection bands and scanning modalities. ImageJ software was used for data analysis.

Image acquisition was performed using Operetta CLS, high-throughput imaging confocal microscopy system (Perkin Elmer) with Harmony software 4.9. Cells are imaged with 20× water immersion objective NA 1.0.

Confocal microscopy was performed with a Leica TCS SP5 confocal laser scanning system based on a Leica DMI 6000B inverted microscope. The images were acquired with an HCX PL APO 63X/1.4NA oil immersion objective. The software used for all acquisitions was Leica LAS AF. Laser lines: 405 nm, 488 nm, 561 nm, 633 nm.

Hypotonic-mediated cell streaming, and EGFP-3NLS leakage time lapses were performed with a Leica Thunder Imaging System based on a Leica DMi8 inverted microscope equipped with a Leica DFC9000 GT sCMOS camera. The images were acquired with an HC PL APO 63×1.4NA oil immersion objective (EGFP-3NLS time lapse) using Leica LAS X software.

Image acquisition of cGAS expression and localization on FFPE samples was performed with an Olympus BX63 full motorized wide field microscope equipped with a B/W Hamamatsu Orca_AG camera. the system is driven by Metamorph (Molecular Devices) software. We used UPlanApo 100× objective N.A.1.35.

### Growth assay survival and broken nuclei discrimination

To evaluate the growth of MCF10.DCIS control empty vector mCherry-H2B or RAB5A mCherry-H2B a Harmony 4.9 (PerkinElmer) custom pipeline was implemented. After 3 days of treatment, the images were acquired. For each well (four wells each condition) composed of 89 fields, the pipeline identifies the nuclei on the Gaussian filtered (radius 3 pixels) global image of mCherry channel using the B method of the Find Nuclei module (parameters were tuned condition by condition). Then the nuclei were filtered by intensity and morphological criteria. To discriminate broken and normal nuclei, the Linear Classifier module with two classes was used; the classifier was trained using around 30 nuclei for both classes.

### Image analyses

To count the number of foci per nuclei, a custom semi-automated Fiji^51,55^ plugin was developed. The plugin identifies the DAPI/nuclear marker using Li (https://imagej.net/plugins/auto-threshold#li) Thresholding Schema on the filtered image (Gaussian filter with 2 pixel radius). Nuclei are then split using the watershed (https://imagej.net/plugins/classic-watershed) method and then checked and corrected by hand. For each nucleus, the plugin identifies and counts the foci on the Foci Channel Marker (53BP1 or γH2AX) using ImageJ’s Find Maxima (https://imagej.nih.gov/ij/docs/menus/process.html#find-maxima) plugin with the noise tolerance parameter selected by hand.

To count the number of micronuclei per field of view (FOV), a custom semi-automated Fiji^51,55^ plugin was developed. The plugin identifies the DAPI/nuclear marker using Huang (https://imagej.net/plugins/auto-threshold#huang) thresholding schema on the filtered image (median filter with radius of 1 pixel). Nuclear structures were then split using the watershed (https://imagej.net/plugins/classic-watershed) method and then checked and corrected by hand. For each FOV, the plugin identifies and counts micronuclear structures using ImageJ’s Analyze Particles (https://imagej.net/imaging/particle-analysis) plugin with the size parameters selected by hand.

For assessing histone methylation on lysine 27, FOVs were randomly selected based on nuclei signal, probed by DAPI staining. Images were analysed using a custom semi-automated plugin developed in Fiji^51,55^. Nuclei were identified on the DAPI channel using the StarDist plugin (https://imagej.net/plugins/stardist) with the built-in Versatile (fluorescent nuclei) neural network model. For each nuclear region of interest, the mean intensity was measured on the H3K27me3 channel and then normalized on the median of the mean intensity distribution of control cells.

For the analysis of the differential signal intensity at the nuclear periphery and central region, images were acquired, and nuclei were segmented as described above. For each nuclear region of interest, the area was reduced to shrink it 1.5 µm from the nuclear border and the mean intensity in the H3K27me3 channel was calculated in the central nuclear region. Finally, the peripheral H3K27me3 mean intensity was calculated in the area between the central region and the nuclear border.

### Cell area fluctuation analysis

EGFP-E-cadherin expressing control and RAB5A-MCF10A cells were generated as described here. Cells were seeded in six-well plates (1.5 × 10^6^ cells per well) in complete medium and cultured until a uniform monolayer had formed. RAB5A expression was induced, where indicated, 16 hours before performing the experiment by adding fresh complete media supplemented with 2.5 μg ml^–1^ doxycycline to cells. Comparable cell confluence was tested by taking pictures by differential interference contrast (DIC) imaging using a 10× objective and counting the number of nuclei per field. To monitor cell fluctuations the phase contrast channel and EGFP-E-Cad channel were merged and then the 2D image sequences were converted into 3D image. Based on the phase contrast images and the E-Cad signal, randomly selected cells are segmented and tracked semi-automatically using Segmentation Editor in Fiji (ImageJ plugin). Cell boundaries were annotated manually with the interval of a few time points and then cell boundaries at other time points are interpolated in 3D to obtain accurate cell morphological dynamics along time. As we have excellent temporal resolution, we assume that any deformation along the direction perpendicular to the cell boundary is small enough such that we can treat it as linear. Thus, we can estimate the deformation of a given cell along time. The negative and positive cell extension of the cell, as shown in Fig. 2f, can be quantitatively extracted. For a migrating cell, its surface can be described as a function of time, that is *S*(*x*, *y*, *t*). The deformation between any consecutive-time points is captured by the partial derivative of *S* with respect to *t*:

1 $${\Delta}S = \frac{{\partial S}}{{\partial t}}$$

Based on our linear assumption, equation (1) can be used to find a linear minimum distance mapping for the points on the boundaries at two time points.

We also need to define whether the deformation is positive (extending—maximum positive deformation (MPD)) or negative (retracting—maximum negative deformation (MPD)). A positive deformation corresponds to a boundary point moving to a position not previously occupied by the cell, and is indicated by a red arrow in Fig. 2f. A negative deformation corresponds to a boundary point moving to a position previously occupied by the cell, and is indicated by a blue arrow in Fig. 2f. To quantify the cell mobility, we focused on the following parameters:

1. Maximum deformation: the maximum norm of the positive deformation vectors, shown as a solid red arrow in Fig. 2f
2. Minimum deformation: the maximum norm of the negative deformation vectors, shown as a solid blue arrow in Fig. 2f

### Nuclei tracking and segmentation

Tracking and segmentation of single nuclei in sequencies of fluorescent microscopy images of confluent monolayers of mCherry-H2B cells is performed with a custom Matlab code implementing the following procedure.

Images are first corrected for background intensity inhomogeneities by applying the background removing algorithm described previously in detail^56^.

Random noise in each corrected image is then reduced by applying a Wiener filter, an adaptive noise-removal filtering that preserves nuclei edges while smoothing the white noise (see equations 9.44–9.46 in ref. ^57^).

Nuclear segmentation is obtained by applying a seeded watershed transform to the spatial gradient of each filtered image^58^. The ‘seeds’ (that is, the pixels that are set to zero in the image before applying the watershed transform) are determined as follows. A Laplacian-of-the-Gaussian (LoG) filter is applied to each filtered image, leading to a map *L*_G_ whose local minima correspond to the candidate centres of the nuclei. Differences in the fluorescent intensity of different nuclei are corrected by dividing *L*_G_ by an intensity map obtained via bicubic interpolation of the minima of *L*_G_. The resulting map $\tilde L_\mathrm{G}$ is binarized by setting to zero (one) all pixels whose value is above (below) a fixed threshold value, *k*. Repeated pixel erosion operations are applied to the obtained binary mask to remove the smallest features and partially separate overlapping nuclei, leading to a final binary map *L*_BN_ from which we extract the ‘seeds’ to be used in the seeded watershed transform: internal ‘seeds’ are obtained as the pixels where *L*_BN_ is non-zero, while external ‘seeds’ as obtained the boundaries of the watershed transform of *L*_BN_.

Once the segmentation procedure on a given image is completed, we can determine the centre of mass, $\mathbf{x}_i$, of each nucleus in the image, its projected area *A*_*i*_ and the angle *θ*_*n,i*_ (modulo *π*) between the major axis of the nucleus and the *x* axis. The direction of the major axis is obtained as the direction of the eigenvector of the covariance matrix of the segmented area associated with the larger eigenvalue^59^.

To reconstruct cell trajectories, we employ the Matlab code freely available at http://site.physics.georgetown.edu/matlab/ implementing the algorithm developed by Crocker and Grier^60^. Once nuclei in different frames are linked into trajectories, the time evolution of the relevant single-nucleus parameters $\mathbf{x}_i \left( t \right)$, $A_i\left( t \right)$ and $\theta _{n,i}\left( t \right)$ can be determined.

The instantaneous velocity of the *i*-th nucleus at frame *t* is estimated as $\mathbf{v}_i \left( t \right) =\left( \mathbf{x}_i\left( t+1 \right) - \mathbf{x}_i \left( t-1 \right) \right) / 2\delta t$, where δ*t* is the time interval between two consecutive frames. The instantaneous mean migration velocity is computed as $\mathbf{v}_{cm} \left(t\right) =\left\langle \mathbf{v}_i \left(t\right) \right\rangle_i$, where $\left\langle \ldots \right\rangle _{i}$ denotes the average over all the nuclei in the field of view (FOV). The amplitude of the velocity fluctuations is evaluated as the root mean square velocity of the nuclei in the centre of mass reference frame $\mathbf{v}_{rms} \left(t\right) = \sqrt{\left\langle \left| \mathbf{v}_i \left(t\right) - \mathbf{v}_{cm} \left(t\right)\right|^2 \right\rangle_i}$.

The velocity spatial correlation function is calculated as$C_{vv}\left(r\right) = \left\langle \left\langle \frac{\mathbf{v}_i\left(t\right)\cdot \mathbf{v}_j\left(t\right)}{\left|\mathbf{v}_i\left(t\right)\right|\cdot \left| \mathbf{v}_j\left(t\right)\right|} \delta\left(\left|\mathbf{x}_i\left(t\right)-\mathbf{x}_j\left(t\right)\right|-r\right) \right\rangle_{i,j}\right\rangle_t$ where *i* and *j* run over all the nuclei and *t* is averaged over the time window 4–20 h, unless otherwise indicated. An estimate of the correlation length *L*_c_ of the velocity field is obtained by fitting a stretched exponential model $\mathrm{e}^{ - \left( {\frac{r}{{L_\mathrm{c}}}} \right)^\alpha }$ to *C*_*vv*_ (*r*).

Visual inspection reveals that the described segmentation procedure is effective in identifying about 90–95% of the nuclei present in the field of view. Despite the effort to reduce multiple segmentation and nuclei merging, however, some segmentation errors occur, especially in those cases where the signal-to-noise ratio is low or partial superpositions of nuclei are frequent. To minimize the impact of segmentation errors on the analysis of nuclear features, we implemented a ‘quality filter’ to remove potentially flowed measurements. To this end, we compute the total instantaneous intensity *J*_*i*_ (*t*) integrating the image intensity $I\left(\mathbf{x},t\right)$ over the segmented area of the *i*-th nucleus at frame *t*. We then compare the instantaneous value *J*_*i*_ (*t*) with its median ${\mathrm{med}}\left[J_i\left(t'\right)\right]_{t-11}^{t-1}$ evaluated over the previous 10 frames. If the quantity $\frac{\left|\mathrm{med}\left[J_i\left(t'\right)\right]_{t-11}^{t-1} - J_i\left(t\right) \right|}{\mathrm{med}\left[J_i\left(t'\right)\right]_{t-11}^{t-1}}\,$ is larger than 0.1, the segmentation of the *i*-th nucleus at frame *t* is considered unreliable and the corresponding parameters are not included in the statistics. Trajectories that, after the application of this ‘quality filter’, lose more than 20% of frames because of this procedure are entirely excluded.

### Particle image velocimetry

Particle image velocimetry (PIV) of fluorescent microscopy images of confluent monolayers of mCherry-H2B cells fluorescent images is performed by using the Matlab PIVLab software^61^.

We choose an interrogation area with size slightly larger than the typical inter-nuclear distance, typically corresponding to 14 μm. Outliers in the reconstructed velocity field, whose modulus exceeds a fixed threshold value, are identified, and replaced with the median value of the velocity over neighbouring grid points.

### Nuclear deformation dynamics

To characterize nuclear shape fluctuations, we evaluate the mean square nuclear strain $\mathrm{MSS}\left( \tau \right) = \left\langle {\left\langle {{\Delta}a_i^2(\tau |t)} \right\rangle _t} \right\rangle _i$ for different delay times *τ*. The nuclear strain ${\Delta}a_i\left( {\tau {{{\mathrm{|}}}}t} \right)$ is estimated as $\left[ {A_i\left( {t + \tau } \right) - A_i\left( t \right)} \right]/\left\langle {A_i\left( t \right)} \right\rangle _t$, where *A*_*i*_(*t*) is the projected area of the *i*-th nucleus at time *t*. To extract the key parameters characterizing nuclear deformation, we fit the model function $\mathrm{MSS}\left( \tau \right) = \sigma_\mathrm{w} + \dot \gamma _0\tau_\mathrm{c}\left[ {1 - \mathrm{e}^{ - \tau /\tau _\mathrm{c}}} \right]$ to the data. This model, which includes a term *σ*_w_ accounting for the random noise in determination of the projected area, describes a diffusive-like growth of the area fluctuations with a characteristic strain rate $\dot \gamma _0$ for short delay times $\mathrm{MSS}\left( \tau \right) \sim \dot \gamma _0\tau$, followed by a saturation to a plateau value $\dot \gamma _0\tau _\mathrm{c}$ for long times. In Fig. 2g,h, the data and the best fitting curves are reported upon the subtraction of the baseline value *σ*_w_ obtained from the fitting procedure. Since MSS(*τ*) does not always reach a clear plateau within the time window accessible during the experiments (Fig. 2g,h), there is a relatively large uncertainty in the determination of the overall amplitude, $\dot \gamma _0\tau _\mathrm{c}$, of the fluctuations. However, this does not affect the robustness of the estimate of $\dot \gamma _0$, as it characterizes the short time behaviour of the fluctuations, which is accurately sampled in our experiments.

### Estimation of the relative stiffness of the nuclei

To characterize the mechanical response of cell nuclei to intracellular stresses induced by mutual cell displacements, we independently evaluate nuclear and cell deformations by measuring the instantaneous nuclear strain rate using the automated imaging segmentation pipeline described above, and the corresponding instantaneous cell strain rate, obtained after computing the divergence of the velocity fields measured by PIV analysis^36^.

### Atomic force microscopy measurements

AFM measurements were carried out at 37 °C using a NanoWizard3 AFM (JPK, Grermany) mounted on an Olympus inverted microscope. The protocol was adapted from a previous study^62^.

Prior to AFM measurements, MCF10.DCIS.com cells, control or RAB5A-induced, were seeded as a monolayer on 24 mm glass coverslips.

### Cell stretching experiments

Cell stretching experiments were carried out using an automated cell stretching dish (international patent: WO 2018/149795)^63^.

The components of the device were designed using SolidWorks CAD software and 3D printed using a stereolithography-based 3D printer (Form 2, Formlabs) and a biocompatible and autoclavable dental resin (Dental SG resin, Formlabs). The printed parts were washed with isopropyl alcohol to remove eventual uncured resin and then post-cured in a UV box to complete the polymerization process. The 3D printed parts were then polished and assembled to create the lower and the upper portions of the stretching dish.

The lower portion (cell culture chamber) has four clips clamping a deformable silicone membrane (thickness, 200 µm, Silex Silicones) sandwiched between two rings. The upper portion (aperture driver) consists of stretching means movable relative to the chamber. This is coupled to a motor controller allowing to regulate the simultaneous movement of the stretching means and then apply the required stress/strain to the membrane seeded with cells. According to previous tests, the strain field is uniform in the central region of the dish within 6 mm^2^.

Prior to the experiments, the cell culture chamber of the stretching device was coated with fibronectin (20 µg ml^–1^). Control and RAB5A-induced MCF10.DCIS cells were seeded as a monolayer.

Before imaging, the whole cell stretching dish was assembled by connecting the aperture driver of the stretching dish to the cell culture chamber. Biaxial stretching was applied directly on the stage of the microscope. Image acquisition was performed using a Confocal Spinning Disk system (Olympus) mounted on an IX83 inverted microscope provided with a motorized stage and an IXON 897 Ultra camera (Andor, 16 bit, pixel size 16 µm), and driven by CellSens Dimension software. Fluorescence images were acquired before and after application of 22% biaxial strain (reached in six stretching steps) through a 20× objective (UPlansApo, NA 0.75) using the EPI-fluorescence mode (excitation wavelength: 530–550 nm). For both control and RAB5A-expressing cells, 30 regions (field of views) were considered within the central region of the cell stretching dish. For each field of view, we evaluated the fractional change ${\Delta}a = \left( {A_f - A_i} \right)/A_i$ in the average nuclear-projected area upon stretching. Exploiting the mechanical model in ref. ^36^, we estimate the ratio between the effective elastic moduli *E*_N_ and *E*_CY_ of the nucleus, and the cytoplasm, respectively, as

$$E_\mathrm{N}/E_\mathrm{CY} = \left( {{\it{\epsilon }}_\mathrm{tot}/{{{\mathrm{{\Delta}}}}}a - \beta } \right)/\left( {1 - \beta } \right),$$

where ${\it{\epsilon }}_\mathrm{tot} = 1.28 \times 1.28 \cong 1.49$ is the imposed area strain and *β* is the surface fraction covered by the nuclei. In our experiments, *β* was found to vary in the range [0.25, 0.31] and [0.17, 0.24] for MCF10.DCIS control and RAB5A-expressing cells, respectively.

### Nuclear deformation through constricted channels

To evaluate nucleus deformability suspended cells were passively flowed, at a concentration of 100,000 cells ml^–1^ and a flow rate of 5 µl min^–1^, into microchannels of 25×20 µm size with a constriction of 6×20 µm that induces substantial nuclear deformation. The microfluidic device was obtained from a micro-structured silicon mould fabricated at the clean room facilities of the Binning and Rohrer Nanotechnology Center through standard photolithography and dry etching processes. The microfluidic chip was then fabricated in PDMS (Sylgard 184) through standard replica moulding. Briefly, the PDMS precursor was mixed with the crosslinker (10:1) and poured on the silicon mould, degassed for 1 h in a vacuum bell and then cured for 3 h at 90 °C. The chip was then demoulded, treated for 1 min with oxygen plasma and irreversibly bonded to a 35 mm bottom-glass petri dish (Mattek).

Experiments were performed at 37 °C and 5% CO_2_ atmosphere. Nuclear squeezing was recorded using a LEICA widefield DMI8 inverted system equipped with a HC PL Fluotar 10× NA = 0.32 (Leica, #506522) objective. The excitation source was a solid-state LED illumination at 475/28 nm (Lumencor light engine LED8). Images were each acquired for 50 ms with a sCMOS Andor Neo 5.5 camera.

To quantify nuclear deformability, we measured the speed of aspect ratio (AR) variation of control and RAB5A-expressing cells. Nuclei were labelled with mCherry-H2B. AR is a dimensionless parameter, and its rate of change provides a direct measurement of nuclear deformability, with higher values corresponding to more deformable (that is, softer) nuclei. We measured nuclei AR for several cells in the time interval needed to pass from an undeformed, just before entering into the constriction, to a completely squeezed configuration (Supplementary Fig. 6e, from *A*/*R*_0_ at *t*_0_ to *A*/*R*_*f*_ at *t*_*f*_). Images were analysed with ImageJ and the nuclear AR variation rates extrapolated through a robust fit in R using the ‘robustbase’ package^64^, where the rate of AR changes as a function of time and is the slope of the fitted curve.

### Excess of perimeter and NE fluctuations

The dimensionless parameter excess of the perimeter of the projected nuclear shape (EOP) was determined through the analysis of immunofluorescent images of SUN2, an inner nuclear membrane protein^38^. As described previously^65^, to determine EOP, we first obtained values for perimeter (*P*) and surface area (*A*) from 2D projected images taken at the maximum radius of the nucleus (using SUN2 stained nuclei). Next, we introduced *R*_0_ as the radius of the circle defined by the area *A*, and compute EOP as the ratio between (*P* − 2π*R*_0_) and (2π*R*_0_). EOP values of a highly folded, presumably floppy, and soft object tend to be close to 1, whereas EOP of a rigid object with a smooth surface tends to be close to 0.

Nuclear envelope fluctuations were measured following the method described in ref. ^65^. Nuclear envelope images of both control and Rab5 populations were recorded using an HC PL APO 63× NA 1,40 OIL immersion objective (Leica, #506350), on a Leica DMI8 widefield Thunder Imager, equipped with a Leica DFC9000 GTC sCMOS camera.

A 488 LED illumination was used to record short time-lapses of about 2 min with high frame rate (4 fps).

The positions of each nucleus have been corrected for the natural linear and rotational motion of the cells using the Stackreg plugin available on Fiji software^66^.

Nuclear envelope fluctuations are measured as the standard deviation from its mean position. Eight separate line scans were drawn orthogonally along the surface of the nucleus. A simple macro (stackprofile_Pala)—modified from a version found on the ImageJ website^55^ by Michael Schmid—was used to determine the profile of each line scan for each time point in the video. The standard deviation of the position of the nuclear envelope around the mean position of all timepoints is taken as a measure of the fluctuation of the nuclear envelope. Each point along a nucleus contributes as one measure. Obtained values are expressed in μm. *N* = 10 cells for both populations, for a total of *n* = 80 measures.

### Imaging and direct particle tracking

For the analysis of genetically encoded viral particles, control or RAB5A-expressing MCF10A and MCF10.DCIS.com cells were lentivirally infected with pLH1337-CMV-PfV-Sapphire-IRES-DsRed-WPRE (Addgene).

Single particle tracking was performed for 40 nM GEMs using a Perkin Elmer Spinning Disk Confocal Microscope and fluorescence was recorded with a C9100-50 (EMCCD) Camera and a 100× objective at a 20 ms image capture rate. The tracking of particles was performed with the Mosaic suite of FIJI using the following typical parameters: radius = 3, cut-off = 0.001 of fluorescence intensity, a link range of 1, and a maximum displacement of 5 px, assuming Brownian dynamics.

### Extraction of the rheological parameters

For every trajectory, we calculated the time-averaged mean square displacement as defined previously^37,67^. To characterize the individual particle trajectories, we calculated apparent diffusion coefficients by fitting mean square displacement with linear (diffusive) time dependence at short time scales as shown previously^37^.

### Brillouin microscopy

Control and RAB5A-MCF10.DCIS.com cells treated with doxycycline were grown in adhesion on a glass coverslip. Cells were analysed by Brillouin micro-spectroscopy as described previously^68^. Briefly, the 53C2 nm monochromatic beam of a single mode, diode-pumped, solid state laser (Spectra-Physics Excelsior) is focused with a power less than 5 mW. The investigated position inside the cell nuclei was chosen using the correlated bright field image of a custom-made inverted microscope.

The same water immersion 60× objective (UPLSAPO-60XW from Olympus) was used to acquire bright field images, to focalize the laser beam and to collect the back-scattered light, which is analysed in frequency by the TFP-2 interferometer^68^. The archived spatial resolution of the mechanical characterization was in the micrometric length scale^69^.

The Brillouin frequency was extracted fitting the spectra using a damped harmonic oscillator function. Measurements of the longitudinal elastic moduli were obtained after setting the cellular density at *ρ* = 1,080 kg m^−3^, and the refractive index at *n* = 1.386 as described previously^68^.

### Statistical analysis

All data are presented as scatter plots or box plots expressed as mean ± s.d. unless otherwise indicated. The number of experiments as well as the number of samples analysed is specified for each experiment and reported in the figure legends. Statistical significance was calculated, whenever we compared two distinct distributions, using a parametric two-tails unpaired student’s *t*-test with Welch corrections for two samples with unequal variance or non-parametric two-tailed Mann–Whitney *t*-test as indicated. Kruskal–Wallis/Dunn’s test was used for one-way data with more than two groups. Nested one-way ANOVA was used as reported for comparison of more unmatched groups. Statistical calculations were performed in GraphPad Prism 8 or Microsoft Excel. The significance of fold difference of each differential gene expression obtained by QRT-PCR was established using a two-tailed unpaired Student’s *t-*test with Welch corrections for two samples and were with *P* values > 0.05 in all cases. Data collection and analysis were performed blind to the conditions of the experiments. Specifically, in all experiments involving mice, we assigned each mouse randomly to the treatment groups (injection of control or RAB5A cells into mammary fat pads). For the experiment with cells, we had two genetically distinct groups (control versus RAB5A) that were treated equally and randomly. For the CLEM experiment, we selected blindly control or RAB5A cells displaying accumulated perinuclear cGAS. No data points were excluded.

### Reporting summary

Further information on research design is available in the Nature Portfolio Reporting Summary linked to this article.

## Online content

Any methods, additional references, Nature Portfolio reporting summaries, source data, extended data, supplementary information, acknowledgements, peer review information; details of author contributions and competing interests; and statements of data and code availability are available at 10.1038/s41563-022-01431-x.

## Supplementary information

Supplementary Information Legends to Supplementary Videos 1–13, Supplementary Fig. 1, Tables 1–3, discussion, methods and references.

Reporting Summary

Supplementary Video 1 Control (CTR) or RAB5A-MCF10.DCIS.com monolayers seeded at various densities in a 12-well plate, and monitored by time-lapse microscopy over 48 hours. Pictures were taken every 15 minutes (Extended Data Fig. 2c). Scale bar, 150 μm.

Supplementary Video 2 Control (CTR) or RAB5A-MCF10.DCIS.com monolayers expressing mCherry-H2B treated with hypotonic solution were monitored by fluorescence time-lapse microscopy over 48 hours. Pictures were taken every 15 minutes (Extended Data Fig. 2g). Scale bar, 150 μm.

Supplementary Video 3 Control or RAB5-expressing HaCat monolayers were seeded at a jamming density, serum starved for 2 days, doxycycline-treated and monitored by time-lapse phase-contrast microscopy in the presence or the absence of EGF (100 ng ml^–1^). Frames were acquired every 5 min over a period of 48 hours (Extended Data Fig. 3a–c). Scale bar, 100 µm.

Supplementary Video 4 RAB5A promotes cell fluctuations in confluent monolayer. Space and time cell fluctuations were monitored in MCF10A cells stably expressing EGFP-E-Cadherin by fluorescence time-lapse microscopy over 24 hours. Pictures were taken every 5 min and random pseudo colours are selected for different cell identities (Fig. 2e–g). Scale bar, 20 µm.

Supplementary Video 5 Nuclear segmentation and tracking of nuclear shape changes. Control and RAB5A-MCF10A expressing mCherry-H2B were monitored by fluorescence time-lapse microscopy over a 24 hour period. Pictures were taken every 10 min. The upper panels show randomly picked cell nuclei in which the continuous green lines with different shades of green represent the corresponding fluctuating profiles of nuclear contours obtained via nuclear segmentations (see Fig. 2h). In the bottom magnified panels, the red lines indicate the representative fluctuating profiles of nuclear contours of control and RAB5A-MCF10A cells in a monolayer. Scale bar, 4 μm.

Supplementary Video 6 Control and RAB5A-MCF10.DCIS.com monolayers expressing EGFP-3NLS were seeded at jamming density. After treatment with doxycycline to induce transgene expression, monolayers were monitored by fluorescence time-lapse microscopy over 30 hours. Picture were taken every 15 min. The leakage of EGFP-3NLS into the cytoplasm is indicative of NE ruptures (Fig. 3d,e) Scale bar, 15 μm.

Supplementary Video 7 Control (CTR) and RAB5A-MCF10.DCIS.com expressing mCherry-H2B that are passively going through a restricted 6 μm-wide channel were monitored by fluorescence time-lapse microscopy over 5 minutes. Pictures were taken every 50 msec (Extended Data Fig. 6e). Scale bar, 50 μm.

Supplementary Video 8 Control (CTR) and RAB5A-MCF10.DCIS.com expressing EGFP-Nesprin were monitored by fluorescence time-lapse microscopy over 5 minutes. Pictures were taken every 250 msec (Extended Data Fig. 6h). Scale bar, 10 μm.

Supplementary Video 9 Living breast cancer organoids labelled with NucLight and embedded into Matrigel were monitored by fluorescence time-lapse microscopy over a period of 24 hours. Pictures were taken every 15 min. Scale bar, 15 μm.

Supplementary Video 10 3D rendering of five living breast cancer organoids labelled with NucLight and embedded into Matrigel were monitored by fluorescence time-lapse microscopy over a period of 24 hours. Pictures were taken every 15 min. The size of each box along the *x*- and *y*-directions was 200 μm. In the bottom panels, thin blue, orange and yellow curves are the temporal evolution of the *x*, *y* and *z* components of the angular velocity associated with the rotation of the organoid depicted in the box above each panel, respectively. Thick black curves represent the angular speed, that is, the modulus of the angular velocity. Organoids are categorized as ‘rotating’ if their average angular speed is larger than 0.03 cycles per hour, as ‘non-rotating’ otherwise (Extended Data Fig. 9a–c).

Supplementary Video 11 Wound healing of control (CTR) and RAB5A-MCF10.DCIS.com seeded at jamming density were monitored by time-lapse microscopy for 48 hours. Pictures were taken every 15 min (Extended Data Fig. 10h). Scale bar, 150 μm.

Supplementary Video 12 Invasion into Matrigel of wounded control (CTR) and RAB5A-MCF10.DCIS.com seeded at jamming density were monitored by time-lapse microscopy for 48 hours. Pictures were taken every 15 min (Extended Data Fig. 10i). Scale bar, 150 μm.

Supplementary Video 13 A semi-automated image analysis pipeline to quantify the location of the γH2AX expressing cells in the tumoural ductal-adenocarcinoma regions.

Supplementary Data 1 Source data to Supplementary Fig. 1.

## Data Availability

RNA-seq data of MCF10A, MCF10.DCIS.com cells and organoids are deposited in the Gene Expression Omnibus (GEO) and European Genomephenome Archive (EGA), with the respective accession numbers: GSE183479 RNA-seq, GSE183539 SAMMY-seq, GSE183407 ChIP-seq, GSE205108 RNA-seq of organoids. Other data generated or analysed during this study are included in the Supplementary Information and are available from the corresponding authors upon request. Source data are provided with this paper.

## Source data

Source Data Fig. 1 Statistical source data.
Source Data Fig. 1 Uncropped scanned blots.
Source Data Fig. 2 Statistical source data.
Source Data Fig. 2 Uncropped scanned blots.
Source Data Fig. 3 Statistical source data.
Source Data Fig. 4 Statistical source data.
Source Data Fig. 5 Statistical source data.
Source Data Fig. 6 Statistical source data.
Source Data Extended Data Fig. 1 Statistical source data.
Source data. Extended Data Fig. 1c Uncropped scanned blots.
Source Data Extended Data Fig. 2 Statistical source data.
Source Data Extended Data Fig. 3 Statistical source data.
Source Data Extended Data Fig. 5 Statistical source data.
Source Data Extended Data Fig. 6 Statistical source data.
Source Data Extended Data Fig. 8 Statistical source data.
Source Data Extended Data Fig. 9 Statistical source data.
Source Data Extended Data Fig. 10 Statistical source data.

## Code availability

All custom code and scripts used in this study are available on request from the corresponding authors or online: Matlab for Nuclei dynamics and mechanics (10.5281/zenodo.7117355); Wound Healing analysis tool (10.5281/zenodo.7124712); plugins Fiji for micronuclei, yH2AX foci, H3K27me3 (10.5281/zenodo.7115350).
