# Author Correction: Tissue fluidification promotes a cGAS–STING cytosolic DNA response in invasive breast cancer

**DOI:** 10.1038/s41563-023-01479-3

**Published:** 2023-01-26

**Authors:** Emanuela Frittoli, Andrea Palamidessi, Fabio Iannelli, Federica Zanardi, Stefano Villa, Leonardo Barzaghi, Hind Abdo, Valeria Cancila, Galina V. Beznoussenko, Giulia Della Chiara, Massimiliano Pagani, Chiara Malinverno, Dipanjan Bhattacharya, Federica Pisati, Weimiao Yu, Viviana Galimberti, Giuseppina Bonizzi, Emanuele Martini, Alexander A. Mironov, Ubaldo Gioia, Flora Ascione, Qingsen Li, Kristina Havas, Serena Magni, Zeno Lavagnino, Fabrizio Andrea Pennacchio, Paolo Maiuri, Silvia Caponi, Maurizio Mattarelli, Sabata Martino, Fabrizio d’Adda di Fagagna, Chiara Rossi, Marco Lucioni, Richard Tancredi, Paolo Pedrazzoli, Andrea Vecchione, Cristiano Petrini, Francesco Ferrari, Chiara Lanzuolo, Giovanni Bertalot, Guilherme Nader, Marco Foiani, Matthieu Piel, Roberto Cerbino, Fabio Giavazzi, Claudio Tripodo, Giorgio Scita

**Affiliations:** 1 IFOM, the FIRC Institute of Molecular Oncology, Milan, Italy; 2 Department of Medical Biotechnology and Translational Medicine, University of Milan, Segrate, Italy; 3 Department of Health Sciences, Human Pathology Section, University of Palermo School of Medicine, Palermo, Italy; 4 Institute of Molecular and Cell Biology, A*STAR, Singapore, & Bioinformatics Institute, A*STAR, Singapore, Singapore; 5 European Institute of Oncology (IEO) IRCCS, Milan, Italy; 6 Istituto Officina dei Materiali, National Research Council (IOM-CNR), Unit of Perugia, c/o Department of Physics and Geology, University of Perugia, Perugia, Italy; 7 Department of Physics and Geology, University of Perugia, Perugia, Italy; 8 Department of Chemistry, Biology and Biotechnology, Biochemical and Biotechnological Sciences, University of Perugia, Perugia, Italy; 9 Institute of Molecular Genetics, National Research Council, Pavia, Italy; 10 Unit of Anatomic Pathology, Department of Molecular Medicine, Fondazione IRCCS Policlinico San Matteo, University of Pavia, Pavia, Italy; 11 Medical Oncology Unit, Fondazione IRCCS Policlinico San Matteo, Pavia, Italy; 12 Department of Internal Medicine and Medical Therapy, University of Pavia, Pavia, Italy; 13 Department of Clinical and Molecular Medicine, University of Roma, La Sapienza, Rome, Italy; 14 Institute of Biomedical Technologies, National Research Council, Milan, Italy; 15 National Institute of Molecular Genetics Romeo and Enrica Invernizzi, INGM, Milan, Italy; 16 Department of Pathology, S. Chiara Hospital, Azienda Provinciale per i Servizi Sanitari, Trento, Italy; 17 CISMed University of Trento, University of Trento, Trento, Italy; 18 Institut Curie and Institut Pierre Gilles de Gennes, PSL Research University, CNRS, UMR-144, Paris, France; 19 Department of Oncology and Haemato-Oncology, University of Milan, Milan, Italy; 20 Faculty of Physics, University of Vienna, Vienna, Austria; 21 Present Address: Max Plank Institute for Dynamics and Self-Organization, Göttingen, Germany; 22 Present Address: Dipartimento di Medicina Molecolare e Biotecnologie Mediche, Università degli Studi di Napoli Federico II, Naples, Italy; 23 Present Address: S.C. Oncologia Medica, ASST Melegnano e della Martesana, Ospedale Uboldo, Cernusco sul Naviglio, Milan, Italy; 24 Present Address: Cell Pathology Children’s Hospital of Philadelphia, Research Institute Department of Pathology and Laboratory Medicine University of Pennsylvania Perelman School of Medicine, Philadelphia, PA USA

**Keywords:** Cellular motility, Biomaterials - cells, Cancer

Correction to: *Nature Materials* 10.1038/s43588-01431-x. Published online 29 December 2022.

In the version of this article initially published, the first name of Flora Ascione was listed as “Floriana”; the error has been corrected in the HTML and PDF versions of the article.

