# Supplementary material for: Tissue fluidification promotes a cGAS–STING cytosolic DNA response in invasive breast cancer
Source: Nat Mater. 2022 Dec 29;22(5):644–55. doi: 10.1038/s41563-022-01431-x (PMC10156599; doi:10.1038/s41563-022-01431-x)
Supplement: Supplementary file 1 — Legends to Supplementary Videos 1–13, Supplementary Fig. 1, Tables 1–3, discussion, methods and references. [file 41563_2022_1431_MOESM1_ESM.pdf]

nature materials

Article

https://doi.org/10.1038/s41563-022-01431-x

Tissue fluidification promotes a cGAS–STING cytosolic DNA response in invasive breast cancer

In the format provided by the authors and unedited

This PDF file includes:

Supplementary Legends to Videos 1 to 13

Supplementary Figure 1

Extended data Tables 1 to 3 relative to Supplementary Methods

Supplementary Note (Discussion)

Supplementary Methods

Supplementary References

Supplementary Legends to Videos

Video1.

Control (CTR) or RAB5A-MCF10.DCIS.com monolayers seeded at various densities in a 12 wells plate, and monitored by time lapse microscopy over a 48 hours period. Pictures were taken every 15 minutes (see Extended data Fig. 2C). Scale bar, 150 μm.

Video2.

Control (CTR) or RAB5A-MCF10.DCIS.com monolayers expressing mCherry-H2B treated with hypotonic solution were monitored by fluorescence time-lapse microscopy over a 48 hours period. Pictures were taken every 15 minutes (see Extended data Fig. 2G). Scale bar, 150 μm.

Video3.

Control or RAB5-expressing HaCat monolayers were seeded at a jamming density, serum starved for 2 days, doxycycline-treated and monitored by time-lapse phase-contrast microscopy in the presence or the absence of EGF (100 ng/ml). Frames were acquired with every 5 min over a period of 48 hours (see Extended data Fig. 3A-C). Scale bar, 100 μm.

Video4.

RAB5A promotes cell fluctuations in confluent monolayer. Space and time cell fluctuations were monitored in MCF-10A cells stably expressing EGFP-E-cadherin by fluorescence time-lapse microscopy over a 24 hours period. Pictures were taken every 5 min and random pseudo colors are selected for different cell identities (see Fig. 2E-G). Scale bar, 20 μm.

Video5.

Nuclear segmentation and tracking of nuclear shape changes. Control and RAB5A-MCF10A expressing mCherry-H2B were monitored by fluorescence time-lapse microscopy over a 24 hour period. Pictures were taken every 10 min. The upper panels show randomly picked cell nuclei in which the continuous green lines with different shades of green represent the corresponding fluctuating profiles of nuclear contours obtained *via* nuclear segmentations (see Fig. 2H). In the bottom magnified panels, the red lines indicate the representative fluctuating profiles of nuclear contours of control and RAB5A-MCF10A cells in a monolayer. Scale bar, 4 μm.

Video6.

Control and RAB5A-MCF10.DCIS.com monolayers expressing EGFP-3NLS were seeded at jamming density. After treatment with doxycycline to induce transgene expression, monolayers were monitored by fluorescence time-lapse microscopy over a 30 hours period. Picture were taken every 15 min. The leakage of EGFP-3NLS into the cytoplasm is indicative of NE ruptures (see Fig. 3D-E) Scale bar, 15 μm.

Video7.

Control (CTR) and RAB5A-MCF10.DCIS.com expressing mCherry-H2B that are passively going through a restricted 6 micrometer-wide channel were monitored by fluorescence time-lapse microscopy over a 5 minutes period. Pictures were taken every 50 msec. (see Extended data Fig. 6E). Scale Bar, 50 μm.

Video8.

Control (CTR) and RAB5A-MCF10.DCIS.com expressing EGFP-Nesprin were monitored by fluorescence time-lapse microscopy over a 5 minutes period. Pictures were taken every 250 msec. (see Extended data Fig. 6H). Scale Bar, 10 μm.

Video9.

Living breast cancer organoids labelled with NucLight and embedded into Matrigel were monitored by fluorescence time-lapse microscopy over a period of 24 hours. Pictures were taken every 15 min. Scale Bar, 15 μm.

Video10.

3D rendering of five living breast cancer organoids labelled with NucLight and embedded into Matrigel were monitored by fluorescence time-lapse microscopy over a period of 24 hours. Pictures were taken every 15 min. The size of each box along the x- and y-directions corresponds to 200 μm. In the bottom panels, thin blue, orange and yellow curves are the temporal evolution of the x, y and z components of the angular velocity associated with the rotation of the organoid depicted in the box above each panel, respectively. Thick black curves represent the angular speed, *i.e.,* the modulus of the angular velocity. Organoids are categorized as "rotating" if their average angular speed is larger than $0.03\ \mathrm{cycles/h}$, as "non rotating" otherwise (see Extended data Fig. 9A-C).

Video11.

Wound healing of control (CTR) and RAB5A-MCF10.DCIS.com seeded at jamming density were monitored by time-lapse microscopy for 48 hours. Pictures were taken every 15 min. (see Extended data Fig. 10H). Scale Bar, 150 μm.

Video12.

Invasion into Matrigel of wounded control (CTR) and RAB5A-MCF10.DCIS.com seeded at jamming density were monitored by time-lapse microscopy for 48 hours. Pictures were taken every 15 min. (see Extended data Fig. 10I). Scale Bar, 150 μm.

Video13.

A semi-automated image analysis pipeline to quantify the location of the γH2AX expressing cells in the tumoral ductal-adenocarcinoma regions. A deep learning based nuclear segmentation (Stardist)[1] was employed to segment individual nuclei and identified the centroid positions of each nuclei in the image frame. A semi-automated analysis was used to identify the location of whole area, the core region and the outer margin of the ductal-adenocarcinoma. An automated histogram-based thresholding in each frame to identify regions with high expression of γH2AX signal, above the threshold applied. Isolated small spots were removed as noise. Nuclei that display either a positive

or weak/absent (below the arbitrary established threshold levels) γH2AX signal were identified. For each case, we quantified the total number of nuclei, the number of nuclei with strongly positive or weak/no γH2AX signal in the tumor core, tumor surface and in the whole tumoral areas and computed the percentage of nuclei with strongly positive γH2AX signal in the core region, tumor front regions and in the whole tumor region over 9 independent cases (see Materials and Methods for details).

Supplementary Figure 1

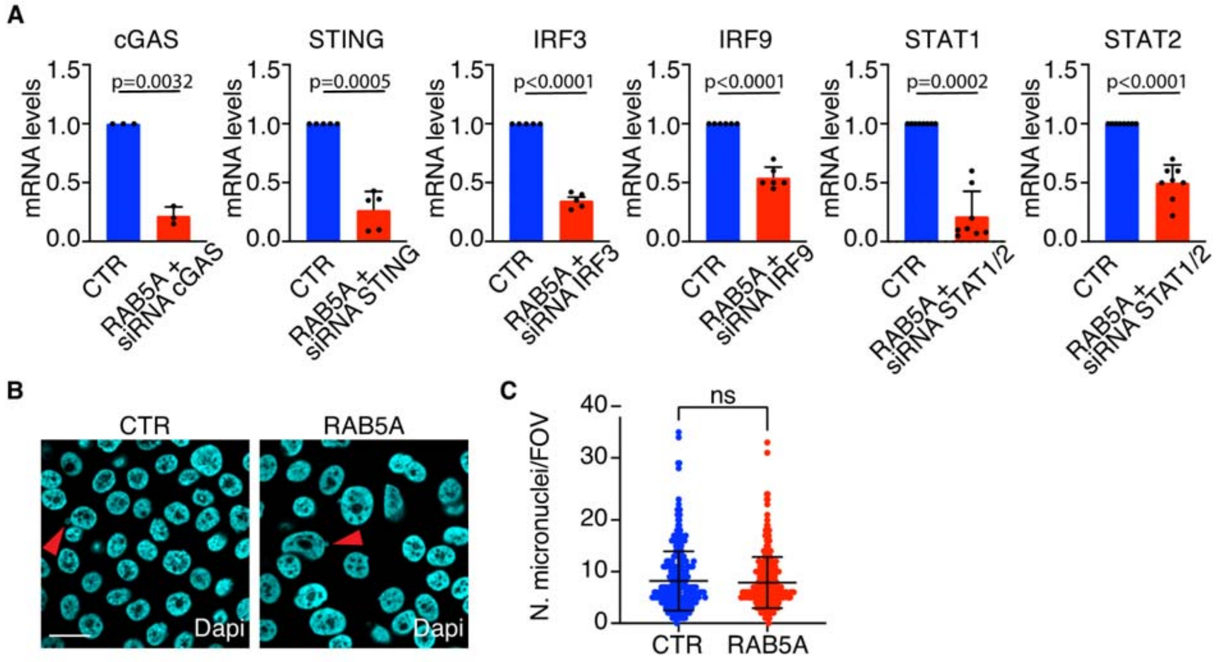

Supplementary-Figure 1. Expression of cGAS/STING pathway components and micronuclei analysis

A. Box plot of the mRNA expression levels of cGAS, STING, IRF3, IRF9, STAT1 and STAT2 determined by qRT-PCR in RAB5A-MCF10.DCIS.com monolayer over control cells silenced with indicated oligos. Data are expressed as mean ± s.d. (each dot represents RAB5A + siRNA cGAS *n*= 3, RAB5A + siRNA STING *n*= 5, RAB5A + siRNA IRF3 *n*= 3, RAB5A + siRNA IRF9 *n*= 3, RAB5A + siRNA STAT1 *n*= 8, RAB5A + siRNA STAT2 *n*= 8 independent experiments). Values were normalized to the controls of each experiment, two-tails, t-test with Welch's correction.

B. Representative images of nuclei and micronuclei stained with DAPI of control and RAB5A monolayers, micronuclei are indicated with red arrowheads. Scale bar, 15 μm

C. Number of micronuclei per field of view (FOV) in control (CTR) and RAB5A-expressing MCF10.DCIS.com monolayers is expressed as the mean ± s.d. (> 100 FOV/experimental conditions in *n*= 3 independent experiments). ns>0.999, two-tailed Mann-Whitney non-parametric test.

P values are indicated in each graph.

Supplementary Table 1, Antibodies

REAGENT or RESOURCE	SOURCE	IDENTIFIER	DILUTION	VALIDATION STATEMENTS MANUFACTURERS' WEBSITE
**Antibodies**				
Anti LaminB1	Abcam	Cat# ab16048	1:1000 WB	https://www.abcam.com/lamin-b1-antibody-nuclear-envelope-marker-ab16048.html
Anti LaminA/C (636)	Santa Cruz Biotechnology	Cat# ab7292	1:500 WB	https://www.scbt.com/p/lamin-a-c-antibody-636
Anti STAT2	Thermofisher	Cat# ab44-362G	1:500 WB	https://www.thermofisher.com/antibody/product/STAT2-Antibody-Polyclonal/44-362G
Anti ISG15	Cell Signalling Technology	Cat# ab2743	1:1000 WB	https://www.cellsignal.com/products/primary-antibodies/isg15-antibody/2743
Anti Vinculin	Sigma-Aldrich	Cat# V9131	1:1000 WB	https://www.sigmaaldrich.com/IT/it/product/sigma/v9131
Anti RAB5A	Santa Cruz Biotechnology	Cat# ab309	1:500 WB	NOT more disposable
Anti RAB5A	Abcam	Cat# ab109534	1:100 IHC	https://www.abcam.com/rab5-antibody-epr5438-early-endosome-marker-ab109534.html
Anti p-STAT1 (58D6) (Tyr701)	Cell Signalling Technology	Cat# ab9167	1:1000 WB	https://www.cellsignal.com/products/primary-antibodies/phospho-stat1-tyr701-58d6-rabbit-mab/9167
Anti STAT1 (42H3)	Cell Signalling Technology	Cat# ab9175	1:1000 WB	https://www.cellsignal.com/products/primary-antibodies/stat1-42h3-rabbit-mab/9175
FITC-conjugated Phalloidin	Sigma-Aldrich	Cat# P5282	1:50 IF	https://www.sigmaaldrich.com/IT/it/product/sigma/p5282
Anti IRF3 (D614C)XP	Cell Signalling Technology	Cat# 11904	1:1000 WB	https://www.cellsignal.com/products/primary-antibodies/irf-3-d6i4c-xp-rabbit-mab/11904
Anti STING/TMEM173	Novus Biologicals	Cat# NBP2-24683	1:500 WB	https://www.novusbio.com/products/sting-tmem173-antibody_nbp2-24683
Anti cGAS (D1D3G)	Cell Signalling Technology	Cat # 15102	1:100 IHC (1:1000 WB)	https://www.cellsignal.com/products/primary-antibodies/cgas-d1d3g-rabbit-mab/15102
Anti IFIT1 (D2X9Z)	Cell Signalling Technology	Cat# 14769	1:500 WB	https://www.cellsignal.com/products/primary-antibodies/ifit1-d2x9z-rabbit-mab/14769
Anti Istone H3 trimetil (Lys27)(C36B11)	Cell Signalling Technology	Cat# 9733	1:1000 IF	https://www.cellsignal.com/products/primary-antibodies/tri-methyl-histone-h3-lys27-c36b11-rabbit-mab/9733
Anti fosfo γH2AX S139 (20E3)	Cell Signalling Technology	Cat# 9718	1:500 IHC	https://www.cellsignal.com/products/primary-antibodies/phospho-histone-h2a-x-ser139-20e3-rabbit-mab/9718
Anti-Histone H3 (tri methyl K9) antibody	Abcam	Cat# 8898	3mg for CHIP	https://www.abcam.com/products?keywords=8898
Recombinant Anti-Histone H3 (tri methyl K9) antibody	Abcam	Cat# ab176916	3mg for CHIP	https://www.abcam.com/products?keywords=176916
Anti 53BP1	Abcam	Cat# 175933	1:100 IF	https://www.abcam.com/products?keywords=+175933
Anti p-CHK1 (phospho S345)	Abcam	Cat# ab58567	1:500 IHC	https://www.abcam.com/chk1-phospho-s345-antibody-ab58567.html
Anti α−Tubulin	Sigma-Aldrich	Cat# T5168	1:1000 WB	https://www.sigmaaldrich.com/IT/it/product/sigma/t5168
Anti fosfo γH2AX S139 (20E3)	Abcam	Cat# 11174	1:1000 IHC	https://www.abcam.com/products?keywords=11174
Anti cGAS (clone D1D3G)	Cell Signalling Technology	Cat#15102	1:100 IHC	https://www.cellsignal.com/products/primary-antibodies/cgas-d1d3g-rabbit-mab/15102
Anti SUN2	Abcam	Cat# ab124916	1:100 IF	https://www.abcam.com/products?keywords=124916
Secondary Antibody (Goat Anti-Rabbit Antibody Conjugated to Horseradish Peroxidase)	Cell Signalling Technology	Cat# 7074	1:3000	https://www.cellsignal.com/products/secondary-antibodies/anti-rabbit-igg-hrp-linked-antibody/7074
Secondary Antibody (Goat Anti-Mouse Antibody Conjugated to Horseradish Peroxidase)	Cell Signalling Technology	Cat# 7076	1:3000	https://www.cellsignal.com/products/secondary-antibodies/anti-mouse-igg-hrp-linked-antibody/7076
DAPI	Thermofisher	Cat# D-1306	1:5000	https://www.thermofisher.com/order/catalog/product/D1306?SID=srch-srp-D1306
Hoechst	Thermofisher	Cat# 62249	1: 20	https://www.thermofisher.com/order/catalog/product/62249?SID=srch-srp-62249
IncuCyte® NucLight Rapid Red dye	Sartorius	Cat# 4717	1:1000	https://www.sartorius.com/en/search?search=4717#24598_24596=4717
MitoTraker Red CMXRos	Thermofisher	Cat# M7512	100 nM	https://www.thermofisher.com/order/catalog/product/M7512
Cy3 AffiniPure Donkey Anti-Rabbit IgG (H+L)	Jackson ImmunoResearch	Cat# 711-165-152	1:400	https://www.jacksonimmuno.com/catalog/products/711-165-152
Cy3 AffiniPure Donkey Anti-Mouse IgG (H+L)	Jackson ImmunoResearch	Cat# 715-165-150	1:400	https://www.jacksonimmuno.com/catalog/products/715-165-150
Donkey anti-Rabbit IgG (H+L) Highly Cross-Adsorbed Secondary Antibody, Alexa Fluor 488	Thermofisher	Cat# A32790	1:100	https://www.thermofisher.com/antibody/product/Donkey-anti-Rabbit-IgG-H-L-Highly-Cross-Adsorbed-Secondary-Antibody-Polyclonal/A32790
Donkey anti-Mouse IgG (H+L) Highly Cross-Adsorbed Secondary Antibody, Alexa Fluor 488	Thermofisher	Cat# A21202	1:100	https://www.thermofisher.com/antibody/product/Donkey-anti-Mouse-IgG-H-L-Highly-Cross-Adsorbed-Secondary-Antibody-Polyclonal/A-21202
**Chemicals**				
Etoposide	Selleckchem	Cat# S1225		
Docetaxel	Selleckchem	Cat# S1148		
Doxycycline hyclate	Sigma-Aldrich	Cat# D9891		
SYBR Gold	Thermo-Fisher	Cat# S11494		
RU.521	Selleckchem	Cat# S6841		
H151	Selleckchem	Cat# S6652		
MRT67307	Selleckchem	Cat# S7948		
**Critical Commercial Assays**				
CometAssay® Kit	Trevigen	Cat# 4250-050-K		
RNeasy mini kit	Qiagen	Cat# 74104		
2'3'-cGAMP ELISA Kit	Cayman Chemical	Cat# 501700		
SuperScript VILO cDNA Synthesis Kit	Thermofisher	Cat#11754050		
**Extracellular Matrices**				
Corning® Matrigel® Matrix	Corning	Cat# 354263		
High Concentration (HC),				
Growth Factor Reduced (GFR) *LDEV-free				
**Oligonucleotides**				
siRNAs targeting MB21D1 (cGAS)	Horizon	Cat# L-015607-02-0010		
siRNAs targeting TMEM173 (STING)	Thermofisher	Cat# s226307		
siRNAs targeting IRF3	Thermofisher	Cat# s7507		
siRNAs targeting IRF9	Thermofisher	Cat# s20292		
siRNAs targeting STAT1	Thermofisher	Cat# s277		
siRNAs targeting STAT2	Thermofisher	Cat# s13528		
siRNAs targeting EZH2	Thermofisher	Cat# s4918		
siRNAs targeting SUZ12	Thermofisher	Cat# s23968		
siRNAs targeting TREX1	Horizon	Cat# L-013239-02-0010		
siRNAs targeting YAP	Thermofisher	Cat# s20366		
siRNAs targeting TAZ	Thermofisher	Cat# s13806		
**qRT-PCR assays**				
**Gene name**		**assay ID**		
18s	Thermofisher	Hs99999901_s1		
GAPDH	Thermofisher	Hs99999905_m1		
GUSB	Thermofisher	Hs99999908_m1		
HPRT	Thermofisher	Hs99999909_m1		
RAB5A	Thermofisher	Hs00702360_s1		
IFI27	Thermofisher	Hs01086373_g1		
IFI44	Thermofisher	Hs00197427_m1		
IFI44L	Thermofisher	Hs00915292_m1		
IFI6	Thermofisher	Hs00242571_m1		
IFIT1	Thermofisher	Hs03027069_s1		
IFIT3	Thermofisher	Hs01922752_s1		
ISG15	Thermofisher	Hs01921425_s1		
MX2	Thermofisher	Hs01550814_m1		
OASL	Thermofisher	Hs00984387_m1		
MB21D1 (cGAS)	Thermofisher	Hs00403553_m1		
TMEM173 (STING)	Thermofisher	Hs00736958_m1		
IRF3	Thermofisher	Hs01547277_m1		
IRF9	Thermofisher	Hs00196051_m1		
STAT1	Thermofisher	Hs01013996_m1		
STAT2	Thermofisher	Hs01013115_g1		
LMNA	Thermofisher	Hs00153462_m1		
LMNB1	Thermofisher	Hs01059210_m1		
EZH2	Thermofisher	Hs01016789_m1		
SUZ12	Thermofisher	Hs00248742_m1		
CDH1	Thermofisher	Hs00170423_m1		
AXIN2	Thermofisher	Hs00610344_m1		
CDH2	Thermofisher	Hs00169953_m1		
EGF	Thermofisher	Hs01099999_m1		
MMP13	Thermofisher	Hs00942584_m1		
ZEB1	Thermofisher	Hs00232783_m1		
CTGF	Thermofisher	Hs00170014_m1		
CYR61	Thermofisher	Hs00998500_g1		
ANKRD1	Thermofisher	Hs00923602_g1		
TREX1	Thermofisher	Hs03989617_s1		
YAP	Thermofisher	Hs00902712_g1		
TAZ	Thermofisher	Hs00179826_m1		
Gapdh	Thermofisher	Mm99999915_g1		
Gusb	Thermofisher	Mm01197698_m1		
Hprt1	Thermofisher	Mm00446968_m1		
Cxcl10	Thermofisher	Mm00445235_m1		
Ifi35	Thermofisher	Mm01260550_g1		
Ifit1	Thermofisher	Mm00515153_m1		
Irf7	Thermofisher	Mm00516793_g1		
Irf9	Thermofisher	Mm00492679_m1		
Isg15	Thermofisher	Mm01705338_s1		
Oas3	Thermofisher	Mm00460944_m1		
Stat1	Thermofisher	Mm00439531_m1		
Stat2	Thermofisher	Mm00490880_m1		
Rab5a	Thermofisher	Mm01278246_m1		

Supplementary Table 2, Reagents, Oligos and QRT-PCR assays

REAGENT or RESOURCE	SOURCE	IDENTIFIER
**Chemicals**		
Etoposide	Selleckchem	Cat# S1225
Docetaxel	Selleckchem	Cat# S1148
Doxycycline hyclate	Sigma-Aldrich	Cat# D9891
SYBR Gold	Thermo-Fisher	Cat# S11494
RU.521	Selleckchem	Cat# S6841
H151	Selleckchem	Cat# S6652
MRT67307	Selleckchem	Cat# S7948
**Critical Commercial Assays**		
CometAssay® Kit	Trevigen	Cat# 4250-050-K
RNeasy mini kit	Qiagen	Cat# 74104
2'3'-cGAMP ELISA Kit	Cayman Chemical	Cat# 501700
SuperScript VILO cDNA Synthesis Kit	Thermofisher	Cat#11754050
**Extracellular Matrices**		
Corning® Matrigel® Matrix	Corning	Cat# 354263
High Concentration (HC),		
Growth Factor Reduced (GFR) *LDEV-free		
**Oligonucleotides**		
siRNAs targeting MB21D1 (cGAS)	Horizon	Cat# L-015607-02-0010
siRNAs targeting TMEM173 (STING)	Thermofisher	Cat# s226307
siRNAs targeting IRF3	Thermofisher	Cat# s7507
siRNAs targeting IRF9	Thermofisher	Cat# s20292
siRNAs targeting STAT1	Thermofisher	Cat# s277
siRNAs targeting STAT2	Thermofisher	Cat# s13528
siRNAs targeting EZH2	Thermofisher	Cat# s4918
siRNAs targeting SUZ12	Thermofisher	Cat# s23968
siRNAs targeting TREX1	Horizon	Cat# L-013239-02-0010
siRNAs targeting YAP	Thermofisher	Cat# s20366
siRNAs targeting TAZ	Thermofisher	Cat# s13806
**qRT-PCR assays**		
**Gene name**		**assay ID**
18s	Thermofisher	Hs99999901_s1
GAPDH	Thermofisher	Hs99999905_m1
GUSB	Thermofisher	Hs99999908_m1
HPRT	Thermofisher	Hs99999909_m1
RAB5A	Thermofisher	Hs00702360_s1
IFI27	Thermofisher	Hs01086373_g1
IFI44	Thermofisher	Hs00197427_m1
IFI44L	Thermofisher	Hs00915292_m1
IFI6	Thermofisher	Hs00242571_m1
IFIT1	Thermofisher	Hs03027069_s1
IFIT3	Thermofisher	Hs01922752_s1
ISG15	Thermofisher	Hs01921425_s1
MX2	Thermofisher	Hs01550814_m1
OASL	Thermofisher	Hs00984387_m1
MB21D1 (cGAS)	Thermofisher	Hs00403553_m1
TMEM173 (STING)	Thermofisher	Hs00736958_m1
IRF3	Thermofisher	Hs01547277_m1
IRF9	Thermofisher	Hs00196051_m1
STAT1	Thermofisher	Hs01013996_m1
STAT2	Thermofisher	Hs01013115_g1
LMNA	Thermofisher	Hs00153462_m1
LMNB1	Thermofisher	Hs01059210_m1
EZH2	Thermofisher	Hs01016789_m1
SUZ12	Thermofisher	Hs00248742_m1
CDH1	Thermofisher	Hs00170423_m1
AXIN2	Thermofisher	Hs00610344_m1
CDH2	Thermofisher	Hs00169953_m1
EGF	Thermofisher	Hs01099999_m1
MMP13	Thermofisher	Hs00942584_m1
ZEB1	Thermofisher	Hs00232783_m1
CTGF	Thermofisher	Hs00170014_m1
CYR61	Thermofisher	Hs00998500_g1
ANKRD1	Thermofisher	Hs00923602_g1
TREX1	Thermofisher	Hs03989617_s1
YAP	Thermofisher	Hs00902712_g1
TAZ	Thermofisher	Hs00179826_m1
Gapdh	Thermofisher	Mm99999915_g1
Gusb	Thermofisher	Mm01197698_m1
Hprt1	Thermofisher	Mm00446968_m1
Cxcl10	Thermofisher	Mm00445235_m1
Ifi35	Thermofisher	Mm01260550_g1
Ifit1	Thermofisher	Mm00515153_m1
Irf7	Thermofisher	Mm00516793_g1
Irf9	Thermofisher	Mm00492679_m1
Isg15	Thermofisher	Mm01705338_s1
Oas3	Thermofisher	Mm00460944_m1
Stat1	Thermofisher	Mm00439531_m1
Stat2	Thermofisher	Mm00490880_m1
Rab5a	Thermofisher	Mm01278246_m1

Supplementary Table 3

Organoids were prepared as described[2]. The kinematic and dynamical analysis of the whole organoid and their labelled nuclei was performed as described[3].

Sample N.	TYPE	TISSUE TYPE	DIAGNOSIS	ER (%)	PGR (%)	HER2 SCORE	HER2 %	KI67 (%)	AGE
BR 25	Pathological	Breast - Left	Infiltrating duct carcinoma	60	60	Neg		4	49
BR 30	Pathological	Breast - Right	Infiltrating duct carcinoma	90	90	3+	90	28	46
BR 39	Pathological	Breast - Left	Infiltrating duct carcinoma	95	95	Neg		12	50
BR 40	Pathological	Breast - Right	Cribriform carcinoma	95	30	1+	15	3	56
BR 44	Pathological	Breast - Right	Infiltrating duct carcinoma	95	40	Neg		22	43

Supplementary Note: Discussion

Like inert soft materials, such as foams and colloids, normal and malignant epithelial tissues can undergo a solid-to-fluid phase transition, which enables them to flow and their constituents to rearrange, while maintaining overall mechanical integrity. This seemingly purely physical process has potential remarkable relevance in clinical cell biology: during carcinogenesis, for example, normal mammary epithelia frequently evolve into solid, rigid, and jammed masses that are densely packed with cancer cells, generating a ductal carcinoma in situ (DCIS); to become invasive ductal carcinoma (IDC), the malignant tissue must gain a certain degree of fluidity to be able to proliferate, migrate collectively and disseminate locally. In this work, we show that this material-like solid-to-fluid phase transition is a rapid, short-term adaptive response to mechanical challenging conditions that, in addition to promoting collective dissemination of early lesions, also results in a long term, transcriptional-dependent phenotype switch toward a cGAS/STING-mediated inflammatory gene response. Remarkably even this long-lived transcriptional adaptation is the result of a response to mechanically challenged conditions to which tissue that transit from a solid, jammed and immobile to a fluid, motile state is subjected. We showed that fluidifictaion via flocking is invariably accompanied and possibly caused by by large density fluctuations and cell deformations that are immediately transferred to cell nuclei. Cell reacts to these stresses by mounting several nuclear mechano-protective strategies (such nuclear rigidification, or perinuclear actin cages) that eventually fail, resulting in frequent nuclear envelope ruptures and the release of chromatinized DNA: a potent cGAS/STING mediated proinflammatory, innate immunity response that drastically rewires the transcriptional state of carcinoma collectives.

Previous studies showed that cancer cell invasion into the confinement of narrow channels and gaps of interstitial tissues exerts large mechanical strains on the nucleus that, when above a certain threshold, can lead to nuclear envelope ruptures (NER) and transient leakage of DNA into the cytosol[4-7]. These events, however, are typically short-lived as NER are efficiently and rapidly repaired by the Endosomal Sorting Complex Required for Transport (ESCORT III) system[4, 8] and the Barrier-to-autointegration factor (BAF)[9], and have not been shown to promote permanent cell fate changes or transcriptional rewiring. BAF, specifically, can also inhibit cGAS binding to DNA and cGAS activation[10]. Tissue fluidification via flocking results, however, in a persistent and a long-lived, chronic mechanical stress state. Tumor cells react to these challenges by mounting a set of diverse mechanoprotective response strategies[11], including nuclear stiffening, alterations in the distribution and structures of heterochromatin, loss of Lamin B1, and the formation of persistent and nuclear protective actin rings[11]. However, as mechanical strains driven by large-scale tissue fluctuations and small-scale nuclear deformations persist in fluidized tissues, nuclear mechanoprotection eventually fails. This results in frequent and repeated NER, and in mechanically-induced, aberrantly elevated DNA damage. These combined events might lead to the accumulation of fragmented DNA that overcome the inhibitory action of BAF and potently activates sustained cGAS/STING signaling, leading to rewiring of the transcriptional profile of DCIS toward an innate immunity, interferon-like, inflammatory response. Remarkably, such changes are a cell-intrinsic and an emerging property of tumor cell collectives, rather than dependent on the interaction of the malignant tissue with the microenvironment. It must be noted, however,

that mechanical perturbations associated with tissue fluidification via unjamming have recently been shown to be driven also by extreme ECM-mediated confinement during invasive growth of triple-negative mammary cancer[12] or by compressive stresses[13]. These microenvironmental-driven alterations might also contribute to the activation of proinflammatory, transcriptional changes. Indeed, we showed that in naturally occurring DCIS the activation of cGAS and the increased elevation of γH2AX frequently accompany the formation of infiltrative foci that mark the transition toward a more invasive phenotype. At these sites, like in model DCIS in 2D and 3D, and in living breast cancer organoids, invasive buds display a graded elevation of RAB5A expression, associated with persistent angular motility and local gradients of fluidification. These local alterations in tissue dynamics are expected to increase chronic mechanical stress, thereby instigating conditions that facilitate NER and cGAS/STING elevation. Recently, we have also shown that extreme confinement in DCIS cancer model cells might be sufficient to promote NERs[14]. These events, however, are relatively transient, but sufficient to promote the nuclear entry of exonucleases, such as TREX1. TREX1 nuclear re-localization, in turn, critically contributes to damage the DNA, under conditions in which the leakage of DNA into the cytoplasm is limited and cGAS is not activated[14].

Recently, skin epidermis stem/progenitor cells subjected to relatively short cycles of stretching have been shown to trigger amplitude-dependent supracellular and nuclear mechanoresponses[11]. These include the formation of transient actin rings and a nuclear tension-mediated, Ca^{++} dependent reduction in the levels of H3K9me3 heterochromatin, resulting in chromatin fluidification and nuclear softening to dissipate mechanical energy[11]. Sustained chromatin fluidification, in turn, led to transcriptional repression and decreased expression of cell identity and differentiation genes[11]. Unjamming via flocking imposes, however, qualitative, and quantitative different mechanical strains, that, nevertheless, elicit a robust set of mechanoprotective responses. These protective strategies, like in skin epidermis stem/progenitor cells, include the permanent formation of actin rings, but no alterations in the global constitutive levels of H3K9me3 heterochromatin, which is, nevertheless, architecturally reorganized. In addition, we detected a slight global increase and a robust redistribution at the nuclear periphery of the facultative H3K27me3 marks. Notably, this latter modification has been shown to occur upon long-term (6-24 hours) stretching, and to be responsible for increase nuclear rigidity[11, 15], similarly to what we found in fluidized normal and oncogenic mammary epithelia. Persistency of these alterations in the presence of constant mechanical stress exerted on cells and nuclei results in NER, leakage of cytoplasmic DNA, and DNA damage. Thus, cells mount distinctive mechanoprotective responses that are not only dependent on the time and extent of mechanical perturbations but are likely cell context related.

Supplementary Methods

Cell cultures and transfection

MCF10.DCIS.com cells were provided by J. F. Marshall (Barts Cancer Institute, Queen Mary University of London, UK) and maintained in DMEM/F12 (Biowest) supplemented with 5% horse serum (Life Technologies), 2 mM L-Glutamine (EuroClone), 0.5 mg/ml Hydrocortisone (Sigma-Aldrich), 10 µg/ml Human insulin (Sigma-Aldrich) and 20 ng/ml EGF (Peprotech).

MCF10A cells were a gift from J. S. Brugge (Department of Cell Biology, Harvard Medical School, Boston, USA) and were maintained in DMEM/F12 (Biowest) supplemented with 5% horse serum (Life Technologies), 2 mM L-Glutamine (EuroClone), 0.5 mg/ml Hydrocortisone (Sigma-Aldrich), 100 ng/ml cholera toxin (Sigma-Aldrich), 10 µg/ml Human insulin (Sigma-Aldrich) and 20 ng/ml EGF (Peprotech).

HaCaT (ATCC) were maintained DMEM (Lonza) + 10% FBS (Life Technologies) + 2 mM L-Glutamine (EuroClone).

Phoenix-AMPHO cells (American Type Culture Collection, CRL-3213) were used as the packaging cell line for the generation of retroviral particles and cultured as recommended by the supplier.

HEK293T (BBCF-Biological Bank and Cell factory, INT, Milan) were grown in DMEM (Lonza) supplemented with 10% FBS (Life Technologies) and 2 mM L-Glutamine (EuroClone) and used as the packaging line for lentiviral vectors.

MCF10A cells were infected with pSLIK-neo-EV (empty vector, CTR) or pSLIK-neo-RAB5A lentiviruses and selected with the appropriate antibiotic to obtain stable inducible cell lines. MCF10.DCIS.com were infected with pSLIK-neo-EV (empty vector, CTR) or pSLIK-neo-RAB5A lentiviruses and selected with the appropriate antibiotic to obtain stable inducible cell lines. Constitutive expression of mCherry H2B was achieved by retroviruses infection of MCF10DCIS.com cells with pBABE-puro-mCherry-H2B vector. pLL5.0 E-Cadherin shRNA/mEcad-GFP vector was a gift from Alpha S. Yap (Division of Molecular Cell Biology, Institute for Molecular Bioscience, The University of Queensland, Australia). pTRIP-CMV-GFP-FLAG-cGAS GFP vector was from Addgene (plasmid# 86675). pTRIP-SFFV-EGFP-NLS vector was from Addgene (plasmid# 86677). EGFP-Nesprin1 is a mini-Nesprin1 cloned into a pCDH-EGFP plasmid, built using the N-terminal region of giant Nesprin1 (1521bp, aa 1-507), corresponding to the actin-binding Calponin domain, and the C-terminus (1422bp, aa 8325-8797) corresponding to the KASH domain.

Transfections were performed using either calcium phosphate or FuGENE HD Transfection Reagent (Promega, Cat# E2311), according to the manufacturer's instructions.

All cell lines have been authenticated by cell fingerprinting and tested for mycoplasma contamination. Cells were grown at 37 °C in a humidified atmosphere with 5% CO_2.

Generation of lentiviral and retroviral particles

Packaging of lentiviruses or retroviruses was performed following standard protocols[16].

RNA interference

siRNA delivery was achieved by mixing 50 nM of specific siRNAs with Optimem and Lipofectamine RNAiMAX Transfection Reagent (Thermofisher, Cat# 13778150). The first

cycle of interference (reverse transfection) was performed on cells in suspension. The day after, a second cycle of interference (forward transfection) was performed on cells in adhesion. The siRNAs used for knocking down specific genes are reported reagents' table. For each RNA interference experiment, a negative control was performed with the same amounts of scrambled siRNAs. Silencing efficiency was controlled by qRT-PCR.

Quantitative RT-PCR analysis and Immunoblotting

Quantitative RT-PCR analysis was performed as previously described[3].

Tissue collection

Breast biopsies were collected from women undergoing mastectomy for primary breast cancer. Donors were informed before the surgery and agreed by written consent to donate tissues. The use of human material has been reviewed by European Institute of Oncology Ethical Committees (reference to UID 2152). The permit for obtaining clinical material did not include access to basic information regarding patients and their detailed medical histories are not given to the authors. Tumor biopsies were processed immediately upon receipt and cultured as described below.

Immunohistochemistry on DCIS and IDC

Immunolocalization analysis of mouse and human tissues sections

Four-micrometers-thick human and mouse tissue sections were deparaffinized, rehydrated and unmasked using Novocastra Epitope Retrieval Solutions pH6 or pH9 in thermostatic bath at 98°C for 30 minutes. Subsequently, the sections were brought to room temperature and washed in PBS. After neutralization of the endogenous peroxidase with 3% H_2O_2 and Fc blocking by a specific protein block (Leica Novocastra), the samples were incubated with the following primary antibodies: RAB5A (clone EPR5438, 1:100 pH6, ab109534, Abcam), phospho-γH2AX (1:1000 pH6, ab11174, Abcam), cGAS (clone D1D3G, 1:100 pH6, #15102, Cell Signaling), pCHK1 (1:500 pH9, ab58567, Abcam).
IHC staining for cGAS was revealed using Novolink Polymer Detection Systems (Leica Novocastra) and DAB (3,3'-Diaminobenzidine, Leica Novocastra) as substrate chromogen and the slides were counterstained with Harris hematoxylin (Novocastra).
For multiple-marker immunostainings, to multiplex antibodies raised in the same species, Opal Multiplex IHC kit was developed. After deparaffinization, antigen retrieval in pH6 buffer was brought to a boil at 100% power, followed by 20% power for 15 minutes using microwave technology (MWT). Sections were treated with blocking buffer for 10 minutes at room temperature before primary antibody incubation. Slides were then incubated with Polymeric horseradish peroxidase-conjugated (HRP) secondary antibody for 10 minutes and the signal was visualized using Opal 520 fluorophore-conjugated tyramide signal amplification (TSA) at 1:100 dilution. The HRP catalyze covalent deposition of fluorophores around the marker of interest. The slides were again processed with the microwave treatment to strip primary/secondary antibody complex and allow the next antigen-antibody staining. Another round of staining was performed with the second primary antibody incubation, followed by Polymeric horseradish peroxidase-conjugated (HRP) secondary antibody and Opal 620 fluorophore-conjugated tyramide signal amplification (TSA) at 1:100 dilution for signal visualization. Finally, slides were again microwaved in antigen retrieval buffer and nuclei were subsequently visualized with DAPI

(4’,6-diamidin-2-fenilindolo). All slides were analyzed under a Zeiss Axioscope A1 microscope equipped with four fluorescence channels widefield IF. Microphotographs were collected using a Zeiss Axiocam 503 Color digital camera with the Zen 2.0 Software (Zeiss).

Quantification of γH2AX in human primary tumor samples

We used a semi-automated image analysis pipeline to quantify the location of the γH2AX expressing cells in the tumoral ductal adenocarcinoma regions. Specifically, we employed a deep learning based nuclear segmentation (Stardist)[1] to segment individual nuclei and identified the centroid positions of each nuclei in the image frame. A semi-automated analysis was used to identify the location of whole area, the core region and the outer margin of the ductal adenocarcinoma. The proper assignment of these regions was verified by a trained pathologist. In all cases, we removed the segmented nuclei with a centroid 20 pixels from the border regions of the imaging frame. We used an automated histogram-based thresholding in each frame to identify regions with high expression of γH2AX signal, above the threshold applied. We also removed isolated small spots as noise. Next, we identified the nuclei that display either a positive or weak/absent (below the arbitrary established threshold levels) γH2AX signal. For each case, we quantified the total number of nuclei, the number of nuclei with strongly positive or weak/no γH2AX signal in the tumor core, tumor surface and in the whole tumoral areas and computed the percentage of nuclei with strongly positive γH2AX signal in the core region, tumor front regions and in the whole tumor region over 9 independent cases. The whole image analysis pipeline is presented in Video13.

For the quantification of the percentage of RAB5A-positive displaying γH2AX positive or cGAS positive signals. After semiautomated identification the whole area, the core region and the outer margin of the ductal-adeno-carcinomas, as described above, the percentage of cells in the various areas expressing express high (>2 on scale from 0.1,2,3) or low l (< 2 on scale from 0.1,2,3) levels of RAB5A identified by expert pathologists and positive for either γH2AX positive or cGAS was counted.

Transcriptomics analyses

Control and RAB5A-expressing monolayers MCF10A or MCF10.DCIS.com cells were seeded in six-well plates (1.5×10^6 cells per well) and cultured until a uniform monolayer had formed. Three independent biological replicas were performed for all this analysis. RAB5A expression was induced, by adding fresh complete media supplemented with 2.5 µg/ml doxycycline hyclate to cells. Comparable cell confluence was tested by taking pictures by differential interference contrast imaging using a 10× objective and counting the number of nuclei per field. After 48 h monolayers were processed for RNA extraction using TRIzol reagent (Thermo Fisher) and processed for total RNA extraction with PureLink™ RNA Mini Kit (Thermo Fisher), according to manufacturer’s instructions.

For RNA-seq analysis of breast cancer patients derived organoids, matrigel droplets containing organoids were directly lysed as described above. The RNA quality was assessed by the RNA Integrity Number (RIN) value with RNA 6000 Nano kit assay (Agilent). Only samples with RIN > 8.0 were used in this study. RNA-seq libraries were constructed according to the TruSeq mRNA Stranded preparation kit (Illumina, San Diego, USA) and sequenced at HiSeq2500.

Libraries for mRNA-Seq: mRNA-seq indexed library preparation was performed starting from 500 ng of total mRNA (Illumina, TruSeq Stranded mRNA, 20020594) according to the manufacturer's instructions. Indexed libraries were quality controlled on Agilent Bioanalyzer 2100 (High Sensitivity DNA kit), quantified (Qubit dsDNA HS Assay, Q32851), normalized and pooled to perform a multiplexed sequencing run. 1% PhiX control was added to the sequencing pool, to serve as a positive run control. Sequencing was performed in PE mode (2x75nt) on an Illumina NextSeq550 platform, generating on average 50 million PE reads per sample. Experiment was performed using biological triplicates; a total of 12 samples were sequenced.

Reads were aligned to the GRCh38/hg38 assembly human reference genome using the STAR aligner[17] with default settings with the parameter --quantMode GeneCounts in order to obtain gene counts. Differential gene expression analysis was performed using the Bioconductor package DESeq2[18] that estimates variance-mean dependence in count data from high-throughput sequencing data and tests for differential expression exploiting a negative binomial distribution-based model. Preranked gene set enrichment analysis (GSEA) for evaluating pathway enrichment in transcriptional data was carried out using the Bioconductor package fgsea[19], taking advantage of the Hallmarks, KEGG and chemical and genetic perturbations (CGP) gene sets available from the GSEA Molecular Signatures Database (http://www.gsea-msigdb.org/gsea/msigdb/collections.jsp).

Transcription factor enrichment analysis for overlap between the input set of differentially expressed genes and entries of the ChEA and ENCODE databases was performed using the Transcription Factor Enrichment Analysis tool from the X2K Web suite (https://maayanlab.cloud/X2K/) that infers upstream regulatory networks from lists of differentially expressed genes.

Chip-Seq experiments

Control or RAB5A-expressing MCF10 DCIS.com monolayers were treated with Formaldheyde (F8775 SIGMA) in a PBS-solution (final 1%) for 10 min while rocking at room temperature and quenched with 0.125 M Glycine for 5 min. Cells were washed twice with cold PBS and scraped off the plates. PBS-washed cells were transferred to 15 ml tubes and spun down at 400g at 4C. The pellet was lysed with 1X sonication lysis buffer (10 mM Tris pH 8.0, 0.25% SDS, 2 mM EDTA, plus protease inhibitors) and incubated for at least 10 min at 4°C. Lysed chromatin was sheared at the average size of 300 bp fragments using Covaris® E220 evolution ultrasonicator (settings: duty factor 20%, peak incidence power 75 Watt, cycles per burst 200, 5 minutes). Sonicated chromatin (3 mg) was incubated overnight at 4°C with 3 mg of the following histone mark antibodies: H3K9me3 ab176916, H3K9me3 ab8898. Immunocomplexes were recovered with 20 µl of pre-blocked Protein G-Dynabeads (Thermo Fisher) for 2 h, at 4°C, and washed twice with RIPA-low salt, twice with RIPA-high salt, twice with RIPA-LiCl and once with 10 mM Tris pH 8.0 and once with 1X TE, as previously reported[20]. The washed immunocomplexes were incubated with ChIP elution buffer (10 mM Tris-HCl pH 8.0, 5 mM EDTA pH 8.0, 300 mM NaCl, 0.4% SDS) supplemented with 0.8 mg/ml Proteinase K for 1 h at 55°C and overnight at 65°C, for reverse crosslinking. The immunoprecipitated DNA was then purified by Qiagen MinElute kit (Qiagen) and eluted in 22 µl EB buffer. ChIP-seq libraries were constructed with TruSeq ChIP Library Preparation Kit (Illumina),

according to the manufacturer's instructions and sequenced on Illumina HiSeq2500 platform.
Libraries for ChIP-Seq: 2 ng of DNA having fragment size 350-400bp were used to synthfhesize libraries for Chromatin Immunoprecipitation profiles (Kapa HyperPrep kit; Roche KK8504, KK8727). Indexed DNA libraries were size-selected and purified (AmpureXP, Beckman, A63881), quantitated (Qubit dsDNA HS Assay, Q32851), checked for size distribution on Agilent Bioanalyzer 2100 (DNA HS kit, Agilent, 5067-4626) and normalized for pooling. 1% PhiX control was added to the sequencing pool, to serve as a positive run control. Sequencing was performed in SR mode (1x75nt) on an Illumina NextSeq550 platform, generating on average 35 million SR reads per sample. A total of 14 samples were sequenced

SAMMY-seq experiments.
Three distinct biological replicas of control and RAB5A expressing MCF10.DCIS.com monolayers were processed for chromatin fractionation as described[21], with minor adaptions. Briefly, Three distinct biological replicas of control and RAB5A expressing MCF10.DCIS.com monolayers were processed for chromatin fractionation as described[21], with minor adaptions. Briefly, 3 million cells were washed in PBS 1X, and extracted in 600 μl of cytoskeleton buffer (CSK: 10 mM PIPES pH 6,8; 100 mM NaCl; 1 mM EGTA; 300 mM Sucrose; 3 mM MgCl2; 1X protease Inhibitors by Roche Diagnostics; 1 mM PMSF) supplemented with 1 mM DTT and 0,5% Triton X-100. After 10 min on wheel at 4°C the cytoskeletal structure was separated from soluble proteins by centrifugation at 900g for 3 min at 4°C, and the supernatant was labeled as S1 fraction. The pellets were resuspended with 600 μl of cytoskeleton buffer, put 10 min on wheel at 4°C followed by centrifugation at 900g for 3 min at 4°C. Chromatin was solubilized by DNA digestion with 25U of RNase–free DNase (Turbo DNAse; Invitrogen AM2238) in 100 μl of CSK buffer for 60 min at 37°C. To stop digestion, ammonium sulphate was added in CSK buffer to a final concentration of 250 mM and, after 5 min in ice, samples were pelleted at 2350g for 3 min at 4°C and the supernatant was labeled as S2 fraction. The pellets were resuspended with 200 μl of CSK buffer, put 10 min on wheel at 4°C followed by centrifugation at 2350g for 3 min at 4°C. The pellet was further extracted with 100 μl of CSK buffer with 2M NaCl for 5 min at 4°C, centrifuged at 2350g 3 min at 4°C and the supernatant was labeled as S3 fraction. This treatment removed the majority of histones from chromatin. The pellets were washed twice with 200 μl of CSK buffer with 2M NaCl, put 10 min on wheel at 4°C followed by centrifugation at 2350g for 3 min at 4°C. The pellets were solubilized in 100 μl of 8M urea buffer for 10 min at room temperature to remove any remaining protein component by applying highly denaturing conditions. This fraction was labeled as S4. DNA was extracted from S2, S3 and S4 fractions.

Libraries for SAMMY-Seq: for fractions S2, S3 and S4 obtained from chromatin fractionation procedure, at least 2.5 ng DNA were used to generate an indexed library (Kapa HyperPrep kit; Roche KK8504, KK8727). Indexed DNA libraries were purified (AmpureXP, Beckman, A63881), quantitated (Qubit dsDNA HS Assay, Q32851), checked for size distribution on Agilent Bioanalyzer 2100 (DNA HS kit, Agilent, 5067-4626) and normalized for pooling. 1% PhiX control was added to the sequencing pool, to serve as a positive run control. Sequencing was performed in SR mode (1x75nt) on an Illumina

NextSeq550 platform, generating at least 30 million SR reads per sample. Experiment was performed using biological triplicates; A total of 18 samples were sequenced

SAMMY-seq and ChIP-seq data analysis

Preprocessing of sequencing reads

Sequencing reads were trimmed and adapters removed by using Trimmomatic (v0.39)[22] using the following parameters for SAMMY-seq and ChIP-seq data: 2 for seed_mismatch, 30 for palindrome_threshold, 10 for simple_threshold, 3 for leading, 3 for trailing and 4:15 for sliding window and sequence minimum length threshold of 35. As clip file has been used the trimmomatic provided dataset “TruSeq3-SE.fa” (for single end). In addition, only for SAMMY-seq data, all reads were cropped to 75 bp reads length (if longer) by setting the crop option of Trimmomatic (v0.39) to 75. After trimming, the reads were aligned using BWA (v0.7.17-r1188)[23] etting –k parameter as 2 and using as reference genome the UCSC hg38 one (only canonical chromosomes have been taken into consideration). The alignment duplicates have been marked with Picard (v2.22) (http://broadinstitute.github.io/picard/) MarkDuplicates option. And then filtered using Samtools (v1.9)[24], in addition we filtered all the reads with mapping quality lower than 1, unmapped and read fails platform/vendor quality checks (-F 1540 -q 1).
Each sequencing lane has been analysed separately up to this point and then merged.

Genomic tracks for data visualization

The comparison between sequencing reads enrichment in ChIP-seq (ChIP over input control reads enrichment) or SAMMY-seq fractions comparison was performed using the SPP (v1.16.0)[25] (v3.5.2) library. The reads have been imported from the (previously filtered) bam files using the “read.bam.tags” function, then they were filtered using “remove.local.tag.anomalies” and finally the normalized log2 reads density ratio was computed using the function “get.smoothed.enrichment.mle” setting “tag.shift = 0” and “background.density.scaling = TRUE” to exclude enriched regions from the calculation of normalization scaling factor.
The comparisons between sequencing reads enrichment were graphically plot using the R (v3.5.2) library Gviz (1.26.5) setting 1000 as number of represented points (“window” parameter).

Heterochromatin domains calling

Heterochromatin (H3K9me3 enriched) domains were defined using the EDD (v1.1.19)[26] software with parameters (binsize = 200 Kb and gap penalty = 25) processing the filtered bam files obtained as described above. The “required_fraction_of_informative_bins” parameter was set to 0.98. The unalignable regions were defined with the ENCODE Unified GRCh38 Exclusion List (previously "blacklist") and downloaded from (https://www.encodeproject.org/files/ENCFF356LFX/).

Metaprofile analysis

The metaprofile analysis was performed using DeepTools (v3.4.3)[27]. The metaprofile matrix was calculated using the “computeMatrix” command of DeepTools, using as regions of interest the heterochromatin domains obtained from CTR H3K9me3 ChIP-seq experiment (with antibody ab-176916-Abcam), as described above, and as signal the S4/S2 or the S4/S3 SAMMY-seq fractions comparisons (log2 ratios) calculated with SPP,

as described above. The domains flanking regions (upstream and downstream) were defined of a size of 1 Mb and 20 Kb was chosen as bin size for each bin representing the domain ("binSize" parameter) and the target size of the domains rescaling was defined as 3 Mb. In addition, the "skipZeros" option was added to remove regions with zero reads coverage. The metaprofile matrix was represented using the "plotProfile" tool of DeepTools using as input the previously created matrix.

Immunostaining

Cells were washed twice with 1X PBS, fixed in 4% paraformaldehyde for 10 min and permeabilized with 0.1% Triton X-100 and 10% FBS for 10 min. After a 1X PBS wash, primary antibodies were added for 2 h at room temperature. Coverslips were washed in 1X PBS before secondary antibody incubation for 1 h at room temperature, protected from light. FITC-phalloidin was added in the secondary antibody step, where applicable. After removal of not specifically bound antibodies by 1X PBS washing, nuclei were stained with 0.5 ng ml–1 DAPI. Samples were post-fixed and mounted on glass slides in anti-fade mounting medium (glycerol). Antibodies were diluted in 1X PBS and 10% FBS.

Electron microscopy

Electron microscopic examination, immune EM goldlabelling based on pre embedding, EM tomography and correlative light-electron microscopy (CLEM) were performed as previously described[28-30].

EM Tomography: Two-step CLEM based on the analysis of tomographic reconstructions acquired under low magnification with consecutive reacquisition of EM tomo box under high magnification and its re-examination was used exactly as described[29]. Briefly, an ultramicrotome (Leica EM UC7; Leica Microsystems, Vienna) was used to cut 200 nm serial semi-thick sections. Sections were collected onto 1 % Formvar films adhered to slot grids. Both sides of the grids were labelled with fiduciary 10 nm gold (PAG10, CMC, Utrecht, the Netherlands). Tilt-series were collected from the samples from ± 65° with 1° increments at 200 kV in Tecnai 20 electron microscopes (FEI, Thermo Fisher Scientific, Eindhoven, the Netherlands). Tilt series were recorded at a magnification of 9,600x, using software supplied with the instrument. The nominal resolution in our tomograms was 4 nm, based upon section thickness, the number of tilts, tilt increments, and tilt angle range. The IMOD package and its newest viewer, 3DMOD 4.0.11, were used to construct individual tomograms and for the assignment of the outer leaflet of organelle membrane contours, CLEM was performed exactly as described[28].

References

1. Schmidt, U., Weigert, M., Broaddus, C. & Myers, G. 265-273 (Springer International Publishing, Cham; 2018).
2. Sachs, N. *et al.* A Living Biobank of Breast Cancer Organoids Captures Disease Heterogeneity. *Cell* **172**, 373-386 e310 (2018).
3. Palamidessi, A. *et al.* Unjamming overcomes kinetic and proliferation arrest in terminally differentiated cells and promotes collective motility of carcinoma. *Nat Mater* **18**, 1252-1263 (2019).
4. Raab, M. *et al.* ESCRT III repairs nuclear envelope ruptures during cell migration to limit DNA damage and cell death. *Science* **352**, 359-362 (2016).
5. Denais, C.M. *et al.* Nuclear envelope rupture and repair during cancer cell migration. *Science* **352**, 353-358 (2016).
6. Pfeifer, C.R. *et al.* Constricted migration increases DNA damage and independently represses cell cycle. *Mol Biol Cell* **29**, 1948-1962 (2018).
7. Xia, Y. *et al.* Nuclear rupture at sites of high curvature compromises retention of DNA repair factors. *J Cell Biol* **217**, 3796-3808 (2018).
8. Radulovic, M. & Stenmark, H. ESCRTs in membrane sealing. *Biochem Soc Trans* **46**, 773-778 (2018).
9. Halfmann, C.T. *et al.* Repair of nuclear ruptures requires barrier-to-autointegration factor. *J Cell Biol* **218**, 2136-2149 (2019).
10. Guey, B. *et al.* BAF restricts cGAS on nuclear DNA to prevent innate immune activation. *Science* **369**, 823-828 (2020).
11. Nava, M.M. *et al.* Heterochromatin-Driven Nuclear Softening Protects the Genome against Mechanical Stress-Induced Damage. *Cell* **181**, 800-817 e822 (2020).
12. Ilina, O. *et al.* Cell-cell adhesion and 3D matrix confinement determine jamming transitions in breast cancer invasion. *Nat Cell Biol* **22**, 1103-1115 (2020).
13. Kilic, A. *et al.* Mechanical forces induce an asthma gene signature in healthy airway epithelial cells. *Sci Rep* **10**, 966 (2020).
14. Nader, G.P.F. *et al.* Compromised nuclear envelope integrity drives tumor cell invasion. *bioRxiv*, 2020.2005.2022.110122 (2020).
15. Le, H.Q. *et al.* Mechanical regulation of transcription controls Polycomb-mediated gene silencing during lineage commitment. *Nat Cell Biol* **18**, 864-875 (2016).
16. Malinverno, C. *et al.* Endocytic reawakening of motility in jammed epithelia. *Nat Mater* **16**, 587-596 (2017).
17. Dobin, A. *et al.* STAR: ultrafast universal RNA-seq aligner. *Bioinformatics* **29**, 15-21 (2013).
18. Love, M.I., Huber, W. & Anders, S. Moderated estimation of fold change and dispersion for RNA-seq data with DESeq2. *Genome Biol* **15**, 550 (2014).
19. Sergushichev, A.A. An algorithm for fast preranked gene set enrichment analysis using cumulative statistic calculation. *bioRxiv*, 060012 (2016).
20. Della Chiara, G. *et al.* Epigenomic landscape of human colorectal cancer unveils an aberrant core of pan-cancer enhancers orchestrated by YAP/TAZ. *Nat Commun* **12**, 2340 (2021).
21. He, D.C., Nickerson, J.A. & Penman, S. Core filaments of the nuclear matrix. *J Cell Biol* **110**, 569-580 (1990).

22. Bolger, A.M., Lohse, M. & Usadel, B. Trimmomatic: a flexible trimmer for Illumina sequence data. *Bioinformatics* **30**, 2114-2120 (2014).
23. Li, H. & Durbin, R. Fast and accurate short read alignment with Burrows-Wheeler transform. *Bioinformatics* **25**, 1754-1760 (2009).
24. Li, H. *et al.* The Sequence Alignment/Map format and SAMtools. *Bioinformatics* **25**, 2078-2079 (2009).
25. Kharchenko, P.V., Tolstorukov, M.Y. & Park, P.J. Design and analysis of ChIP-seq experiments for DNA-binding proteins. *Nat Biotechnol* **26**, 1351-1359 (2008).
26. Hahne, F. & Ivanek, R. Visualizing Genomic Data Using Gviz and Bioconductor. *Methods Mol Biol* **1418**, 335-351 (2016).
27. Ramirez, F. *et al.* deepTools2: a next generation web server for deep-sequencing data analysis. *Nucleic Acids Res* **44**, W160-165 (2016).
28. Beznoussenko, G.V. *et al.* Transport of soluble proteins through the Golgi occurs by diffusion via continuities across cisternae. *Elife* **3** (2014).
29. Beznoussenko, G.V., Ragnini-Wilson, A., Wilson, C. & Mironov, A.A. Three-dimensional and immune electron microscopic analysis of the secretory pathway in Saccharomyces cerevisiae. *Histochem Cell Biol* **146**, 515-527 (2016).
30. Beznoussenko, G.V. & Mironov, A.A. Correlative video-light-electron microscopy of mobile organelles. *Methods Mol Biol* **1270**, 321-346 (2015).
