# Supplementary material for: Tissue fluidification promotes a cGAS–STING cytosolic DNA response in invasive breast cancer
Source: Nat Mater. 2022 Dec 29;22(5):644–55. doi: 10.1038/s41563-022-01431-x (PMC10156599; doi:10.1038/s41563-022-01431-x)
Supplement: Supplementary file 2 — Reporting Summary [file 41563_2022_1431_MOESM2_ESM.pdf]

nature portfolio

Corresponding author(s): giorgio scita

Last updated by author(s): 23/09/2022

Reporting Summary

Nature Portfolio wishes to improve the reproducibility of the work that we publish. This form provides structure for consistency and transparency in reporting. For further information on Nature Portfolio policies, see our Editorial Policies and the Editorial Policy Checklist.

Statistics

For all statistical analyses, confirm that the following items are present in the figure legend, table legend, main text, or Methods section.

n/a	Confirmed	
☐	☒	The exact sample size (*n*) for each experimental group/condition, given as a discrete number and unit of measurement
☐	☒	A statement on whether measurements were taken from distinct samples or whether the same sample was measured repeatedly
☐	☒	The statistical test(s) used AND whether they are one- or two-sided<br>*Only common tests should be described solely by name; describe more complex techniques in the Methods section.*
☒	☐	A description of all covariates tested
☐	☒	A description of any assumptions or corrections, such as tests of normality and adjustment for multiple comparisons
☐	☒	A full description of the statistical parameters including central tendency (e.g. means) or other basic estimates (e.g. regression coefficient) AND variation (e.g. standard deviation) or associated estimates of uncertainty (e.g. confidence intervals)
☐	☒	For null hypothesis testing, the test statistic (e.g. *F*, *t*, *r*) with confidence intervals, effect sizes, degrees of freedom and *P* value noted<br>*Give P values as exact values whenever suitable.*
☒	☐	For Bayesian analysis, information on the choice of priors and Markov chain Monte Carlo settings
☒	☐	For hierarchical and complex designs, identification of the appropriate level for tests and full reporting of outcomes
☒	☐	Estimates of effect sizes (e.g. Cohen's *d*, Pearson's *r*), indicating how they were calculated

Our web collection on statistics for biologists contains articles on many of the points above.

Software and code

Policy information about availability of computer code

Data collection

- Zen 2.0 Software
- Image Lab software (v 3.0)
- 7500 software (v 2.0.6)
- Metamorph (v 7.8)
- 3DMOD 4.0.11
- Leica Application Suite X
- Harmony software v 4.9.
- Leica LAS AF
- NanoWizard Control Software v.6

Data analysis

- Fiji (v 2.3.0)
- MatLab (v R2021b)
- GraphPad PRISM (9.4.1)
- Excel (16.62)
- AFM (Floriana)
- Samtools (1.16.1)
- Picard (v2.22)
- DeepTools (v 3.4.3)
- SPP (v 1.16.0)
- R (v 3.5.2)
- Gviz (1.26.5)

- Bioconductor package DESeq2 (for DGE) version 1.36.0
- Bioconductor package fgsea (v 1.22.0)
- Transcription Factor Enrichment Analysis tool from the X2K Web suite
- Trimmomatic (v 0.39)
- JPK Data Processing Version 6.1.116

All custom code and scripts used in this study are available online: https://doi.org/10.5281/zenodo.7117355 : Matlab for Nuclei dynamics and mechanics; https://doi.org/10.5281/zenodo.7124712 : Wound Healing analysis tool; https://doi.org/10.5281/zenodo.7115350 : plugins Fiji for micronuclei, yH2AX foci, H3K27me3 on NE or upon request from the corresponding authors.

For manuscripts utilizing custom algorithms or software that are central to the research but not yet described in published literature, software must be made available to editors and reviewers. We strongly encourage code deposition in a community repository (e.g. GitHub). See the Nature Portfolio guidelines for submitting code & software for further information.

Data

Policy information about availability of data

All manuscripts must include a data availability statement. This statement should provide the following information, where applicable:

- Accession codes, unique identifiers, or web links for publicly available datasets
- A description of any restrictions on data availability
- For clinical datasets or third party data, please ensure that the statement adheres to our policy

RNA-seq data of MCF10A, MCF10.DCIS.com cells and organoids are deposited in the Gene Expression Omnibus (GEO) and European Genomephenome Archive (EGA), with the respective accession numbers: GSE183479 RNA-seq, GSE183539 SAMMY-seq, GSE183407 ChIP-seq, GSE205108 RNA-seq of Organoids. Other data generated or analysed during this study are provided as Source Data or included in the Supplementary Information and are available from the corresponding authors upon request.

Field-specific reporting

Please select the one below that is the best fit for your research. If you are not sure, read the appropriate sections before making your selection.

☒ Life sciences ☐ Behavioural & social sciences ☐ Ecological, evolutionary & environmental sciences

For a reference copy of the document with all sections, see nature.com/documents/nr-reporting-summary-flat.pdf

Life sciences study design

All studies must disclose on these points even when the disclosure is negative.

Sample size	No statistical methods were used to pre-determine sample sizes, which were chosen based on our previous experience on collective cell migration in 2D and 3D (e.g. Nat Mater (2017) 16, 587-596; Nat Mater (2019) 18, 1252-1263; Eur Phys J E Soft Matter 2022 45, 50) and in vivo work on cell endocytosis and tumor dissemination (e.g. J Cell Biol (2014) 206, 307-328; Nat Mater (2019) 18, 1252-1263). The exact replication numbers, sample sizes and statistical methods are described in detail in the text.
Data exclusions	No data were excluded
Replication	Data were successfully replicated in at least 3 independent experiments
Randomization	In all experiments involving mice, we assigned each mouse randomly to the treatment groups (Injection of control or RAB5A-cells into mammary fat pads). For the experiment with cells, we had two genetically distinct groups (control vs RAB5A) that were treated equally and randomly. For the CLEM experiment, we selected blindly control or RAB5A cells displaying accumulated perinuclear cGAS.
Blinding	The experiments were evaluated blindly by the operators

Reporting for specific materials, systems and methods

We require information from authors about some types of materials, experimental systems and methods used in many studies. Here, indicate whether each material, system or method listed is relevant to your study. If you are not sure if a list item applies to your research, read the appropriate section before selecting a response.

Materials & experimental systems

n/a	Involved in the study	
[ ]	[x]	Antibodies
[ ]	[x]	Eukaryotic cell lines
[x]	[ ]	Palaeontology and archaeology
[ ]	[x]	Animals and other organisms
[ ]	[x]	Human research participants
[x]	[ ]	Clinical data
[x]	[ ]	Dual use research of concern

Methods

n/a	Involved in the study	
[ ]	[x]	ChIP-seq
[x]	[ ]	Flow cytometry
[x]	[ ]	MRI-based neuroimaging

Antibodies

Antibodies used

Antibodies (The list below is also included in the supplementary tables section):

Anti LaminB1   Abcam Cat# ab16048 1:1000 WB
Anti LaminA/C (636) Santa Cruz Biotechnology Cat# ab7292 1:500 WB
Anti STAT2 Thermofisher Cat# ab44-362G 1:500 WB
Anti ISG15 Cell Signalling Technology Cat# ab2743 1:1000 WB
Anti Vinculin Sigma-Aldrich Cat# V9131 1:1000 WB
Anti RAB5A Santa Cruz Biotechnology Cat# ab309 1:500 WB
Anti RAB5A Abcam Cat# ab109534 1:100 IHC
Anti p-STAT1 (58D6) (Tyr701) Cell Signalling Technology Cat# ab9167 1:1000 WB
Anti STAT1 (42H3) Cell Signalling Technology Cat# ab9175 1:1000 WB
FITC-conjugated Phalloidin Sigma-Aldrich Cat# P5282 1:50 IF
Anti IRF3 (D614C)XP Cell Signalling Technology Cat# 11904 1:1000 WB
Anti STING/TMEM173 Novus Biologicals Cat# NBP2-24683 1:500 WB
Anti cGAS (D1D3G) Cell Signalling Technology Cat # 15102 1:100 IHC (1:1000 WB)
Anti IFIT1 (D2X9Z) Cell Signalling Technology Cat# 14769 1:500 WB
Anti Istone H3 trimetil (Lys27)(C36B11) Cell Signalling Technology Cat# 9733 1:1000 IF
Anti fosfo gH2AX S139 (20E3) Cell Signalling Technology Cat# 9718 1:500 IHC
Anti-Histone H3 (tri methyl K9) antibody Abcam Cat# 8898 3mg for CHIP
Recombinant Anti-Histone H3 (tri methyl K9) antibody Abcam Cat# ab176916 3mg for CHIP
Anti 53BP1 Abcam Cat# 175933 1:100 IF
Anti p-CHK1 (phospho S345) Abcam Cat# ab58567 1:500 IHC
Anti α–Tubulin Sigma-Aldrich Cat# T5168 1:1000 WB
Anti fosfo gH2AX S139 (20E3) Abcam Cat# 11174 1:1000 IHC
Anti cGAS (clone D1D3G) Cell Signalling Technology Cat#15102 1:100 IHC
Anti SUN2 Abcam Cat# ab124916 1:100 IF

Secondary Antibody (Goat Anti-Rabbit Antibody Conjugated to Horseradish Peroxidase) Cell Signalling Technology Cat# 7074 1:3000
Secondary Antibody (Goat Anti-Mouse Antibody Conjugated to Horseradish Peroxidase) Cell Signalling Technology Cat# 7076 1:3000
DAPI Thermofisher Cat# D-1306 1:5000
Hoechst Thermofisher Cat# 62249 1: 20
IncuCyte® NucLight Rapid Red dye Sartorius Cat# 4717 1:1000
MitoTraker Red CMXRos Thermofisher Cat# M7512 100 nM
Cy3 AffiniPure Donkey Anti-Rabbit IgG (H+L) Jackson ImmunoResearch Cat# 711-165-152 1:400
Cy3 AffiniPure Donkey Anti-Mouse IgG (H+L) Jackson ImmunoResearch Cat# 715-165-150 1:400
Donkey anti-Rabbit IgG (H+L) Highly Cross-Adsorbed Secondary Antibody, Alexa Fluor 488 Thermofisher Cat# A32790 1:100
Donkey anti-Mouse IgG (H+L) Highly Cross-Adsorbed Secondary Antibody, Alexa Fluor 488 Thermofisher Cat# A21202 1:100

Validation

The specificity of the cGAS antibody for IHC was validated previously by cGAS specific silencing siRNA.

Anti LaminB1   https://www.abcam.com/lamin-b1-antibody-nuclear-envelope-marker-ab16048.html
Anti LaminA/C (636) https://www.scbt.com/p/lamin-a-c-antibody-636
Anti STAT2 https://www.thermofisher.com/antibody/product/STAT2-Antibody-Polyclonal/44-362G
Anti ISG15 https://www.cellsignal.com/products/primary-antibodies/isg15-antibody/2743
Anti Vinculin https://www.sigmaaldrich.com/IT/it/product/sigma/v9131
Anti RAB5A NOT more disposable
Anti RAB5A https://www.abcam.com/rab5-antibody-epr5438-early-endosome-marker-ab109534.html
Anti p-STAT1 (58D6) (Tyr701) https://www.cellsignal.com/products/primary-antibodies/phospho-stat1-tyr701-58d6-rabbit-mab/9167
Anti STAT1 (42H3) https://www.cellsignal.com/products/primary-antibodies/stat1-42h3-rabbit-mab/9175
FITC-conjugated Phalloidin https://www.sigmaaldrich.com/IT/it/product/sigma/p5282
Anti IRF3 (D614C)XP https://www.cellsignal.com/products/primary-antibodies/irf-3-d6i4c-xp-rabbit-mab/11904
Anti STING/TMEM173 https://www.novusbio.com/products/sting-tmem173-antibody_nbp2-24683
Anti cGAS (D1D3G) https://www.cellsignal.com/products/primary-antibodies/cgas-d1d3g-rabbit-mab/15102
Anti IFIT1 (D2X9Z) https://www.cellsignal.com/products/primary-antibodies/ifit1-d2x9z-rabbit-mab/14769
Anti Istone H3 trimetil (Lys27)(C36B11) https://www.cellsignal.com/products/primary-antibodies/tri-methyl-histone-h3-lys27-

c36b11-rabbit-mab/9733
Anti fosfo gH2AX S139 (20E3) https://www.cellsignal.com/products/primary-antibodies/phospho-histone-h2a-x-ser139-20e3-rabbit-mab/9718
Anti-Histone H3 (tri methyl K9) antibody https://www.abcam.com/products?keywords=8898
Recombinant Anti-Histone H3 (tri methyl K9) antibody https://www.abcam.com/products?keywords=176916
Anti 53BP1 https://www.abcam.com/products?keywords=+175933
Anti p-CHK1 (phospho S345) https://www.abcam.com/chk1-phospho-s345-antibody-ab58567.html
Anti α–Tubulin https://www.sigmaaldrich.com/IT/it/product/sigma/t5168
Anti fosfo gH2AX S139 (20E3) https://www.abcam.com/products?keywords=11174
Anti cGAS (clone D1D3G) https://www.cellsignal.com/products/primary-antibodies/cgas-d1d3g-rabbit-mab/15102
Anti SUN2 https://www.abcam.com/products?keywords=124916

Secondary Antibody (Goat Anti-Rabbit Antibody Conjugated to Horseradish Peroxidase) https://www.cellsignal.com/products/secondary-antibodies/anti-rabbit-igg-hrp-linked-antibody/7074
Secondary Antibody (Goat Anti-Mouse Antibody Conjugated to Horseradish Peroxidase) https://www.cellsignal.com/products/secondary-antibodies/anti-mouse-igg-hrp-linked-antibody/7076
DAPI https://www.thermofisher.com/order/catalog/product/D1306?SID=srch-srp-D1306
Hoechst https://www.thermofisher.com/order/catalog/product/62249?SID=srch-srp-62249
IncuCyte® NucLight Rapid Red dye https://www.sartorius.com/en/search?search=4717#24598_24596=4717
MitoTraker Red CMXRos https://www.thermofisher.com/order/catalog/product/M7512
Cy3 AffiniPure Donkey Anti-Rabbit IgG (H+L) https://www.jacksonimmuno.com/catalog/products/711-165-152
Cy3 AffiniPure Donkey Anti-Mouse IgG (H+L) https://www.jacksonimmuno.com/catalog/products/715-165-150
Donkey anti-Rabbit IgG (H+L) Highly Cross-Adsorbed Secondary Antibody, Alexa Fluor 488 https://www.thermofisher.com/antibody/product/Donkey-anti-Rabbit-IgG-H-L-Highly-Cross-Adsorbed-Secondary-Antibody-Polyclonal/A32790
Donkey anti-Mouse IgG (H+L) Highly Cross-Adsorbed Secondary Antibody, Alexa Fluor 488 https://www.thermofisher.com/antibody/product/Donkey-anti-Mouse-IgG-H-L-Highly-Cross-Adsorbed-Secondary-Antibody-Polyclonal/A-21202

Eukaryotic cell lines

Policy information about cell lines

Cell line source(s)

MCF10.DCIS.com cells were provided by J. F. Marshall (Barts Cancer Institute, Queen Mary University of London, UK) and maintained in DMEM/F12 (Biowest) supplemented with 5% horse serum (Life Technologies), 2 mM L-Glutamine (EuroClone), 0.5 mg/ml Hydrocortisone (Sigma-Aldrich), 10 µg/ml Human insulin (Sigma-Aldrich) and 20 ng/ml EGF (Peprotech).
MCF10A cells were a gift from J. S. Brugge (Department of Cell Biology, Harvard Medical School, Boston, USA), originally obtained from ATCC and were maintained in DMEM/F12 (Biowest) supplemented with 5% horse serum (Life Technologies), 2 mM L-Glutamine (EuroClone), 0.5 mg/ml Hydrocortisone (Sigma-Aldrich), 100 ng/ml cholera toxin (Sigma-Aldrich), 10 µg/ml Human insulin (Sigma-Aldrich) and 20 ng/ml EGF (Peprotech).
HaCaT (ATCC) were maintained DMEM (Lonza) + 10% FBS (Life Technologies) + 2 mM L-Glutamine (EuroClone).
Phoenix-AMPHO cells (American Type Culture Collection, CRL-3213) were used as the packaging cell line for the generation of retroviral particles and cultured as recommended by the supplier.
HEK293T (BBCF-Biological Bank and Cell factory, INT, Milan) were grown in DMEM (Lonza) supplemented with 10% FBS (Life Technologies) and 2 mM L-Glutamine (EuroClone) and used as the packaging line for lentiviral vectors.

Authentication

The identity of each of the cell line was verified by fingerprinting by our cell culture facility.

Mycoplasma contamination

Each of the cell line is tested negative for Mycoplasma by our cell culture facility.

Commonly misidentified lines
(See ICLAC register)

No commonly misidentified cell lines were used.

Animals and other organisms

Policy information about studies involving animals; ARRIVE guidelines recommended for reporting animal research

Laboratory animals

We employed immuno-compromised mice; Strain NOD.Cg-PrkdcscidIl2rgtm1Wjl/SzJ (commonly known as the NOD SCID gamma; NSG). The animals were housed at 20-24 degrees in controlled humidity ranging from 45 to 60% and with a12 hours light cycle.

Methods used with these animals:
Control and RAB5A MCF10.DCIS.com were injected into female NOD.Cg-PrkdcscidIl2rgtm1Wjl/SzJ (commonly known as the NOD SCID gamma; NSG) mice as described previously99. Before injection, control or RAB5A-expressing MCF10.DCIS.com cells were trypsin detached, washed twice and resuspended at a final concentration of 300000 cells/13 µl PBS. The cell suspension was then mixed with 5 µl growth factor–reduced Matrigel (BD, Cat# 354263) and 2 µl Trypan blue solution and maintained on ice until injection. Aseptic conditions under a laminar flow hood were used throughout the surgical procedure. Female NSG mice, 6–9 weeks-old, were anesthetized with 2% isoflurane and injected with a 20µl cell suspension directly in the fourth mammary fad pad. Mice were fed with doxycycline hyclate 4 days after injection. Tumor growth was monitored weekly using digital calipers, and tumor volume was calculated according to the formula: L × W2/2 = mm3. After 3 weeks, mice were anesthetized with 2% isoflurane to remove primary tumors (mastectomy). For histological evaluation, primary tumors were fixed in 4% phosphate-buffered formalin and embedded in paraffin. 3-µm sections of tumors were made and slides were counterstained with H&E and with indicated antibodies. Fragments of primary tumors were snap frozen on dry ice and stored at −80°C before mRNA analysis. For ex vivo MCF10.DCIS.com tumor slices, primary tumors were removed, cut by a scalpel and each tumor slide was placed over a metal grid inserted in a six-well plate to allow tumors to grow on an interface air/culture medium. Before imaging, 2.5 µg/ml doxycycline hyclate was added to the tumor slices

culture media to induce RAB5A expression. Tumor cells were maintained under stimulation for five days, changing the medium every day. Tissue samples were fixed in 10% buffered formalin and embedded in paraffin. 4-µm tissue sections were counterstained with H&E and with indicated antibodies.

Wild animals

No wild animals were used in the study.

Field-collected samples

No field-collected samples were used in the study.

Ethics oversight

All animal experiments were approved by the OPBA (Organisms for the well-being of the animal) of IFOM and Cogentech. All experiments complied with national guidelines and legislation for animal experimentation. All mice were bred and maintained under specific pathogen-free conditions in our animal facilities at Cogentech Consortium at the FIRC Institute of Molecular Oncology Foundation and at the European Institute of Oncology in Milan, under the authorization from the Italian Ministry of Health (Autorizzazione N° 604-2016).

Note that full information on the approval of the study protocol must also be provided in the manuscript.

Human research participants

Policy information about studies involving human research participants

Population characteristics

Breast biopsies were collected from women undergoing mastectomy for primary breast cancer at The EUROPEAN INSTITUTE OF ONCOLOGY, MILAN. Donors were informed before the surgery and agreed by written consent to donate tissues. Tumor biopsies were processed immediately upon receipt and cultured as described below. The following samples were used

Sample N.	TYPE	TISSUE TYPE	DIAGNOSIS	ER (%)	PGR (%)	HER2 SCORE	HER2 %	KI67 (%)	AGE
BR 25	Path.	Breast - Left	Infiltrating duct carcinoma	60	60	Neg		4	49
BR 30	Path.	Breast - Right	Infiltrating duct carcinoma	90	90	3+	90	28	46
BR 39	Path.	Breast - Left	Infiltrating duct carcinoma	95	95	Neg		12	50
BR 40	Path.	Breast - Right	Cribriform carcinoma	95	30	1+	15	3	56
BR 44	Path.	Breast - Right	Infiltrating duct carcinoma	95	40	Neg		22	43

Recruitment

No selection of patient material (breast cancer tissue and fresh samples) was applied.

Ethics oversight

The use of human material has been reviewed by European Institute of Oncology Ethical Committees (reference to UID 2152). The permit for obtaining clinical material did not include access to basic information regarding patients and their detailed medical histories are not given to the authors.

Note that full information on the approval of the study protocol must also be provided in the manuscript.

ChIP-seq

Data deposition

☒ Confirm that both raw and final processed data have been deposited in a public database such as GEO.

☒ Confirm that you have deposited or provided access to graph files (e.g. BED files) for the called peaks.

Data access links
May remain private before publication.

GEO datasets are available at:

GSE183407 ChIP-Seq
GSE205108 RNA-seq of Organoids
GSE183479 RNA-seq
GSE183539 SAMMY-seq

Files in database submission

Raw sequencing data files (FASTQ), Bigwig files for genomics tracks and BED files for peak calls

Genome browser session
(e.g. UCSC)

N/A

Methodology

Replicates

H3K9me3 ChIP-seq experiments were replicated with two different antibodies.

Sequencing depth

Sequencing was performed in SR mode (1x75nt) on an Illumina NextSeq550 platform, generating on average 35 million SR reads per

Sequencing depth	sample.
Antibodies	H3K4me3 Millipore 07-473, H3K27Ac abcam 4729, H3K9me3 ab176916, H3K9me3 ab8898. Immunocomplexes were recovered with 20 µl of pre-blocked Protein G-Dynabeads (Thermo Fisher) for 2 h, at 4°C, and washed twice with RIPA-low salt, twice with RIPA-high salt, twice with RIPA-LiCl and once with 10 mM Tris pH 8.0 and once with 1X TE, as previously reported90. The washed immunocomplexes were incubated with ChIP elution buffer (10 mM Tris-HCl pH 8.0, 5 mM EDTA pH 8.0, 300 mM NaCl, 0.4% SDS) supplemented with 0.8 mg/ml Proteinase K for 1 h at 55°C and overnight at 65°C, for reverse crosslinking. The immunoprecipitated DNA was then purified by Qiagen MinElute kit (Qiagen) and eluted in 22 µl EB buffer. ChIP-seq libraries were constructed with TruSeq ChIP Library Preparation Kit (Illumina), according to the manufacturer's instructions and sequenced on Illumina HiSeq2500 platform.
Peak calling parameters	Heterochromatin (H3K9me3 enriched) domains were defined using the EDD (v1.1.19) software with parameters (binsize = 200 Kb and gap penalty = 25) processing the filtered bam files obtained as described above. The "required_fraction_of_informative_bins" parameter was set to 0.98. The unalignable regions were defined with the ENCODE Unified GRCh38 Exclusion List (previously "blacklist") and downloaded from (https://www.encodeproject.org/files/ENCFF356LFX/).
Data quality	Sequencing reads were trimmed and adapters removed by using Trimmomatic (v0.39)92 using the following parameters for SAMMY-seq and ChIP-seq data: 2 for seed_mismatch, 30 for palindrome_threshold, 10 for simple_threshold, 3 for leading, 3 for trailing and 4:15 for sliding window and sequence minimum length threshold of 35. As clip file has been used the trimmomatic provided dataset "TruSeq3-SE.fa" (for single end).<br>After trimming, the reads were aligned using BWA (v0.7.17-r1188)93 etting –k parameter as 2 and using as reference genome the UCSC hg38 one (only canonical chromosomes have been taken into consideration). The alignment duplicates have been marked with Picard (v2.22) (http://broadinstitute.github.io/picard/) MarkDuplicates option. And then filtered using Samtools (v1.9)94, in addition we filtered all the reads with mapping quality lower than 1, unmapped and read fails platform/vendor quality checks (-F 1540 -q 1). Each sequencing lane has been analysed separately up to this point and then merged.
Software	We used the following software (with respective version numbers) in the preprocessing and analysis of ChIP-seq data: Trimmomatic (v0.39); BWA (v0.7.17-r1188); Picard (v2.22); Samtools (v1.9); R (v3.5.2); SPP (v1.16.0); Gviz (1.26.5); EDD (v1.1.19); DeepTools (v3.4.3)
