# Supplementary figures and images for: Tissue fluidification promotes a cGAS–STING cytosolic DNA response in invasive breast cancer
Source: Nat Mater. 2022 Dec 29;22(5):644–55. doi: 10.1038/s41563-022-01431-x (PMC10156599; doi:10.1038/s41563-022-01431-x)

Source data-Uncropped scanned blots Fig. 1E

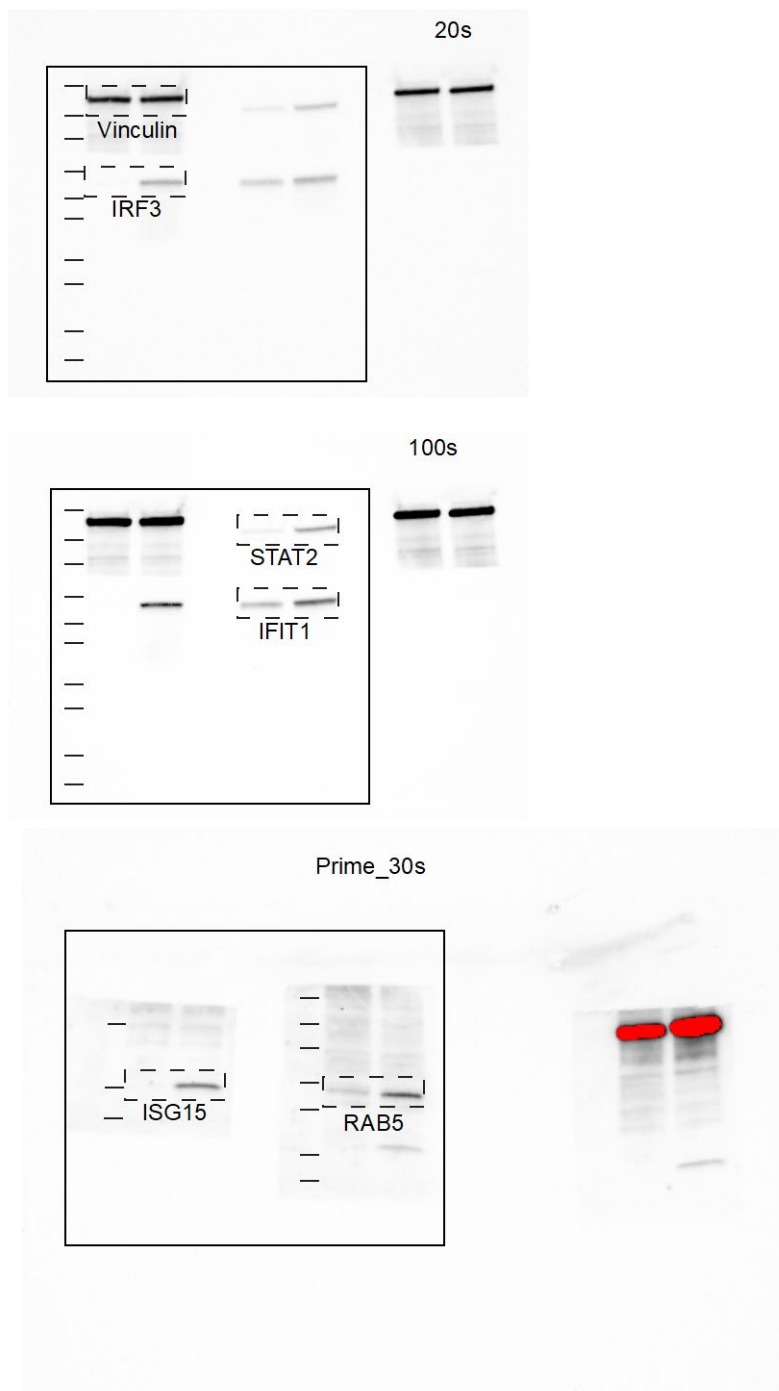

Supplement: Source Data Fig. 1 — Uncropped scanned blots. [file 41563_2022_1431_MOESM18_ESM.pdf]

Source data-Uncropped scanned blots Extended data Fig. 1C

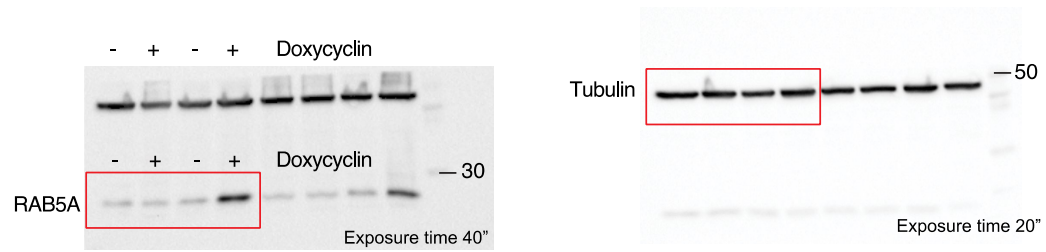

Supplement: Source data. Extended Data Fig. 1c — Uncropped scanned blots. [file 41563_2022_1431_MOESM26_ESM.pdf]
