# Supplementary material for: Tissue fluidification promotes a cGAS–STING cytosolic DNA response in invasive breast cancer
Source: Nat Mater. 2022 Dec 29;22(5):644–55. doi: 10.1038/s41563-022-01431-x (PMC10156599; doi:10.1038/s41563-022-01431-x)

Source data-Uncropped scanned blots Fig. 2D

Figure 2D

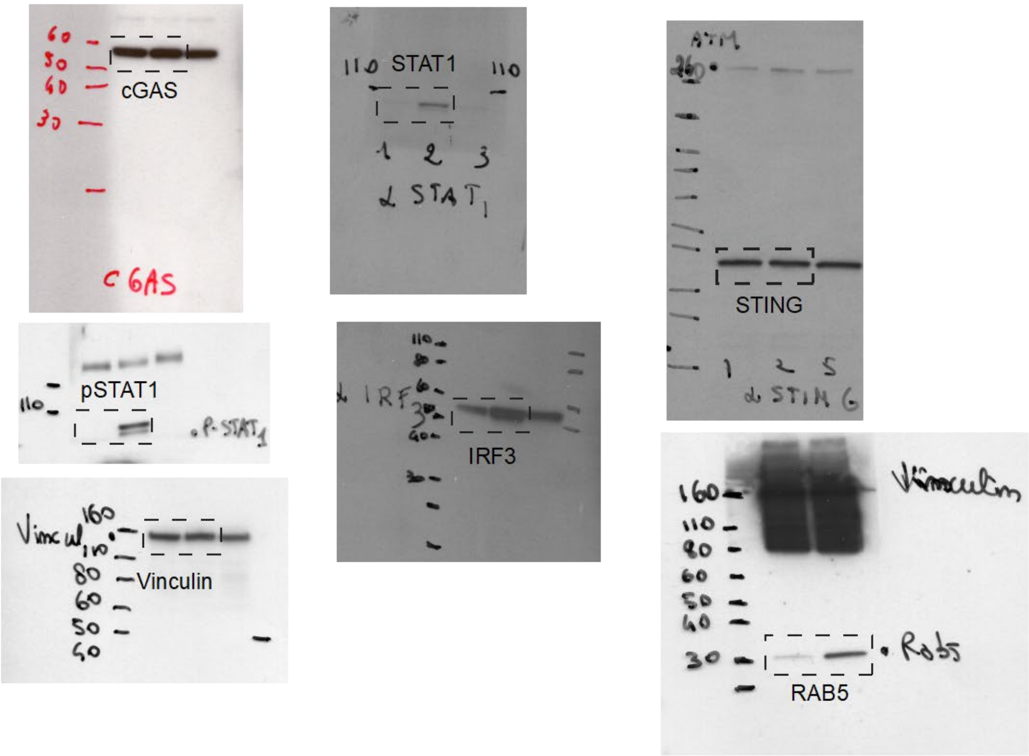

Source data-Uncropped scanned blots Fig. 2K

Figure 2K

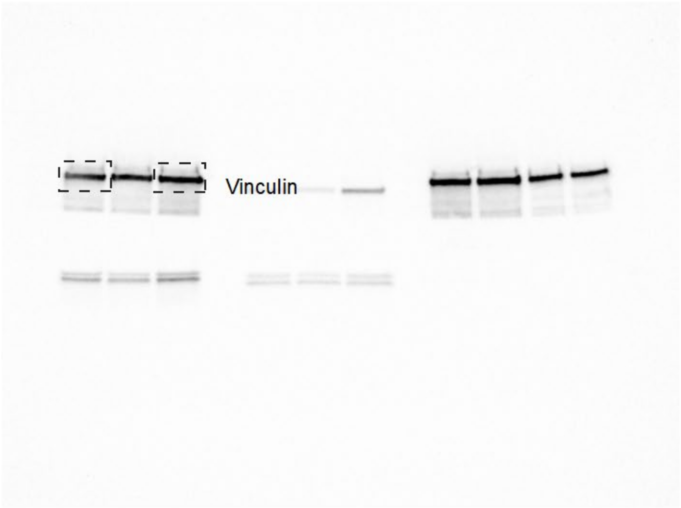

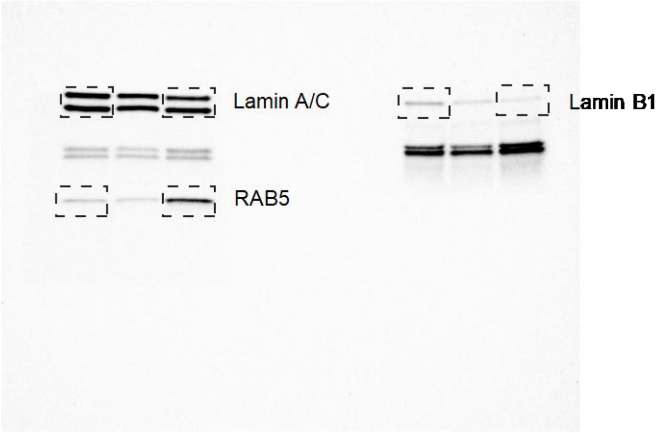

Supplement: Source Data Fig. 2 — Uncropped scanned blots. [file 41563_2022_1431_MOESM20_ESM.pdf]
